# Sabinene: A New Green Solvent Used in the Synthesis of Thiazolo[5,4-*b*]pyridines by Thermal or Microwave Activation

**DOI:** 10.3390/molecules28196924

**Published:** 2023-10-03

**Authors:** Gatien Messire, Véronique Ferreira, Emma Caillet, Lyana Bodin, Amélia Auville, Sabine Berteina-Raboin

**Affiliations:** Institut de Chimie Organique et Analytique (ICOA), Université d’Orléans, UMR-CNRS 7311, BP 6759, rue de Chartres, 45067 Orléans, France; gatien.messire@univ-orleans.fr (G.M.); veronique.ferreira-bardin@univ-orleans.fr (V.F.); emma.caillet@etu.univ-orleans.fr (E.C.); lyana.bodin@etu.umontpellier.fr (L.B.); amelia.auville@etu.univ-orleans.fr (A.A.)

**Keywords:** sustainable chemistry, bicyclic *N*,*S*-heterocycles, biomass-based green solvents, sabinene

## Abstract

Following the work already carried out in our laboratory on eucalyptol, a new green solvent derived from biomass, we are now looking at sabinene as another new green solvent. Sabinene is also derived from biomass, has no known toxicity and can be recycled by distillation. We have shown that it can be used as it is or distilled to synthesize thiazolo[5,4-*b*]pyridine heterocycles by thermal activation or microwave irradiation. This new solvent was compared with various conventional and green solvents. The conditions were optimised to enable us to carry out the syntheses in satisfactory yields, and we were able to show that sabinene, a natural bicyclic monoterpene, could be used effectively as a solvent.

## 1. Introduction

**Figure 1:**
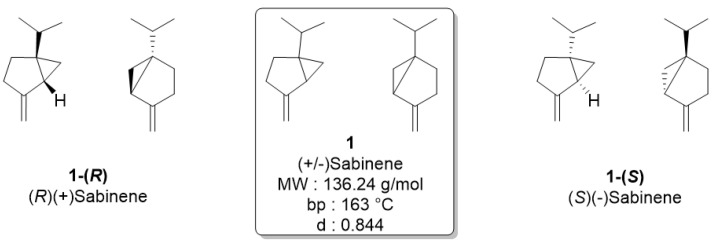
Sabinene structure.

Organic chemistry mainly uses petroleum-based products or solvents, which have a major impact on the environment. Today, it is important to preserve our non-renewable resources by using new types of solvents derived from biomass and to think about the economy of the atom. In 2019, we were able to highlight a new solvent derived from biomass, eucalyptol [1]. This has been compared with known conventional and green solvents and has shown an undeniable interest in the organic synthesis of numerous nitrogenous and sulphurous oxygenated heterocyclic compounds via, in particular, nucleophilic substitutions, cyclisations, various metal-catalysed couplings and multicomponent reactions [1,2,3,4,5]. Although we are still applying this new solvent in various synthetic processes, within the team we are continuing our efforts to limit our environmental impact and are therefore looking at other biomass-derived solvents. Sabinene **1** (Figure 1) is a compound in the family of unsaturated monoterpene hydrocarbons with the molecular formula C_10_H_16_. Sabinene is its trivial name, while its IUPAC name is 4-methylidene-1-(propan-2-yl)bicyclo[3.1.0]hexane. It is classified as a food additive and as a flavouring agent in the perfume industry. It is known for its anti-inflammatory, antioxidant, antifungal, [6,7], antiseptic, antimicrobial [8] and bactericidal properties [9].

Sabinene is either extracted from various plants or biosynthesised by enzymatic reaction [10]. It is naturally present in juniper (*Juniperus Sabina*) [11], marjoram (*Origanum majorana)* [12], holm oak (*Quercus ilex*) [13], Norway spruce (*Picea abies*), Douglas fir (*Pseudotsuga menziesii*) [14], spearmint (*Mentha spicata*)[15], angelica (*Angelica archangelica*, *Apiaceae*) [16], carrots (*Daucus Carota*) [17], black pepper (*Piperaceae*) [18], Clausena anisata (Wildd.) Hook.f. ex Benth. (*Rutacea*) [19] or the citrus family [7] and many others. Sabinene, present in certain citrus fruits, could therefore be obtained from waste products from the fruit juice industry. It was therefore interesting to test it for the organic synthesis of compounds for biological purposes, as its use as a solvent would contribute to the recycling of industrial waste.

Sabinene has also been reported as a starting material for advanced biofuels [20,21]. Here, it is used as a green solvent for the synthesis of various thiazolo[5,4-*b*]pyridines compared to eucalyptol or cyclopentyl methyl ether (CPME), limonene and citral.

On the one hand, heterocycles are widely present in many agrochemical and pharmaceutical products [22,23,24,25,26,27,28]. To date, the number of pharmaceutical products containing a heterocyclic part in their skeleton, and in particular bicyclic heterocycles, is estimated to be around 70%, hence the importance of mastering synthesis protocols and carrying them out under the safest possible conditions, for the development and production of new environmentally friendly drugs or agrochemical compounds [25,29,30,31]. On the other hand, thiazolo[5,4-*b*]pyridine analogues are known for their promising properties and are therefore the subject of various developments [32,33,34,35,36], particularly in oncology, as some analogues show very good inhibition (in the nanomolar range) of phosphoinositide 3-kinase (PI3K) [37]. This is an important target for survival, proliferation and differentiation, and therefore for targeted tumour therapy [38]. These compounds can be synthesised in several ways, depending on the functionalities envisaged, in particular on the 6-membered ring. They can also be synthesised in a single step from a chloronitropyridine and a suitably substituted thioamide or thiourea [39]. We chose to use the one-step method starting from a 3-amino-2-chloropyridine derivative and an isothiocyanate, a synthetic method already used when we investigated laser irradiation as a new activation method in organic synthesis [40]. This reaction was chosen because of the interest of this type of heterocycle, but also because while the reagents are soluble, the product precipitates out of the medium, making it easy to visualise its production.

## 2. Results and Discussion

### 2.1. Thiazolo-Pyridine Synthesis in Various Standard and Green Solvents 

#### 2.1.1. Optimisation in Various Solvents

On the basis of the results obtained in a previous study [40] involving coupling between 3-amino-2-chloropyridine **2a** and phenyl isothiocyanate **3a** to obtain N-phenylthiazolo[5,4-*b*]pyridin-2-amine **4a**, the synthesis was first carried out in various conventional solvents before being performed in green solvents. To investigate the ranges and limits, the temperature was maintained at a set point of 110 °C for an internal temperature of 100 °C in a sealed tube for each experiment by conventional heating using a stirring plate. It was found that 4 h were required at this temperature in conventional solvents, and that increasing the reaction time did not provide any significant improvement. It should be noted that the product obtained is the HCl salt product already described by Atland and Molander [41], which exhibits a characteristic NMR spectrum. We tried to carry out the reaction in the presence of a base equivalent such as K_2_CO_3_, but the reaction proved inefficient under these conditions. The solution was to proceed in two stages, forming the product in salt form and then neutralising it in the presence of a base. We therefore continued our study without a base and formed the products in salt form.

As the yields obtained were moderate in both conventional and green solvents, we concentrated on the latter to optimise reaction time, using one equivalent of each reagent. While citral only led to a disappointing yield of 21% in 16 h, increasing the reaction time was beneficial for the other green solvents, allowing us to achieve satisfactory yields of 58 to 75% (Table 1, entries 7,9 and 11). Beyond 16 h, we did not observe any improvement in performance. We used sabinene as a new solvent and obtained encouraging results (Table 1, entries 10 and 11), although not as good as with eucalyptol or CPME (entries 6 to 9). Citral is a compound that does not behave very well at this temperature: the medium blackens as soon as the reaction temperature reaches 95 °C, whereas its boiling point is 229 °C (Table 1, entry 14). Given this degradation of the medium, we have not studied this solvent in depth, concentrating instead on sabinene and its comparison with eucalyptol, CPME and limonene.

#### 2.1.2. Optimisation in Green Solvents

We therefore continued our optimisation in the previously mentioned green solvents before applying this new solvent (sabinene) to the synthesis of various compounds. The starting 3-amino-2-chloropyridine **2a** (1.5 mmol) was heated in 1 mL of solvent in the presence of phenyl isothiocyanate **3a** (Table 2). The yield was improved by increasing the amount of pyridine reagent (Table 2, entries 5, 9 and 12).

Since sabinene is commercially available at 75% purity (Merck, natural sabinene), we distilled it under reduced pressure with a membrane pump at 12 mbar, at 40 °C. However, we found that the reactions carried out in distilled or undistilled sabinene were unaffected and that the yields were equivalent, so we continued our study with commercially available undistilled sabinene.

**Figure 2:**
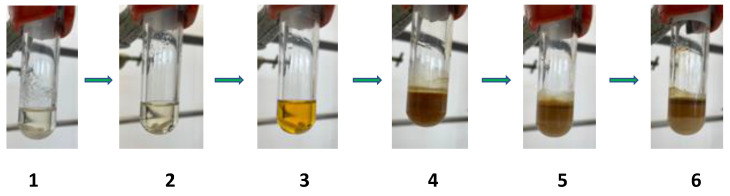
Visual evolution of a manipulation from time T = 0 at r.t. to time T = 24 h at 100 °C. **1**: T = 0, reaction medium. **2**: T = 60 °C during heating period. **3**: after 5 min at 100 °C. **4**: after 30 min at 100 °C. **5**: after 4 h at 100 °C. **6**: after 24 h at 100 °C.

To visualise the evolution of the reaction, the different phases, using 3-amino-2-chloropyridine **2a** heated in 1 mL of sabinene in the presence of 4-bromophenyl isothiocyanate, were photographed at different reaction times, starting with the control after mixing the compounds, then during the heating period at 60 °C, after 5 min at 100 °C, after 30 min at 100 °C, then after 4 h at 100 °C, and finally after 24 h of reaction at 100 °C, where complete precipitation of the product could be seen (Figure 2).

### 2.2. Optimisation of Thiazolo-Pyridine Synthesis in Sabinene

#### Under Microwave Irradiation and Thermal Conditions

Based on these initial results, the reaction was carried out under the conditions described in Table 2, entry 12, in order to obtain the optimum yield. We then tried to reduce the reaction time by using microwave activation. The temperature was also adapted. After completion, the product was filtered and washed with ethyl acetate and diethyl ether, and no further purification was required (Table 3).

Under microwave irradiation, the best results were obtained in 1 h at 160 °C, close to the boiling point of sabinene. However, as this temperature is not compatible with all isothiocyanates, we tried to reduce the reaction temperature and found that at 130 °C, the time required was 2 h. As sabinene is not a polar solvent and therefore not the most interesting for microwave reactions, we used 25% of a co-solvent that increases this polarity and chose ethanol or acetonitrile, which has proved very interesting as a co-solvent in previous work under microwave irradiation [42]. It turns out that while ethanol offers no improvement, acetonitrile halves the reaction time at 130 °C, while increasing the yield very slightly (Table 3, entries 4 and 6).

**Scheme 1:**
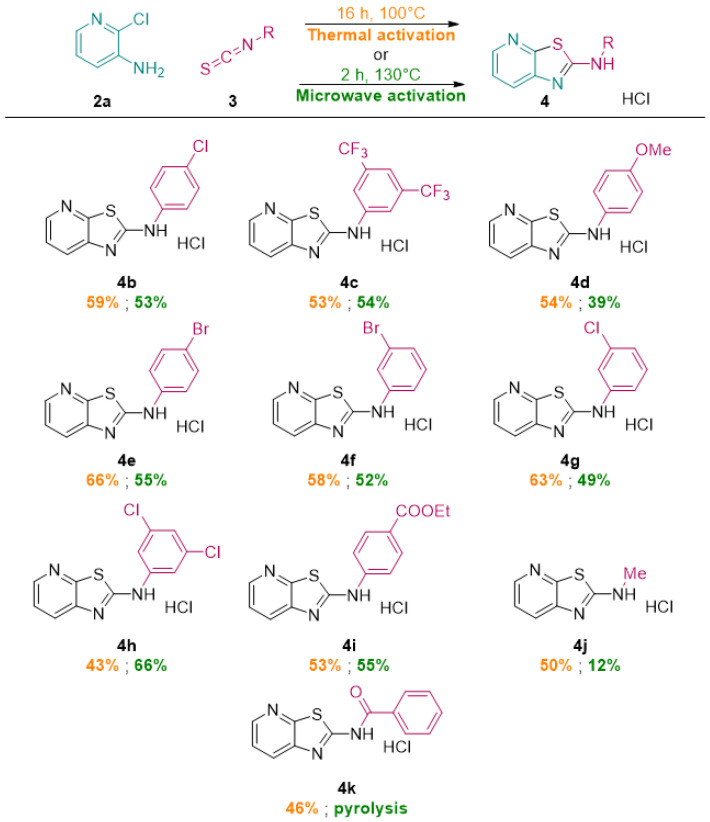
Thiazolo-pyridine synthesis in sabinene under thermal or microwave activation.

We applied these optimised conditions to a number of isothiocyanates **3** starting with 3-amino-2-chloropyridine **2a** under thermal or microwave activation. The results are summarised in Scheme 1.

Six new structures were synthesised and obtained in high yields and purity by thermal and microwave activation, the other four having already been obtained in a previous study [40]. Secondly, we investigated the use of other pyridines substituted with an alkyl-type electron donor group.

Using 3-amino-2-chloro-5-methylpyridine **2b** and phenyl isothiocyanate **3a** in a sealed tube with 1 mL of sabinene required heating to 160 °C under thermal conditions and took 16 h to achieve a satisfactory result. After the reaction, the mixture was filtered with ethyl acetate and the desired compound was synthesised in a 66% yield (Table 4, entry 5).

**Scheme 2:**
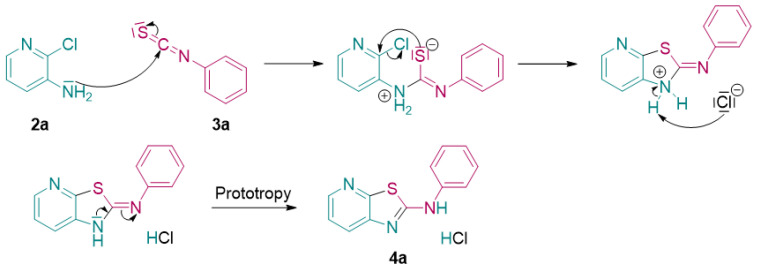
Mechanism of 2-amino thiazolo[5,4-*b*]pyridine formation.

In this case, too, we obtained the product in salt form and the reaction temperature was increased due to the donor effect of the para at the chlorine atom, which could explain the lower reactivity of the latter given the mechanism of formation of 2-aminothiazolo[5,4-*b*]pyridine (Scheme 2).

This mechanism was validated by Atland and Molander [41] with the formation of thiourea, in which the tautomeric thione or thiol displaced the chlorine atom.

Under microwave irradiation, the best results were obtained using 1.1 equiv. 3-amino-2-chloro-5-methylpyridine **2b** and 1 equiv. isothiocyanate **3**, in a sealed tube with 1 mL sabinene. The reaction was carried out at 130 °C for 2 h to give a yield of 64% (Table 5, entry 7). Again, at the end of the reaction, the mixture was filtered and rinsed with ethyl acetate.

**Scheme 3:**
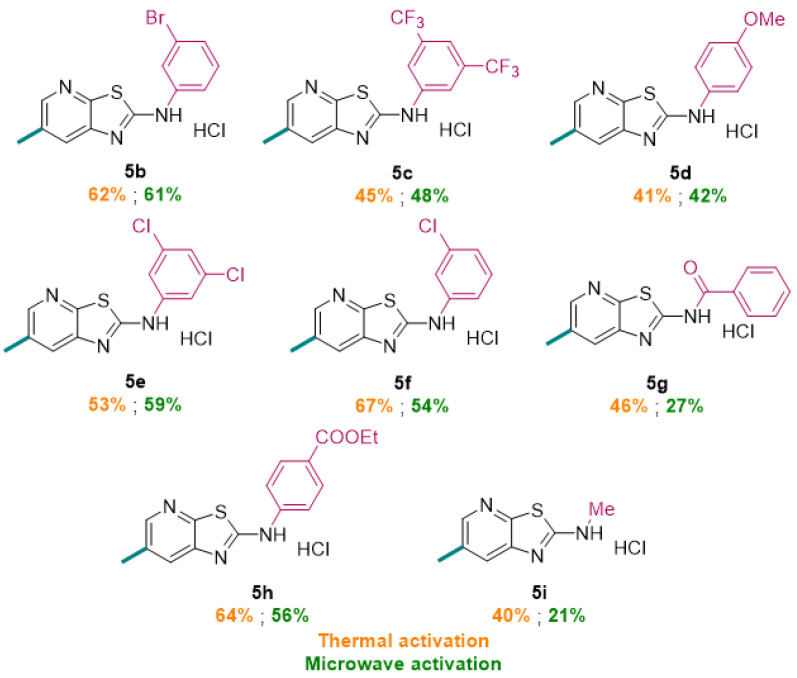
6-methyl-thiazolo-pyridine synthesis in sabinene under thermal or microwave activation.

We applied these optimised conditions to certain isothiocyanates with 3-amino-2-chloro-5-methylpyridine **2b** under thermal or microwave activation. The results are summarised in Scheme 3.

The use of 3-amino-2-chloro-5-methylpyridine **2b** gives good yields when activated by conventional heating. The results remain lower under microwave irradiation, but enable the desired products to be generated more quickly. We were able to overcome the deactivating effect of methyl in this reaction by adjusting the conditions.

**Scheme 4:**
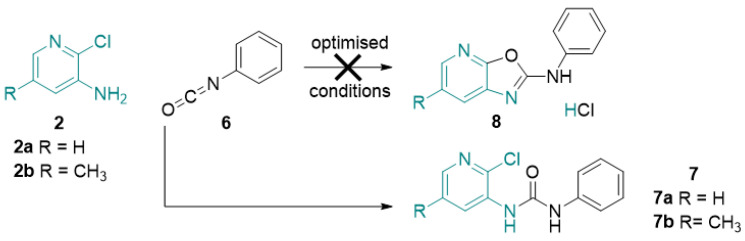
Synthesis of corresponding urea instead of desired oxazolopyridine.

In parallel, we tested these conditions using phenyl isocyanate **6** to obtain the corresponding oxazolopyridines **8**. However, as reported by Sun and co-workers [43], in this case and under our conditions, we also stopped at urea **7** (Scheme 4).

## 3. Materials and Methods

### 3.1. General Information

All reagents were purchased from commercial suppliers and used without further purification. Natural sabinene was purchased from Merck (KGaA, Darmstadt, Germany) with 75% of purity. Unless otherwise specified, sabinene was used in its commercial form. ^1^H and ^13^C NMR spectra were recorded on a Bruker DPX 250 (^13^C, 62.9 MHz) (Bruker, Wissembourg, France), Bruker Avance II 250.13 (^13^C, 63 MHz), Bruker Avance 400.13 (^13^C, 101 MHz) (Bruker, Wissembourg, France), or on a Bruker Avance III HD nanobay 400.13 (^13^C, 101 MHz) (Bruker, Wissembourg, France). Chemical shifts are expressed in parts per million (ppm) and were calibrated on deuterated or residual non-deuterated solvent peaks for ^1^H and ^13^C spectra. The following abbreviations are used for proton spectra multiplicities: b: broad, s: singlet, d: doublet, t: triplet, q: quartet, p: pentuplet, m: multiplet. Microwave-assisted reactions were carried out in a Biotage Initiator microwave synthesis instrument and temperatures were measured using an IR sensor (Biotage, Uppsala, Sweden). Melting points (p.m. (°C)) were taken on samples placed in open capillary tubes on a Thermo Fisher Melting Point Instrument Digital 9000 Series IA9200X6 and were not corrected. High-resolution mass spectra (HRMS) were performed on a Bruker 4G Maxis UHR-q-TOF mass spectrometer (Bruker, Wissembourg, France), with an electrospray ionization (ESI) mode. The numbering of the atoms on the molecules has been chosen arbitrarily and is indicated on the drawings of the molecules for a better understanding of the NMR spectra.

### 3.2. General Procedure (1)

The substituted 3-amino-2-chloropyridine **2** (1.65 mmol; 1.1 equiv.) and substituted isothiocyanate **3** (1.5 mmol; 1 equiv.) were dissolved in 1.0 mL of sabinene and stirred at 100 °C for 16 h. The mixture was allowed to cool to room temperature. The mixture was then filtered and washed with ethyl acetate followed by diethyl ether. The product was isolated without further purification.

### 3.3. General Procedure (2)

In a sealed tube, the substituted 3-amino-2-chloropyridine **2** (1.65 mmol ; 1.1 equiv.) and substituted isothiocyanate **3** (1.5 mmol ; 1 equiv.) were dissolved in 1.0 mL of sabinene. The mixture was placed under microwave irradiation for 2 h at 130 °C. The mixture was allowed to cool to room temperature. Then, the reaction was filtered and washed with ethyl acetate followed by diethyl ether. The product was isolated without further purification.

### 3.4. General Procedure (3)

In a sealed tube, the substituted 3-amino-2-chloropyridine **2** (1.65 mmol; 1.1 equiv.) and substituted isothiocyanate **3** (1.5 mmol; 1 equiv.) were dissolved in the solvent consisting of 0.75 mL sabinene and 0.25 mL acetonitrile. The mixture was placed under microwave irradiation for 2 h at 130 °C. After cooling to room temperature, the reaction mixture was filtered and washed with ethyl acetate followed by diethyl ether. The product was isolated without further purification.

*N*-phenylthiazolo[5,4-*b*]pyridin-2-amine hydrochloride (**4a**).

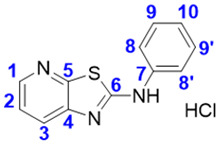



Using general procedure (1) applied to phenyl isothiocyanate **3a** and 3-amino-2-chloropyridine **2a**. Yield: 65%. Beige solid, m.p. 273 °C. (Lit. 284–285 °C) [41]. ^1^H NMR (400 MHz, DMSO-d6) δ 7.07 (tt, J = 7.3, 1.2 Hz, 1H, ^10^H_Ar_), 7.32–7.41 (m, 2H, ^9^H_Ar_ and ^9′^H_Ar_), 7.42 (dd, J = 8.1, 5.0 Hz, 1H, ^2^H_Ar_), 7.81 (dd, J = 7.5, 1.3 Hz, 2H, 8H_Ar_ and ^8′^H_Ar_), 7.97 (dd, J = 8.2, 1.5 Hz, 1H, ^3^H_Ar_), 8.29 (dd, J = 5.0, 1.5 Hz, 1H, ^1^H_Ar_), 10.94 (bs, 1H, N-H). ^13^C NMR (101 MHz, DMSO-d6) δ 118.4 (^8^CH_Ar_ and ^8′^CH_Ar_), 121.7 (^2^CH_Ar_), 122.8 (^10^CH_Ar_), 126.2 (^3^CH_Ar_), 129.0 (^9^CH_Ar_ and ^9′^CH_Ar_), 140.0 (^7^C^IV^), 141.8 (^1^CH_Ar_), 146.5 (^4^C^IV^), 153.2 (^5^C^IV^) and 161.1 (^6^C^IV^). HRMS (*m*/*z*) (ESI+): calcd. for *m*/*z* C_12_H_10_N_3_S [M + H^+^] = 228.0590; found = 228.0588.

*N*-(4-chlorophenyl)thiazolo[5,4-*b*]pyridin-2-amine hydrochloride (**4b**).

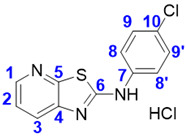



Using general procedure (1) applied to 4-chlorophenyl isothiocyanate and 3-amino-2-chloropyridine **2a**. Yield: 59%. Beige solid, m.p. 258 °C. ^1^H NMR (DMSO-d6, 400 MHz): δH = 7.39–7.46 (m, 3H, ^2^H_Ar_ + ^8^H_Ar_ + ^8′^H_Ar_), 7.86 (d, J = 8.0 Hz, 2H, ^9^H_Ar_ and ^9′^H_Ar_), 7.98 (d, J = 8.2 Hz, 1H, ^3^H_Ar_), 8.31 (d, J = 5.2 Hz, 1H, ^1^H_Ar_), 11.23 (bs, 1H, N-H). ^13^C NMR (DMSO-*d6*, 101 MHz): δ 119.8 (^9^CH_Ar_ and ^9′^CH_Ar_), 121.8 (^2^CH_Ar_), 126.2 (^10^C^IV^), 126.3 (^3^CH_Ar_), 128.9 (^8^CH_Ar_ and ^8′^CH_Ar_), 139.0 (^7^C^IV^), 142.2 (^1^CH_Ar_), 146.3 (^4^C^IV^), 153.4 (^5^C^IV^) and 160.8 (^6^C^IV^). HRMS (*m*/*z*) (ESI+): calcd. for *m*/*z* C_12_H_9_ClN_3_S [M + H^+^] = 262.0200; found = 262.0198.

*N*-(3,5-bis(trifluoromethyl)phenyl)thiazolo[5,4-*b*]pyridin-2-amine hydrochloride (**4c**).

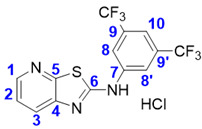



Using general procedure (3) applied to 3,5-Bis(trifluoromethyl)phenyl isothiocyanate and 3-amino-2-chloropyridine **2a**. Yield: 54%. Colourless solid, m.p. 231 °C. ^1^H NMR (DMSO-*d6*, 400 MHz): δ 7.43 (dd, J = 8.2, 4.9 Hz, 1H, ^2^H_Ar_), 7.69 (s, 1H, ^10^H_Ar_), 8.03 (d, J = 8.1 Hz, 1H, ^3^H_Ar_), 8.34 (d, J = 4.9 Hz, 1H, ^1^H_Ar_), 8.51 (s, 2H, ^8^H_Ar_ and ^8′^H_Ar_), 11.89 (bs, 1H, N-H). ^13^C NMR (DMSO-*d6*, 101 MHz): δ 114.8 (^10^CH_Ar_), 117.6 (^8^CH_Ar_ and ^8′^CH_Ar_), 121.8 (^2^CH_Ar_), 123.3 (q, ^1^J = 274 Hz, ^11^CF_3_ and ^11′^CF_3_), 126.7 (^3^CH_Ar_), 130.9 (q, ^2^J = 32 Hz, ^9^C^IV^ and ^9′^C^IV^), 141.8 (^7^C^IV^), 143.6 (^1^CH_Ar_), 145.3 (^4^C^IV^), 153.8 (^5^C^IV^) and 160.4 (^6^C^IV^). ^19^F NMR (DMSO-*d6*, 376 MHz): δ 61.66. HRMS (*m*/*z*) (ESI+): calcd. for *m*/*z* C_14_H_8_F_6_N_3_S [M + H^+^] = 364.0338; found = 364.0341.

*N*-(4-methoxyphenyl)thiazolo[5,4-*b*]pyridin-2-amine hydrochloride (**4d**).

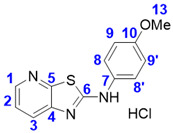



Using general procedure (1) applied to 4-methoxyphenyl isothiocyanate and 3-amino-2-chloropyridine **2a**. Yield: 54%. Yellow solid, m.p. 241 °C. ^1^H NMR (DMSO-*d6*, 400 MHz): δ 3.74 (s, 3H, ^11^CH_3_-O), 6.96 (d, J = 7.0 Hz, 2H, ^8^CH_Ar_ and ^8′^CH_Ar_), 7.41 (dd, J = 8.1, 5.0 Hz, 1H, ^2^CH_Ar_), 7.69 (d, J = 7.0 Hz, 2H, ^9^CH_Ar_ and ^9′^CH_Ar_), 7.93 (d, J = 8.1 Hz, 1H, ^3^CH_Ar_), 8.27 (d, J = 5.1 Hz, 1H, ^1^CH_Ar_), 10.93 (bs, 1H, N-H). ^13^C NMR (DMSO-*d6*, 101 MHz): δ 55.3 (^11^CH_3_-O), 114.3 (^8^CH_Ar_ and ^8′^CH_Ar_), 120.5 (^9^CH_Ar_ and ^9′^CH_Ar_), 121.8 (^2^CH_Ar_), 125.8 (^3^CH_Ar_), 133.1 (^7^C^IV^), 141.1 (^1^CH_Ar_), 146.7 (^4^C^IV^), 152.8 (^5^C^IV^), 155.3 (^10^C^IV^) and 161.6 (^6^C^IV^). HRMS (*m*/*z*) (ESI+): calcd. for *m*/*z* C_13_H_12_N_3_OS [M + H^+^] = 258.0695; found = 258.0693.

*N*-(4-bromophenyl)thiazolo[5,4-*b*]pyridin-2-amine hydrochloride (**4e**).

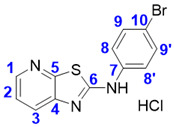



Using general procedure (1) applied to 4-bromophenyl isothiocyanate and 3-amino-2-chloropyridine **2a**. Yield: 66%. Beige solid, m.p. 264 °C (decomposition). ^1^H NMR (DMSO-*d6*, 400 MHz): δ 7.41 (dd, J = 8.1, 4.9 Hz, 1H, ^2^H_Ar_), 7.55 (d, J = 8.8 Hz, 2H, ^8^H_Ar_ and ^8′^H_Ar_), 7.80 (d, J = 8.9 Hz, 2H, ^9^H_Ar_ and ^9′^H_Ar_), 7.97 (dd, J = 8.2, 1.6 Hz, 1H, ^3^H_Ar_), 8.30 (dd, J = 4.9, 1.6 Hz, 1H, ^1^H_Ar_), 11.16 (bs, 1H, N-H). ^13^C NMR (DMSO-*d6*, 101 MHz): δ 114.1 (^10^C^IV^), 120.2 (^9^CH_Ar_ and ^9′^CH_Ar_), 121.7 (^2^CH_Ar_), 126.1 (^3^CH_Ar_), 131.8 (^8^CH_Ar_ and ^8′^CH_Ar_), 139.4 (^7^C^IV^), 142.5 (^1^CH_Ar_), 146.1 (^4^C^IV^), 153.6 (^5^C^IV^) and 160.6 (^6^C^IV^). HRMS (*m*/*z*) (ESI+): calcd. for *m*/*z* C_12_H_9_BrN_3_S [M + H^+^] = 305.9695; found = 305.9698.

*N*-(3-bromophenyl)thiazolo[5,4-*b*]pyridin-2-amine hydrochloride (**4f**).

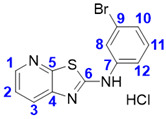



Using general procedure (1) applied to 3-bromophenyl isothiocyanate and 3-amino-2-chloropyridine **2a**. Yield: 58%. Yellowish solid, m.p. 231 °C. ^1^H NMR (DMSO-*d6*, 400 MHz): δ 7.23 (d, J = 8.1 Hz, 1H, ^12^H_Ar_), 7.33 (t, J = 8.1 Hz, 1H, ^11^H_Ar_), 7.41 (dd, J = 8.4, 5.2 Hz, 1H, ^2^H_Ar_), 7.71 (d, J = 8.2 Hz, 1H, ^10^H_Ar_), 7.99 (d, J = 8.1 Hz, 1H, ^3^H_Ar_), 8.18 (s, 1H, ^8^H_Ar_), 8.29 (d, J = 5.3 Hz, 1H, ^1^H_Ar_), 11.12 (bs, 1H, N-H). ^13^C NMR (DMSO-*d6*, 101 MHz): δ 117.2 (^10^CH_Ar_), 120.5 (^8^CH_Ar_), 121.8 (^2^CH_Ar_), 121.9 (^9^C^IV^), 125.2 (^12^CH_Ar_), 127.0 (^3^CH_Ar_), 130.9 (^11^CH_Ar_), 141.5 (^1^CH_Ar_), 141.6 (^7^C^IV^), 146.5 (^4^C^IV^), 152.7 (^5^C^IV^) and 160.8 (^6^C^IV^). HRMS (*m*/*z*) (ESI+): calcd. for *m*/*z* C_12_H_9_BrN_3_S [M + H^+^] = 305.9695; found = 305.9689.

*N*-(3-chlorophenyl)thiazolo[5,4-*b*]pyridin-2-amine hydrochloride (**4g**).

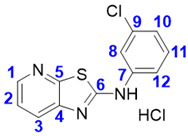



Using general procedure (1) applied to 3-chlorophenyl isothiocyanate and 3-amino-2-chloropyridine **2a**. Yield: 63%. Beige solid, m.p. 205 °C. ^1^H NMR (DMSO-*d6*, 400 MHz): δ 7.10 (dd, J = 7.8, 2.4 Hz, 1H, ^12^H_Ar_), 7.39 (t, J = 8.2 Hz, 1H, ^11^H_Ar_), 7.44 (dd, J = 8.0, 4.9 Hz, 1H, ^2^H_Ar_), 7.67 (dd, J = 8.2, 2.6 Hz, 1H, ^10^H_Ar_), 8.03 (dd, J = 8.2, 1.6 Hz, 1H, ^3^H_Ar_), 8.07 (m, 1H, ^8^H_Ar_), 8.32 (dd, J = 4.9, 1.6 Hz, 1H, ^1^H_Ar_), 11.32 (bs, 1H, N-H). ^13^C NMR (DMSO-*d6*, 101 MHz): δ 116.7 (^10^CH_Ar_), 117.6 (^8^CH_Ar_), 121.8 (^2^CH_Ar_), 122.3 (^12^CH_Ar_), 126.5 (^3^CH_Ar_), 130.6 (^11^CH_Ar_), 133.3 (^9^C^IV^), 141.4 (^7^C^IV^), 142.4 (^1^CH_Ar_), 146.1 (^4^C^IV^), 153.4 (^5^C^IV^) and 160.7 (^6^C^IV^). HRMS (*m*/*z*) (ESI+): calcd. for *m*/*z* C_12_H_9_ClN_3_S [M + H^+^] = 262.0200; found = 262.0202.

*N*-(3,5-dichlorophenyl)thiazolo[5,4-*b*]pyridin-2-amine hydrochloride (**4h**).

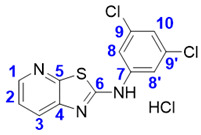



Using general procedure (3) applied to 3,5-dichlorophenyl isothiocyanate and 3-amino-2-chloropyridine **2a**. Yield: 66%. Beige solid, m.p. 265 °C (decomposition). ^1^H NMR (DMSO-*d6*, 400 MHz): δ 7.22 (t, J = 1.8 Hz, 1H, ^10^H_Ar_), 7.43 (dd, J = 8.2, 4.9 Hz, 1H, ^2^H_Ar_), 7.91 (d, J = 1.8 Hz, 2H, ^8^H_Ar_ and ^8′^H_Ar_), 8.05 (dd, J = 8.2, 1.6 Hz, 1H, ^3^H_Ar_), 8.33 (dd, J = 4.9, 1.6 Hz, 1H, ^1^H_Ar_), 11.54 (bs, 1H, N-H). ^13^C NMR (DMSO-*d6*, 101 MHz): δ 116.2 (^8^CH_Ar_ and ^8′^CH_Ar_), 121.5 (^10^CH_Ar_), 121.8 (^2^CH_Ar_), 126.6 (^3^CH_Ar_), 134.3 (^9^C^IV^ and ^9′^C^IV^), 142.2 (^7^C^IV^), 143.2 (^1^CH_Ar_), 145.6 (^4^C^IV^), 153.7 (^5^C^IV^) and 160.3 (^6^C^IV^). HRMS (*m*/*z*) (ESI+): calcd. for *m*/*z* C_12_H_8_Cl_2_N_3_S [M + H^+^] = 295.9811; found = 295.9810.

*N*-(ethyl 4-aminobenzoate)thiazolo[5,4-*b*]pyridin-2-amine hydrochloride (**4i**).

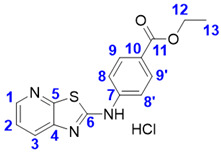



Using general procedure (3) applied to ethyl 4-isothiocyanatobenzoate and 3-amino-2-chloropyridine **2a**. Yield: 55%. Beige solid, m.p. 230 °C. ^1^H NMR (DMSO-*d6*, 400 MHz): δ 1.31 (t, J = 7.1 Hz, 3H, ^13^CH_3_), 4.28 (q, J = 7.1 Hz, 2H, ^12^CH_2_), 7.45 (dd, J = 8.1, 4.9 Hz, 1H, ^2^H_Ar_), 7.96 (s, 4H, H_Ar_), 8.04 (d, J = 8.3 Hz, 1H, ^3^H_Ar_), 8.34 (d, J = 4.9 Hz, 1H, ^1^H_Ar_), 11.48 (bs, 1H, N-H). ^13^C NMR (DMSO-*d6*, 101 MHz): δ 14.3 (^13^CH_3_), 60.4 (^12^CH_2_), 117.6 (2xCH_Ar_), 121.8 (^2^CH), 123.5 (^10^C^IV^), 126.8 (^3^CH), 130.5 (2xCH_Ar_), 142.6 (^1^CH), 144.2 (^7^C^IV^), 146.1 (^4^C^IV^), 153.5 (^5^C^IV^), 160.5 (^6^C^IV^) and 165.3 (^11^C^IV^=O). HRMS (*m*/*z*) (ESI+): calcd. for *m*/*z* C_15_H_14_N_3_O_2_S [M + H^+^] = 300.0801; found = 300.0801.

*N*-methylthiazolo[5,4-*b*]pyridin-2-amine hydrochloride (**4j**).

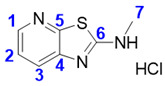



Using general procedure (1) applied to methyl isothiocyanate and 3-amino-2-chloropyridine **2a**. Yield: 50%. Beige solid, m.p. 239 °C (lit. 264–265) [41]. ^1^H NMR (DMSO-*d6*, 400 MHz): δ 3.06 (s, 3H, ^7^CH_3_), 7.43 (dd, J = 8.1, 5.0 Hz, 1H, ^2^H_Ar_), 7.88 (d, J = 8.1 Hz, ^3^H_Ar_), 8.27 (d, J = 5.0 Hz, 1H, ^1^H_Ar_), 9.69 (bs, 1H, N-H). ^13^C NMR (DMSO-*d6*, 101 MHz): δ 30.9 (^7^CH_3_), 122.0 (^2^CH_Ar_), 123.7 (^3^CH_Ar_), 141.6 (^1^CH_Ar_), 142.4 (^4^C^IV^), 150.6 (^5^C^IV^) and 165.9 (^6^C^IV^). HRMS (*m*/*z*) (ESI+): calcd. for *m*/*z* C_7_H_8_N_3_S [M + H^+^] = 166.0433; found = 166.0438.

*N*-benzamidethiazolo[5,4-*b*]pyridin-2-amine hydrochloride (**4k**).

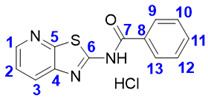



Using general procedure (1) applied to benzoyl isothiocyanate and 3-amino-2-chloropyridine **2a**. Yield: 46%. Beige solid, m.p. 183 °C. ^1^H NMR (DMSO-*d6*, 400 MHz): δ 7.54 (dd, J = 8.2, 4.6 Hz, 1H, ^2^H_Ar_), 7.58 (t, J = 7.6 Hz, 2H, ^10^H_Ar_ and ^10′^H_Ar_), 7.67–7.71 (m, 1H, H_Ar_), 8.11–8.20 (m, 3H, ^9^H_Ar_ + ^9′^H_Ar_ + ^3^H_Ar_), 8.52 (dd, J = 4.8, 1.4 Hz, 1.0H, ^1^H_Ar_), 12.95 (bs, 1H, N-H). ^13^C NMR (DMSO-*d6*, 101 MHz): δ 121.8 (^2^CH_Ar_), 127.6 (^3^CH_Ar_), 128.4 (^9^CH_Ar_ and ^9′^CH_Ar_), 128.7 (^10^CH_Ar_ and ^10′^CH_Ar_), 131.6 (^8^C^IV^), 133.1 (^11^CH_Ar_), 141.8 (^4^C^IV^), 145.4 (^1^CH_Ar_), 154.7 (^5^C^IV^), 158.4 (^6^C^IV^) and 166.3 (^7^C^IV^=O). HRMS (*m*/*z*) (ESI+): calcd. for *m*/*z* C_13_H_10_N_3_OS [M + H^+^] = 256.0539; found = 256.0541.

*N*-phenylthiazolo[5,4-*b*]-6-methylpyridin-2-amine hydrochloride (**5a**).

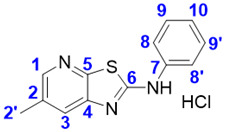



Using general procedure (2) applied to phenyl isothiocyanate **3a** and 3-amino-2-chloro-5-methylpyridine **2b**. Yield: 64%. Beige solid, m.p. 228 °C (decomposition). ^1^H NMR (DMSO-*d6*, 400 MHz): δ 2.38 (s, 3H, ^2′^CH_3_), 7.06 (t, J = 7.4 Hz, 1H, ^10^H_Ar_), 7.38 (t, J = 7.6 Hz, 2H, ^9^H_Ar_ and ^9′^H_Ar_), 7.80 (d, J = 8.3 Hz, 2H, ^8^H_Ar_ and ^8′^H_Ar_), 7.88 (s, 1H, ^3^H_Ar_), 8.20 (s, 1H, ^1^H_Ar_), 10.93 (bs, 1H, N-H). ^13^C NMR (DMSO-*d6*, 101 MHz): δ 17.8 (^2′^CH_3_), 118.5 (^8^CH_Ar_ and ^8′^CH_Ar_), 122.9 (^10^CH_Ar_), 127.4 (^3^CH_Ar_), 129.1 (^9^CH_Ar_ and ^9′^CH_Ar_), 131.7 (^2^C^IV^), 140.0 (^7^C^IV^), 141.2 (^1^CH_Ar_), 146.9 (^4^C^IV^), 149.4 (^5^C^IV^) and 161.5 (^6^C^IV^). HRMS (*m*/*z*) (ESI+): calcd. for *m*/*z* C_13_H_12_N_3_S [M + H^+^] = 242.0746; found = 242.0749.

*N*-(3-bromophenyl)thiazolo[5,4-*b*]-6-methylpyridin-2-amine hydrochloride (**5b**).

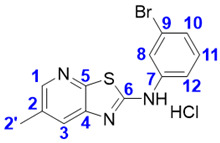



Using general procedure (1) applied to 3-bromophenyl isothiocyanate and 3-amino-2-chloro-5-methylpyridine **2b**. Yield: 62%. Beige solid, m.p. 247–248 °C. ^1^H NMR (DMSO-*d6*, 400 MHz): δ 2.38 (s, 3H, ^2′^CH_3_), 7.23 (d, J = 7.8 Hz, 1H, ^12^H_Ar_), 7.32 (t, J = 7.9 Hz, 1H, ^11^H_Ar_), 7.69 (d, J = 8.1 Hz, 1H, ^10^H_Ar_), 7.91 (s, 1H, ^3^H_Ar_), 8.20 (s, 2H, ^1^H_Ar_ and ^8^H_Ar_), 11.26 (bs, 1H, N-H). ^13^C NMR (DMSO-*d6*, 101 MHz): δ 17.8 (^2′^CH_3_), 117.0 (^10^CH_Ar_), 120.4 (^8^CH_Ar_), 121.8 (^9^C^IV^), 125.1 (^12^CH_Ar_), 127.3 (^3^CH_Ar_), 130.9 (^11^CH_Ar_), 131.6 (^2^C^IV^), 141.5 (^7^C^IV^), 142.5 (^1^CH_Ar_), 146.2 (^4^C^IV^), 150.1 (^5^C^IV^) and 160.9 (^6^C^IV^). HRMS (*m*/*z*) (ESI+): calcd. for *m*/*z* C_13_H_11_BrN_3_S [M + H^+^] = 319.9852; found = 319.9847.

*N*-(3,5-bis(trifluoromethyl)phenyl)thiazolo[5,4-*b*]-6-methylpyridin-2-amine hydrochloride (**5c**).

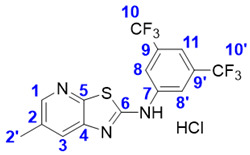



Using general procedure (2) applied to 3,5-Bis(trifluoromethyl)phenyl isothiocyanate and 3-amino-2-chloro-5-methylpyridine **2b**. Yield: 48%. Beige solid, m.p. 242 °C (decomposition). ^1^H NMR (DMSO-*d6*, 400 MHz): δ 2.37 (s, 3H, ^2′^CH_3_), 7.67 (s, 1H, ^11^H_Ar_), 7.89 (s, 1H, ^3^H_Ar_), 8.19 (s, 1H, ^1^H_Ar_), 8.49 (s, 2H, ^8^CH_Ar_ and ^8′^CH_Ar_), 11.83 (bs, 1H, N-H). ^13^C NMR (DMSO-*d6*, 101 MHz): δ 17.7 (^2′^CH_3_), 114.8 (^11^CH_Ar_), 117.6 (^8^CH_Ar_ and ^8′^CH_Ar_), 123.3 (q, ^1^J = 274 Hz, ^10^CF_3_ and ^10′^CF_3_), 127.3 (^3^CH_Ar_), 130.9 (q, ^2^J = 33 Hz, ^9^C^IV^ and ^9′^C^IV^), 131.6 (^2^C^IV^), 141.8 (^7^C^IV^), 143.9 (^1^CH_Ar_), 145.3 (^4^C^IV^), 150.6 (^5^C^IV^) and 160.6 (^6^C^IV^). ^19^F NMR (DMSO-*d6*, 376 MHz):δ -61.68. HRMS (*m*/*z*) (ESI+): calcd. for *m*/*z* C_15_H_10_F_6_N_3_S [M + H^+^] = 378.0494; found = 378.0491.

*N*-(4-methoxyphenyl)thiazolo[5,4-*b*]-6-methylpyridin-2-amine hydrochloride (**5d**).

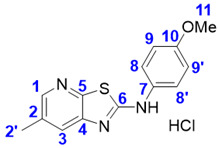



Using general procedure (2) applied to 4-methoxyphenyl isothiocyanate and 3-amino-2-chloro-5-methylpyridine **2b**. Yield: 42%. Yellow solid, m.p. 212 °C (decomposition). ^1^H NMR (DMSO-*d6*, 400 MHz): δ 2.37 (s, 3H, ^2′^CH_3_), 3.75 (s, 3H, ^11^CH_3_-O), 6.96 (d, J = 9.0 Hz, 2H, ^8^H_Ar_ and ^8′^H_Ar_), 7.67 (d, J = 9.0 Hz, 2H, ^9^H_Ar_ and ^9′^H_Ar_), 7.84 (s, 1H, ^3^H_Ar_), 8.17 (s, 1H, ^1^H_Ar_), 10.80 (bs, 1H, N-H). ^13^C NMR (DMSO-*d6*, 101 MHz): δ 17.8 (^2′^CH_3_), 55.3 (^11^CH_3_-O), 114.3 (^8^CH_Ar_ and ^8′^CH_Ar_), 120.6 (^9^CH_Ar_ and ^9′^CH_Ar_), 127.0 (^3^CH_Ar_), 131.8 (^2^C^IV^), 133.1 (^7^C^IV^), 140.5 (^1^CH_Ar_), 147.0 (^4^C^IV^), 149.1 (^5^C^IV^), 155.4 (^10^C^IV^) and 162.1 (^6^C^IV^). HRMS (*m*/*z*) (ESI+): calcd. for *m*/*z* C_14_H_14_N_3_OS [M + H^+^] = 272.0852; found = 272.0856.

*N*-(3,5-dichlorophenyl)thiazolo[5,4-*b*]-6-methylpyridin-2-amine hydrochloride (**5e**).

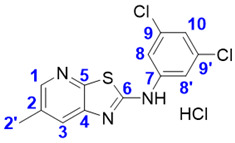



Using general procedure (2) applied to 3,5-dichlorophenyl isothiocyanate and 3-amino-2-chloro-5-methylpyridine **2b**. Yield: 59%. Pinkish solid, m.p. 251 °C. ^1^H NMR (DMSO-*d6*, 250 MHz): δ 2.37 (s, 3H, ^2′^CH_3_), 7.21 (t, J = 1.9 Hz, 1H, ^10^H_Ar_), 7.89 (d, J = 1.9 Hz, 2H, ^8^H_Ar_ and ^8′^H_Ar_), 7.93 (d, J = 1.0 Hz, 1H, ^3^H_Ar_), 8.20 (d, J = 1.2 Hz, 1H, ^1^H_Ar_), 11.51 (bs, 1H, N-H). ^13^C NMR (DMSO-*d6*, 101 MHz): δ 17.7 (^2′^CH_3_), 116.1 (^8^CH_Ar_ and ^8′^CH_Ar_), 121.4 (^10^CH_Ar_), 127.4 (^3^CH_Ar_), 131.6 (^2^C^IV^), 134.2 (^9^C^IV^ and ^9′^C^IV^), 142.2 (^7^C^IV^), 143.2 (^1^CH_Ar_), 145.7 (^4^C^IV^), 150.3 (^5^C^IV^) and 160.6 (^6^C^IV^). HRMS (*m*/*z*) (ESI+): calcd. for *m*/*z* C_13_H_10_Cl_2_N_3_S [M + H^+^] = 309.9967; found = 309.9973.

*N*-(3-chlorophenyl)thiazolo[5,4-*b*]-6-methylpyridin-2-amine hydrochloride (**5f**).

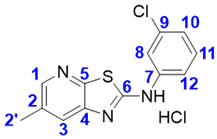



Using general procedure (1) applied to 3-chlorophenyl isothiocyanate and 3-amino-2-chloro-5-methylpyridine **2b**. Yield: 67%. Colourless solid, m.p. 214 °C. ^1^H NMR (DMSO-*d6*, 400 MHz): δ 2.38 (s, 3H, ^2′^CH_3_), 7.09 (dd, J = 8.0, 2.3 Hz, 1H, ^10^H_Ar_), 7.38 (t, J = 8.0 Hz, 1H, ^11^H_Ar_), 7.64 (dd, J = 8.2, 2.3 Hz, 1H, ^12^H_Ar_), 7.93 (s, 1H, ^3^H_Ar_), 8.08 (s, 1H, ^8^H_Ar_), 8.21 (s, 1H, ^1^H_Ar_), 11.34 (bs, 1H, N-H). ^13^C NMR (DMSO-*d6*, 101 MHz): δ 17.8 (^2′^CH_3_), 116.7 (^12^CH_Ar_), 117.6 (^8^CH_Ar_), 122.2 (^10^CH_Ar_), 127.6 (^3^CH_Ar_), 130.6 (^11^CH_Ar_), 131.7 (^2^C^IV^), 133.3 (^9^C^IV^), 141.4 (^7^C^IV^), 141.9 (^1^CH_Ar_), 146.4 (^4^C^IV^), 149.7 (^5^C^IV^) and 161.0 (^6^C^IV^). HRMS (*m*/*z*) (ESI+): calcd. for *m*/*z* C_13_H_11_ClN_3_S [M + H^+^] = 276.0357; found = 267.0360.

*N*-benzamidethiazolo[5,4-*b*]-6-methyl-2-amine hydrochloride (**5g**).

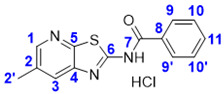



Using general procedure (1) applied to benzoyl isothiocyanate and 3-amino-2-chloro-5-methylpyridine **2b**. Yield: 46%. Colourless solid, m.p. 222 °C. ^1^H NMR (DMSO-*d6*, 400 MHz): δ 2.43 (s, 3H, ^2′^CH_3_), 7.57 (t, J = 7.7 Hz, 2H, ^10^H_Ar_ and ^10′^H_Ar_), 7.67 (t, J = 7.3 Hz, 1H, ^11^H_Ar_), 7.97 (s, 1H, ^3^H_Ar_), 8.13 (d, J = 7.8 Hz, 2H, ^9^H_Ar_ and ^9′^H_Ar_), 8.35 (s, 1H, ^1^H_Ar_), 12.91 (bs, 1H, N-H). ^13^C NMR (DMSO-*d6*, 101 MHz): δ 17.9 (^2′^CH_3_), 127.7 (^3^CH_Ar_), 128.4 (^9^CH_Ar_ and ^9′^CH_Ar_), 128.7 (^10^CH_Ar_ and ^10′^CH_Ar_), 131.5 (^2^C^IV^), 131.7 (^8^C^IV^), 133.1 (^11^CH_Ar_), 141.7 (^4^C^IV^), 146.2 (^1^CH_Ar_), 151.8 (^5^C^IV^), 158.6 (^6^C^IV^) and 166.2 (^7^C^IV^=O). HRMS (*m*/*z*) (ESI+): calcd. for *m*/*z* C_14_H_12_N_3_OS [M + H^+^] = 270.0696; found = 270.0698.

*N*-(ethyl 4-aminobenzoate)thiazolo[5,4-*b*]-6-methylpyridin-2-amine hydrochloride (**5h**).

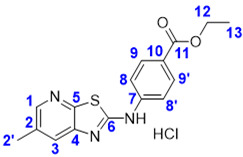



Using general procedure (1) applied to ethyl 4-isothiocyanatobenzoate and 3-amino-2-chloro-5-methylpyridine **2b**. Yield: 64%. Beige solid, m.p. 210 °C (decomposition). ^1^H NMR (DMSO-*d6*, 400 MHz): δ 1.31 (t, J = 7.0 Hz, 3H, ^13^CH_3_), 2.38 (s, 3H, ^2′^CH_3_), 4.28 (q, J = 7.0 Hz, 2H, ^12^CH_2_), 7.89 (s, 1H, ^3^H_Ar_), 7.89–7.98 (m, 4H, H_Ar_), 8.20 (s, 1H, ^1^H_Ar_), 11.41 (bs, 1H, N-H). ^13^C NMR (DMSO-*d6*, 101 MHz): δ 14.2 (^13^CH_3_), 17.8 (^2′^CH_3_), 60.4 (^12^CH_2_), 117.5 (2 x CH_Ar_), 123.4 (^10^C^IV^), 127.4 (^3^CH_Ar_), 130.5 (2 x CH_Ar_), 131.6 (^2^C^IV^), 142.7 (^1^CH), 144.2 (^7^C^IV^), 146.1 (^4^C^IV^), 150.2 (^5^C^IV^), 160.8 (^6^C^IV^) and 165.3 (^11^C^IV^). HRMS (*m*/*z*) (ESI+): calcd. for *m*/*z* C_16_H_16_N_3_O_2_S [M + H^+^] = 314.0958; found = 314.0956.

*N*-methylthiazolo[5,4-*b*]-6-methylpyridin-2-amine hydrochloride (**5i**).

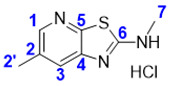



Using general procedure (1) applied to methyl isothiocyanate and 3-amino-2-chloro-5-methylpyridine **2b**. Yield: 40%. Colourless solid, m.p. 186 °C (decomposition). ^1^H NMR (D_2_O, 400 MHz): δ 2.39 (s, 3H, ^2′^CH_3_), 3.13 (s, 3H, ^7^CH_3_), 7.60 (s, 1H, ^3^H_Ar_), 8.12 (s, 1H, ^1^H_Ar_). ^13^C NMR (D_2_O, 101 MHz): δ 17.5 (^2′^CH_3_), 31.4 (^7^CH_3_), 124.3 (^3^CH_Ar_), 134.2 (^2^C^IV^), 138.1 (C^IV^), 142.8 (^1^CH_Ar_), 143.3 (C^IV^) and 167.7 (^6^C^IV^). HRMS (*m*/*z*) (ESI+): calcd. for *m*/*z* C_8_H_10_N_3_S [M + H^+^] = 180.0590; found = 180.0587.

## 4. Conclusions

Following our work on eucalyptol as a new green solvent, we show in this article that sabinene is also potentially usable as another new biomass-derived green solvent.

## Figures and Tables

**Table 1:** Optimisation time of reaction in various solvents.

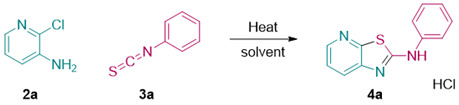				
Entry	Solvent	T (°C)	Time (h)	Yield (%)
1	Acetone	100	4	60
2	DCM	100	4	43
3	Toluene	100	4	44
4	Dioxane	100	4	44
5	THF	100	4	43
6	Eucalyptol	100	4	59
7	Eucalyptol	100	16	75
8	CPME	100	4	63
9	CPME	100	16	71
10	Sabinene	100	4	36
11	Sabinene	100	16	58
12	Limonene	100	4	65
13	Limonene	100	16	70
14	Citral	100	16	21

**Table 2:** Optimising reaction time and reagent equivalence in green solvents.

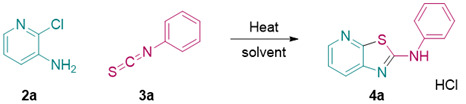						
Entry	2-chloro-3-amino-pyridine (Equiv.)	Isothiocyanate (Equiv.)	Reaction Time (h)	TP (°C)	Solvent	Yields
1	1	1	4	100	Eucalyptol	59%
2	1	1	16	100	Eucalyptol	75%
3	1	1	4	100	CPME	63%
4	1	1	16	100	CPME	71%
5	1.1	1	16	100	CPME	79%
6	1	1	4	100	Limonene	65%
7	1	1	16	100	Limonene	70%
8	1	1	4	100	Sabinene	36%
9	1.1	1	4	100	Sabinene	38%
10	1	1.1	4	100	Sabinene	33%
11	1	1	16	100	Sabinene	68%
12	1.1	1	16	100	Sabinene	76%
13	1.1	1	16	100	Distilled Sabinene	62%
14	1	1	16	100	Distilled Sabinene	58%

**Table 3:** Optimisation of the reaction conditions under microwaves.

Entry	Reaction Time	Temperature (°C)	Yield (%)	Solvant
1	1 h	160	67	Sabinene
2	2 h	150	59	
3	2 h	130	55	
4	4 h	130	62	
5	2 h	130	44	75:25 Sabinene/Ethanol
6	2 h	130	64	75:25 Sabinene/ACN

**Table 4:** Optimisation from 3-amino-2-chloro-5-methylpyridine **2b** under thermal conditions.

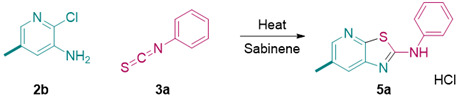						
Entry	Reaction Time	Pyridine (Equiv.)	Isothiocyanate (Equiv.)	Rinsing Solvent	Yield	NMR Observation
1	5 h	1	1	Diethyl ether	46%	Clear
2	5 h	1.1	1	Diethyl ether	55%	Parasite peak
3	5 h	1	1.1	Diethyl ether	53%	Parasite peak
4	16 h	1.1	1	Diethyl ether	75%	Parasite peak
5	16 h	1.1	1	Ethyl acetate	66%	Clear

**Table 5:** Optimisation under microwave irradiation.

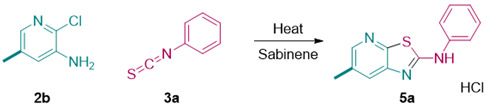			
Entry	Reaction Time	Temperature	Yield
1	30 min	150 °C	40%
2	1 h	150 °C	61%
3	1 h	160 °C	57%
4	2 h	150 °C	63%
6	2 h	110 °C	37%
7	2 h	130 °C	64%
8	1 h	130 °C	50%

## Data Availability

All data are reported in the manuscript and Supplementary Materials.

## Supplementary Materials

The following supporting information can be downloaded at: https://www.mdpi.com/article/10.3390/molecules28196924/s1, Characterization data for obtained products and copies of 1H, ^13^C NMR spectra and HRMS. Reference [41] is cited in the Supplementary Materials.

## Sample Availability

Samples of the compounds are available from the authors.
