# Supplementary material for: Sabinene: A New Green Solvent Used in the Synthesis of Thiazolo[5,4-b]pyridines by Thermal or Microwave Activation
Source: Molecules. 2023 Oct 3;28(19):6924. doi: 10.3390/molecules28196924 (PMC10574264; doi:10.3390/molecules28196924)
Supplement: Supplementary file 1 [file molecules-28-06924-s001.zip › molecules-2601986-supplementary.pdf]

molecules

Sabinene: A New Green Solvent Used in the Synthesis of Thiazolo[5,4-*b*]pyridines by Thermal or Microwave Activation

Gatien Messire, Véronique Ferreira, Emma Caillet, Lyana Bodin, Amélia Auville and Sabine Berteina-Raboin *

Institut de Chimie Organique et Analytique (ICOA), Université d'Orléans UMR-CNRS 7311, BP 6759, rue de Chartres, 45067 Orléans, France;
gatien.messire@univ-orleans.fr (G.M.); veronique.ferreira-bardin@univ-orleans.fr (V.F.);
emma.caillet@etu.univ-orleans.fr (E.C.); lyana.bodin@etu.umontpellier.fr (L.B.);
amelia.auville@etu.univ-orleans.fr (A.A.)
* Correspondence: sabine.berteina-raboin@univ-orleans.fr; Tel.: +33-238-494-856

Supporting Information

molecules

MDPI

Experimental part

All reagents were purchased from commercial suppliers and used without further purification. Sabinene was purchased from Merck with 75% of purity. Unless otherwise specifed, sabinene was used in its commercial form. ^{1}H and ^{13}C NMR spectra were recorded on a Bruker DPX 250 (^{13}C, 62.9 MHz) (Bruker, Wissembourg, France), Bruker Avance II 250.13 (^{13}C, 63 MHz), Bruker Avance 400.13 (^{13}C, 101 MHz) (Bruker, Wissembourg, France), or on a Bruker Avance III HD nanobay 400.13 (^{13}C, 101 MHz) (Bruker, Wissembourg, France). Chemical shifts are expressed in parts per million (ppm) and were calibrated on deuterated or residual non-deuterated solvent peaks for ^{1}H and ^{13}C spectra. The following abbreviations are used for proton spectra multiplicities: b: broad, s: singlet, d: doublet, t: triplet, q: quartet, p: pentuplet, m: multiplet. Microwave-assisted reactions were carried out in a Biotage Initiator microwave synthesis instrument and temperatures were measured by an IR sensor (Biotage, Uppsala, Sweden). Melting points (p.m. [°C]) were taken on samples placed in open capillary tubes on a Thermo Fisher Melting Point Instrument Digital 9000 Series IA9200X6 and were not corrected. High-resolution mass spectra (HRMS) were performed on a Bruker 4G Maxis UHR-q-TOF mass spectrometer (Bruker, Wissembourg, France), with an electrospray ionization (ESI) mode. The numbering of the atoms on the molecules has been chosen arbitrarily and is indicated on the drawings of the molecules for a better understanding of the NMR spectra.

General procedure (1) : Substituted 3-amino-2-chloropyridine **2** (1.65 mmol; 1.1 equiv.) and substituted isothiocyanate **3** (1.5 mmol; 1 equiv.) were dissolved in 1.0 mL of sabinene and stirred at 100°C for 16 h. The mixture was allowed to cool to room temperature. The mixture was then filtered and washed with ethyl acetate followed by diethyl ether. The product was isolated without further purification.

General procedure (2) : In a sealed tube, substituted 3-amino-2-chloropyridine **2** (1,65 mmol ; 1,1 equiv.) and substituted isothiocyanate **3** (1,5 mmol ; 1 equiv.) were dissolved in 1.0 mL of sabinene. The mixture was placed under microwave irradiation during 2 h at 130 °C. The mixture was allowed to cool to room temperature. Then, the reaction was filtered and washed with ethyl acetate followed by diethyl ether. The product was isolated without further purification.

General procedure (3) : In a sealed tube, substituted 3-amino-2-chloropyridine **2** (1.65 mmol; 1.1 equiv.) and substituted isothiocyanate **3** (1.5 mmol; 1 equiv.) were dissolved in the solvent consisting of 0.75 ml sabinene and 0.25 ml acetonitrile. The mixture was placed under microwave irradiation for 2 h at 130°C. After cooling to room temperature, the reaction mixture was filtered and washed with ethyl acetate followed by diethyl ether. The product was isolated without further purification.

molecules MDPI

N-phenylthiazolo[5,4-b]pyridin-2-amine hydrochloride (**4a**)

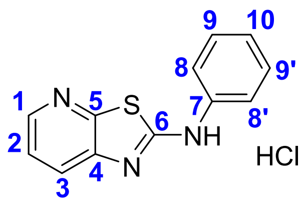

General procedure 1 was applied to phenyl isothiocyanate **3a** and 3-amino-2-chloropyridine **2a** to give *N*-phenylthiazolo[5,4-*b*]pyridin-2-amine hydrochloride **4a** in 65% yield.

Aspect : beige powder

Melting point : 273°C (lit. 284-285°C) [41]

HRMS (ESI+): m/z calculated for $C_{12}H_{10}N_3S$ [M + H^+] = 228.0590 ; found = 228.0588

^{1}H NMR (DMSO-*d6*, 400 MHz) : δ_H = 7.07 (tt, J = 7.3, 1.2 Hz, 1H, $^{10}H_{Ar}$), 7.32-7.41 (m, 2H, $^9H_{Ar}$ and $^{9'}H_{Ar}$), 7.42 (dd, J = 8.1, 5.0 Hz, 1H, $^2H_{Ar}$), 7.81 (dd, J = 7.5, 1.3 Hz, 2H, $^8H_{Ar}$ and $^{8'}H_{Ar}$), 7.97 (dd, J = 8.2, 1.5 Hz, 1H, $^3H_{Ar}$), 8.29 (dd, J = 5.0, 1.5 Hz, 1H, $^1H_{Ar}$), 10.94 (bs, 1H, N-H).

^{13}C NMR (DMSO-*d6*, 101 MHz) : δ_C = 118.4 ($^8CH_{Ar}$ and $^{8'}CH_{Ar}$), 121.7 ($^2CH_{Ar}$), 122.8 ($^{10}CH_{Ar}$), 126.2 ($^3CH_{Ar}$), 129.0 ($^9CH_{Ar}$ and $^{9'}CH_{Ar}$), 140.0 ($^7C^{IV}$), 141.8 ($^1CH_{Ar}$), 146.5 ($^4C^{IV}$), 153.2 ($^5C^{IV}$) and 161.1 ($^6C^{IV}$).

molecules

MDPI

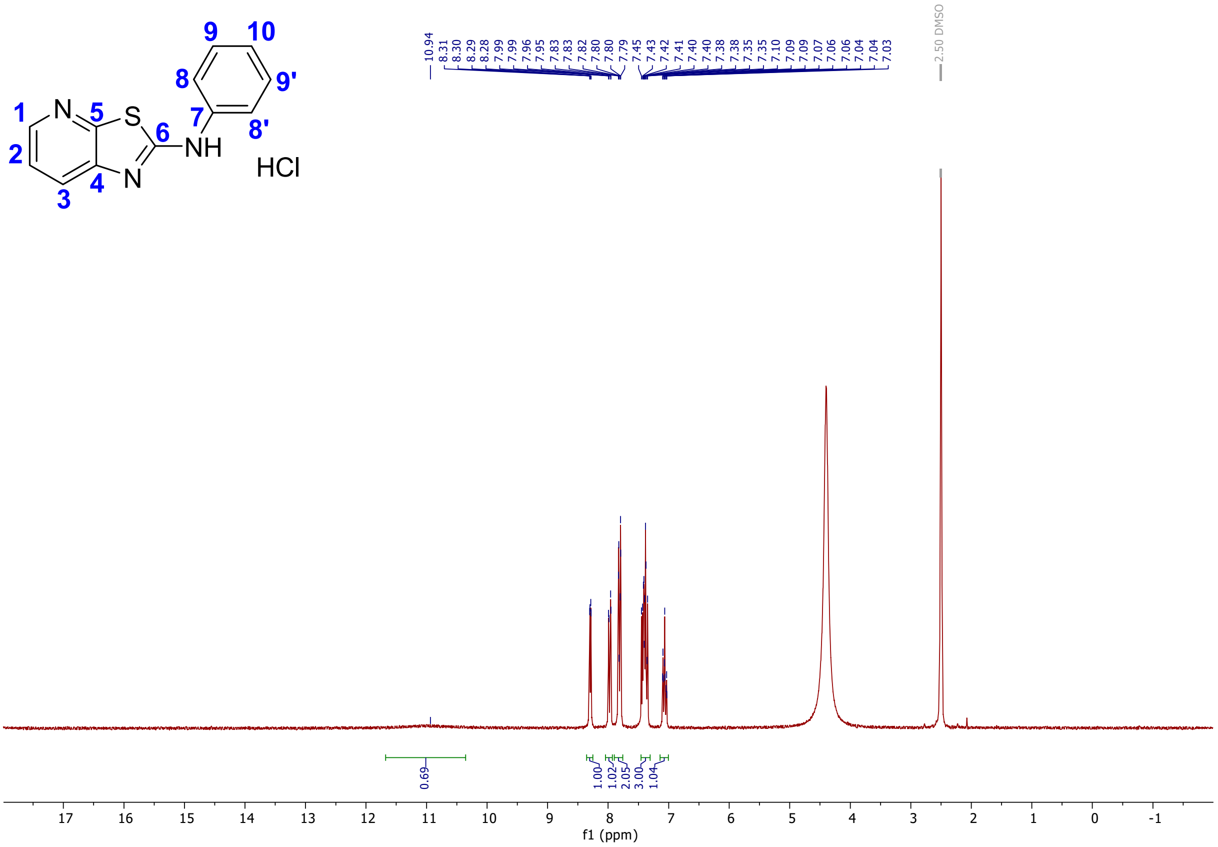

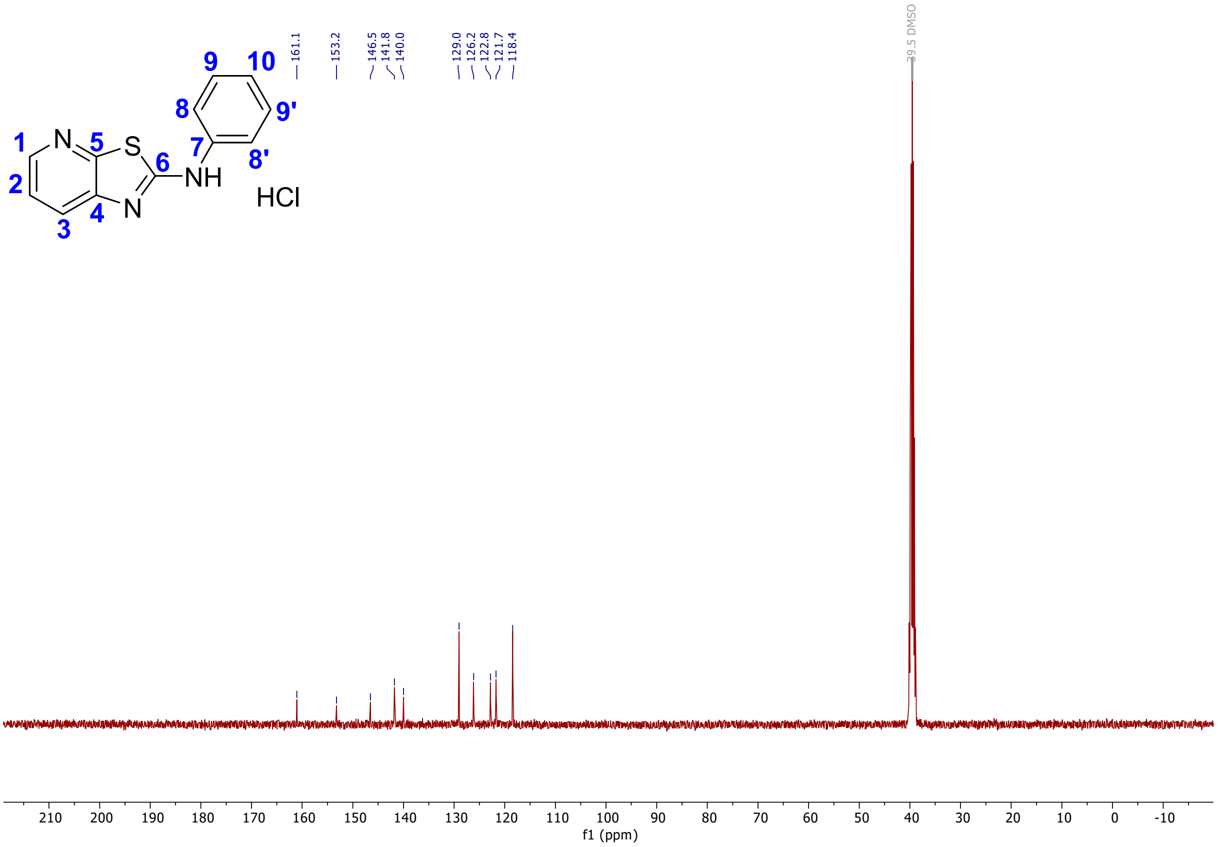

molecules

MDPI

N-(4-chlorophenyl)thiazolo[5,4-*b*]pyridin-2-amine hydrochloride (**4b**)

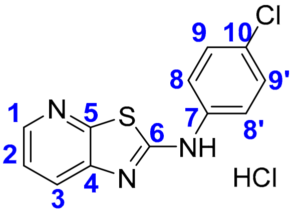

General procedure 1 was applied to 4-chlorophenyl isothiocyanate and 3-amino-2-chloropyridine **2a** to give *N*-(4-chlorophenyl)thiazolo[5,4-*b*]pyridin-2-amine hydrochloride **4b** in 59% yield.

Aspect : beige powder

Melting point : 258°C

HRMS (ESI+): m/z calculated for $C_{12}H_9ClN_3S$ [M + H$^+$] = 262.0200 ; found = 262.0198

^{1}**H NMR (DMSO-*d6*, 400 MHz) :** δ_H = 7.39-7.46 (m, 3H, $^2H_{Ar}$ + $^8H_{Ar}$ + $^{8'}H_{Ar}$), 7.86 (d, J = 8.0 Hz, 2H, $^9H_{Ar}$ and $^{9'}H_{Ar}$), 7.98 (d, J = 8.2 Hz, 1H, $^3H_{Ar}$), 8.31 (d, J = 5.2 Hz, 1H, $^1H_{Ar}$), 11.23 (bs, 1H, N-H).

^{13}**C NMR (DMSO-*d6*, 101 MHz) :** δ_C = 119.8 ($^9CH_{Ar}$ and $^{9'}CH_{Ar}$), 121.8 ($^2CH_{Ar}$), 126.2 ($^{10}C^{IV}$), 126.3 ($^3CH_{Ar}$), 128.9 ($^8CH_{Ar}$ and $^{8'}CH_{Ar}$), 139.0 ($^7C^{IV}$), 142.2 ($^1CH_{Ar}$), 146.3 ($^4C^{IV}$), 153.4 ($^5C^{IV}$) and 160.8 ($^6C^{IV}$).

molecules

MDPI

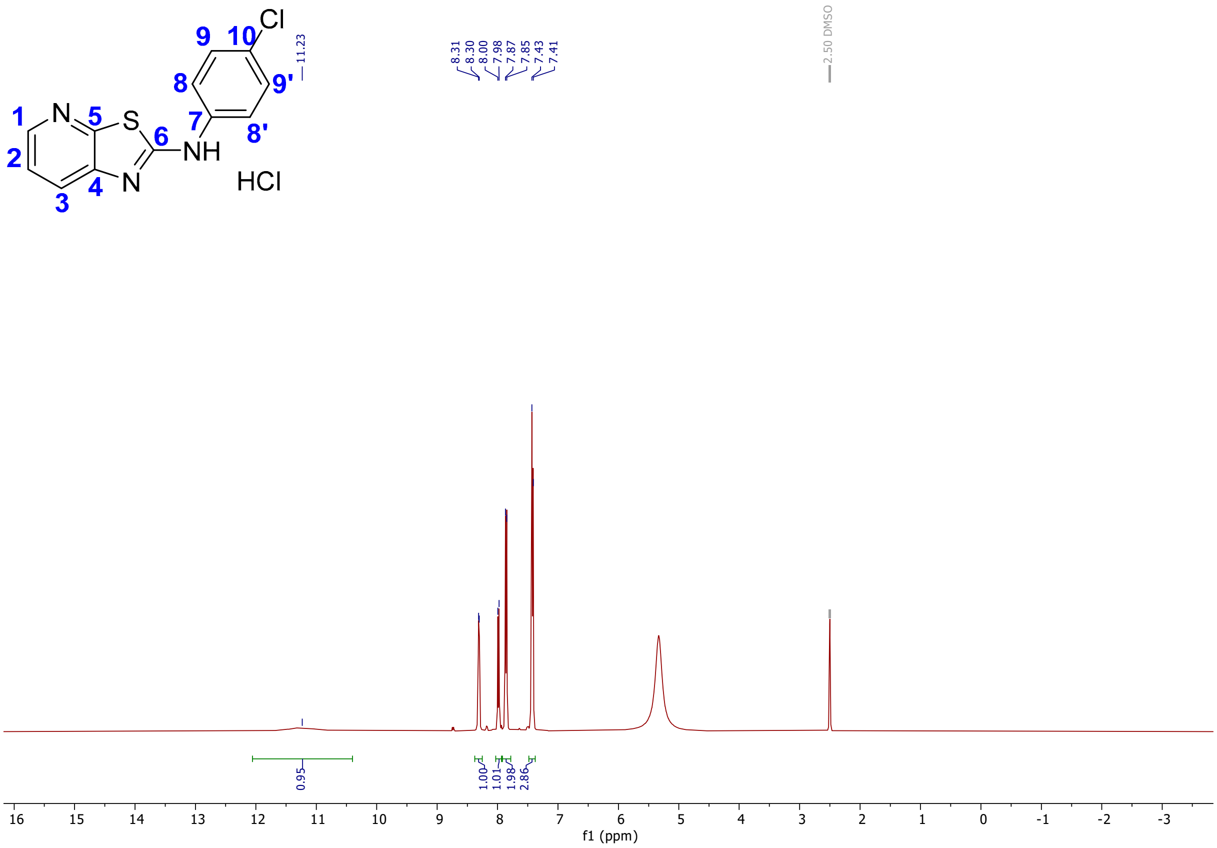

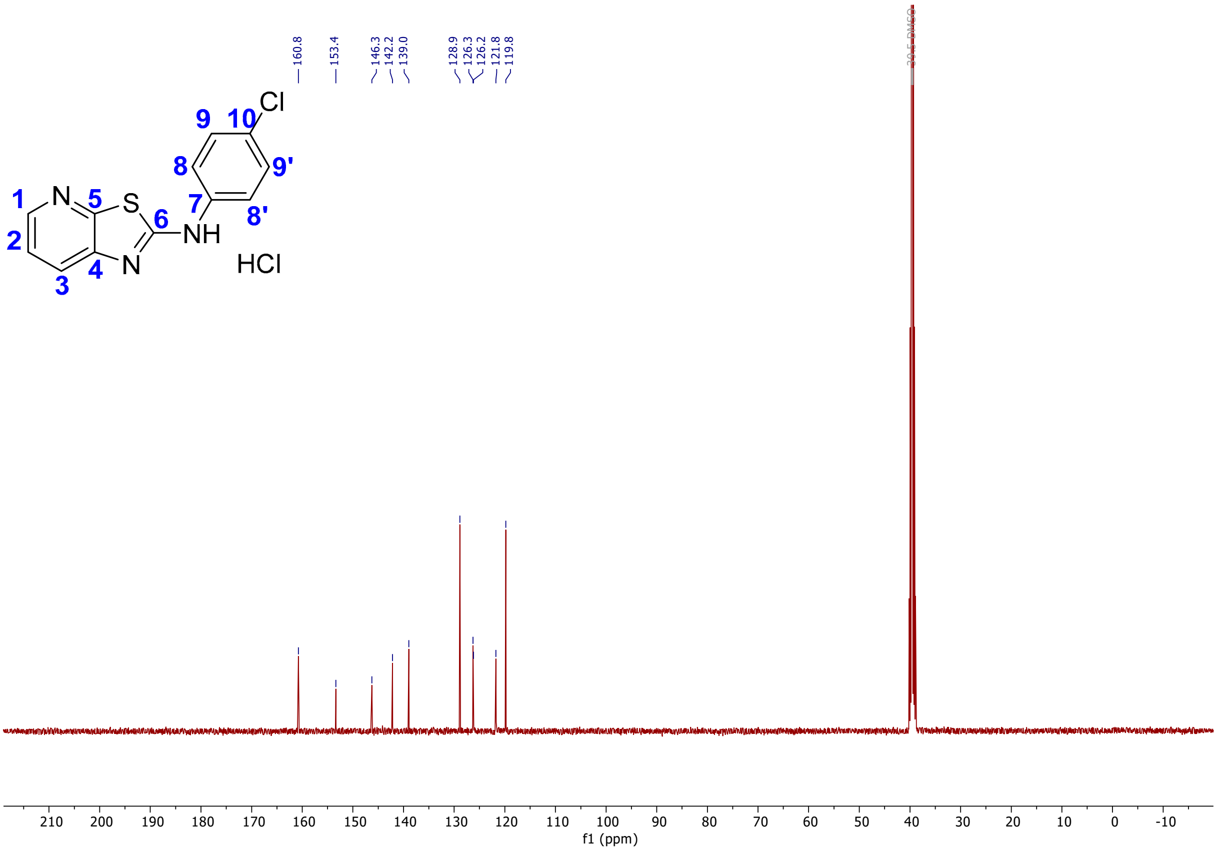

molecules

N-(3,5-bis(trifluoromethyl)phenyl)thiazolo[5,4-b]pyridin-2-amine hydrochloride (**4c**)

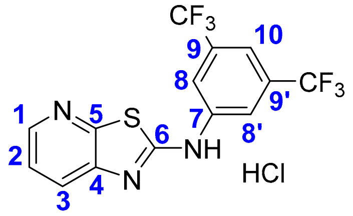

General procedure 3 was applied to 3,5-Bis(trifluoromethyl)phenyl isothiocyanate and 3-amino-2-chloropyridine **2a** to give *N*-(3,5-bis(trifluoromethyl)phenyl)thiazolo[5,4-*b*]pyridin-2-amine hydrochloride **4c** in 54% yield.

Aspect : colourless powder

Melting point : 231°C

HRMS (ESI+): m/z calculated for $C_{14}H_{8}F_{6}N_{3}S$ [M + H^{+}] = 364.0338 ; found = 364.0341

^{1}H NMR (DMSO-*d6*, 400 MHz) : δ_H = 7.43 (dd, J = 8.2, 4.9 Hz, 1H, $^{2}H_{Ar}$), 7.69 (s, 1H, $^{10}H_{Ar}$), 8.03 (d, J = 8.1 Hz, 1H, $^{3}H_{Ar}$), 8.34 (d, J = 4.9 Hz, 1H, $^{1}H_{Ar}$), 8.51 (s, 2H, $^{8}H_{Ar}$ and $^{8'}H_{Ar}$), 11.89 (bs, 1H, N-H).

^{13}C NMR (DMSO-*d6*, 101 MHz) : δ_C = 114.8 ($^{10}CH_{Ar}$), 117.6 ($^{8}CH_{Ar}$ and $^{8'}CH_{Ar}$), 121.8 ($^{2}CH_{Ar}$), 123.3 (q, ^{1}J = 274 Hz, $^{11}CF_3$ and $^{11'}CF_3$), 126.7 ($^{3}CH_{Ar}$), 130.9 (q, ^{2}J = 32 Hz, $^{9}C^{IV}$ and $^{9'}C^{IV}$), 141.8 ($^{7}C^{IV}$), 143.6 ($^{1}CH_{Ar}$), 145.3 ($^{4}C^{IV}$), 153.8 ($^{5}C^{IV}$) and 160.4 ($^{6}C^{IV}$).

^{19}F NMR (DMSO-*d6*, 376 MHz) : δ_F = -61.66.

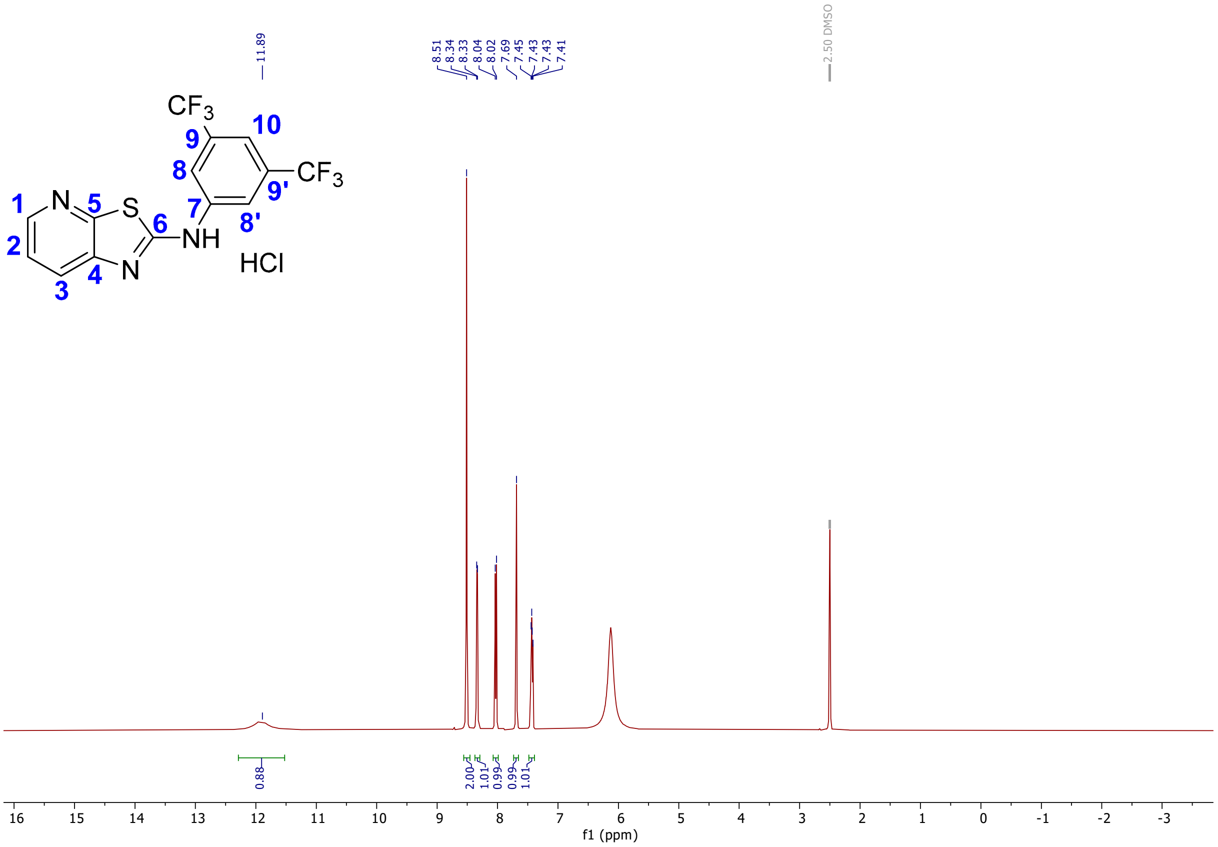
11.89
8.51 8.34 8.33 8.04 8.02 7.69 7.45 7.43 7.43 7.41
2.50 DMSO
CF3
CF3
NH
HCl
N
S
N
1 2 3 4 5 6 7 8 9 10 8' 9'
0.88
2.00 1.01 0.99 0.99 1.01
f1 (ppm)

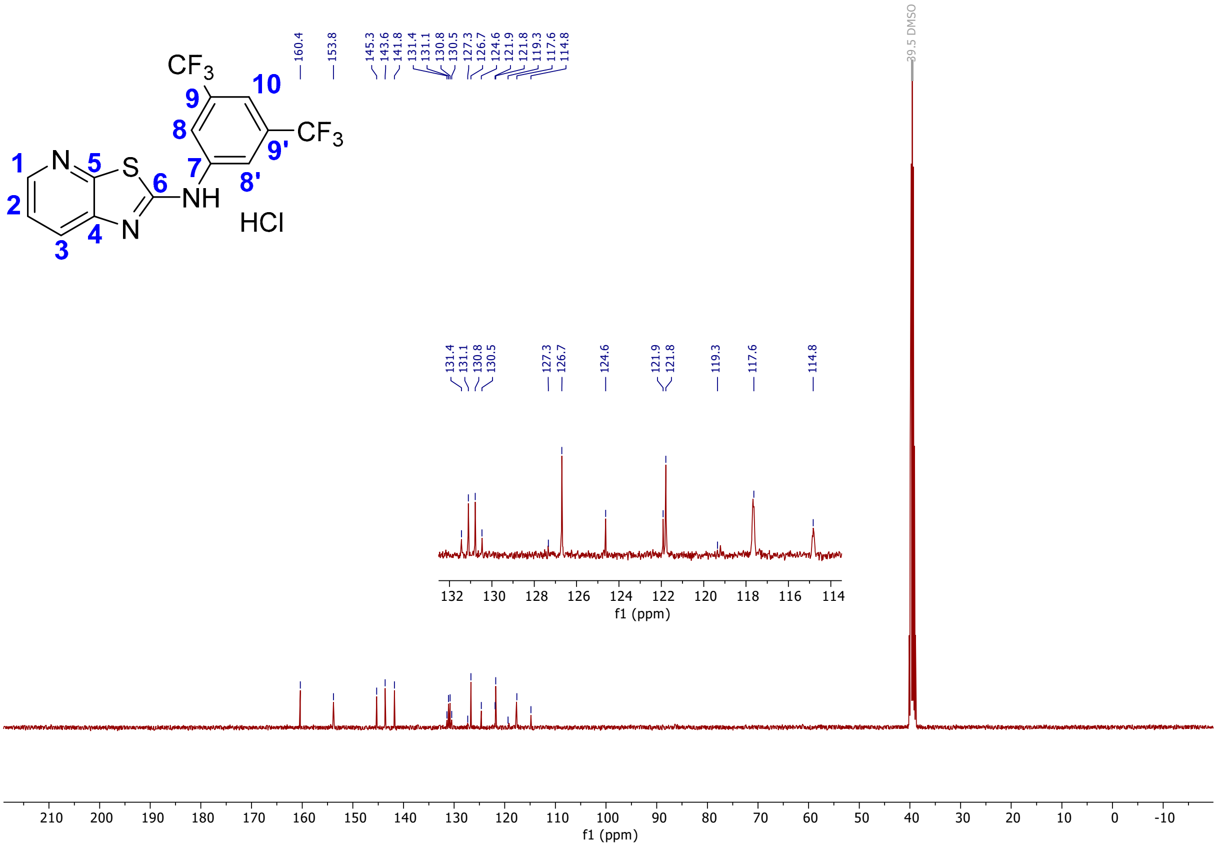
160.4
153.8
145.3 143.6 141.8 131.4 131.1 130.8 130.5 127.3 126.7 124.6 121.9 121.8 119.3 117.6 114.8
39.5 DMSO
CF3
CF3
NH
HCl
N
S
N
1 2 3 4 5 6 7 8 9 10 8' 9'
131.4 131.1 130.8 130.5 127.3 126.7 124.6 121.9 121.8 119.3 117.6 114.8
f1 (ppm)
f1 (ppm)

molecules

MDPI

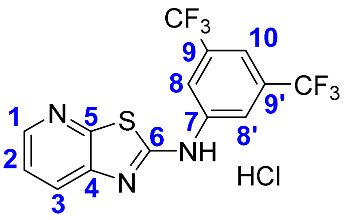

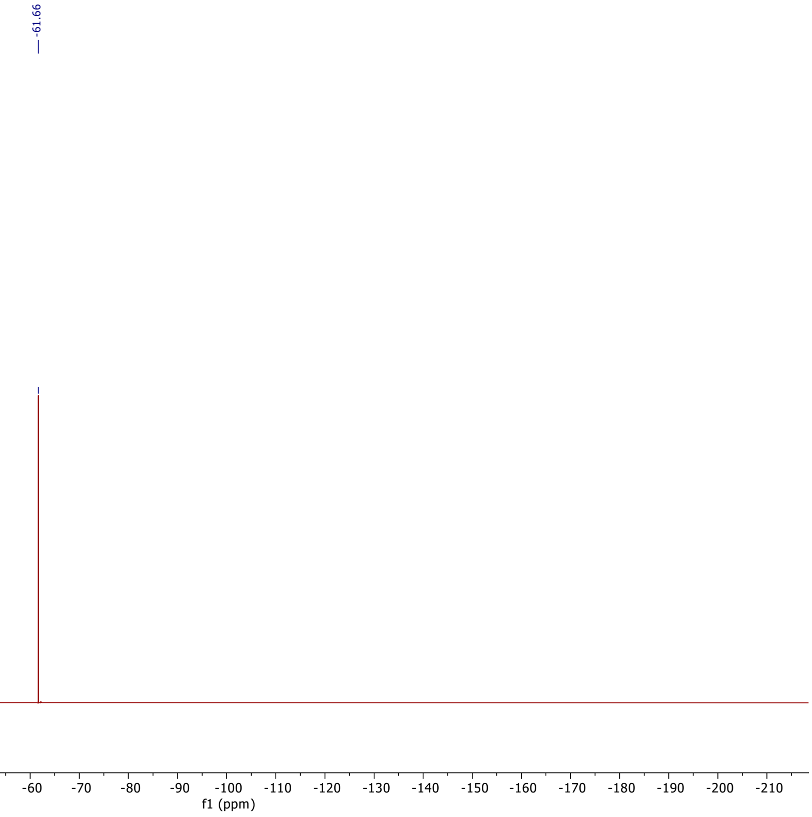

N-(4-methoxyphenyl)thiazolo[5,4-*b*]pyridin-2-amine hydrochloride (**4d**)

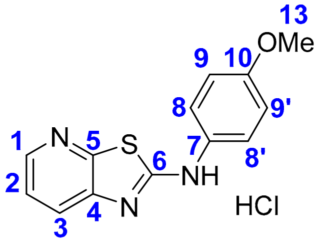

General procedure 1 was applied to 4-methoxyphenyl isothiocyanate and 3-amino-2-chloropyridine **2a** to give *N*-(4-methoxyphenyl)thiazolo[5,4-*b*]pyridin-2-amine hydrochloride **4d** in 54% yield.

Aspect : yellow powder

Melting point : 241°C

HRMS (ESI+): m/z calculated for $C_{13}H_{12}N_3OS$ [M + H$^+$] = 258.0695 ; found = 258.0693

^{1}H NMR (DMSO-*d6*, 400 MHz) : δ_H = 3.74 (s, 3H, $^{11}CH_3$-O), 6.96 (d, J = 7.0 Hz, 2H, $^8CH_{Ar}$ and $^{8'}CH_{Ar}$), 7.41 (dd, J = 8.1, 5.0 Hz, 1H, $^2CH_{Ar}$), 7.69 (d, J = 7.0 Hz, 2H, $^9CH_{Ar}$ and $^{9'}CH_{Ar}$), 7.93 (d, J = 8.1 Hz, 1H, $^3CH_{Ar}$), 8.27 (d, J = 5.1 Hz, 1H, $^1CH_{Ar}$), 10.93 (bs, 1H, N-H).

^{13}C NMR (DMSO-*d6*, 101 MHz) : δ_C = 55.3 ($^{11}CH_3$-O), 114.3 ($^8CH_{ar}$ and $^{8'}CH_{Ar}$), 120.5 ($^9CH_{Ar}$ and $^{9'}CH_{Ar}$), 121.8 ($^2CH_{Ar}$), 125.8 ($^3CH_{Ar}$), 133.1 ($^7C^{IV}$), 141.1 ($^1CH_{Ar}$), 146.7 ($^4C^{IV}$), 152.8 ($^5C^{IV}$), 155.3 ($^{10}C^{IV}$) and 161.6 ($^6C^{IV}$).

molecules

MDPI

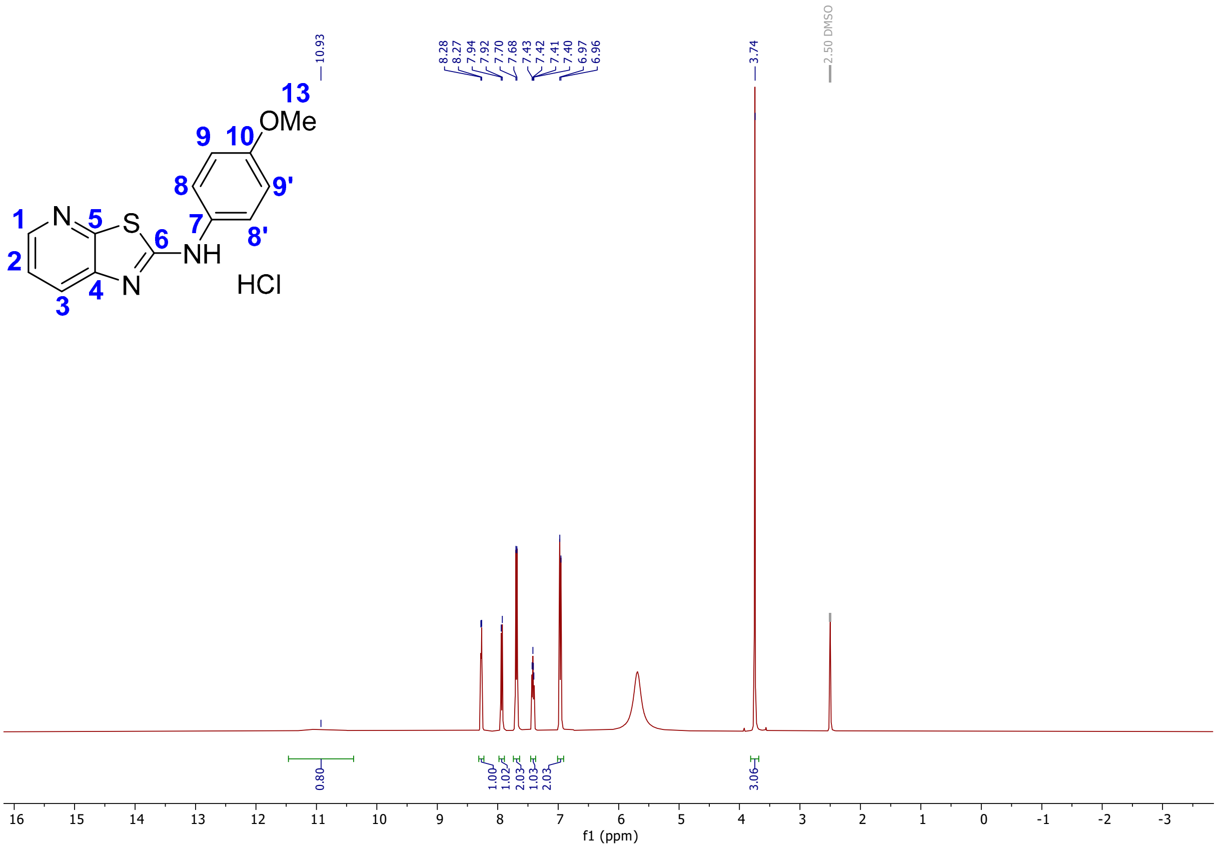

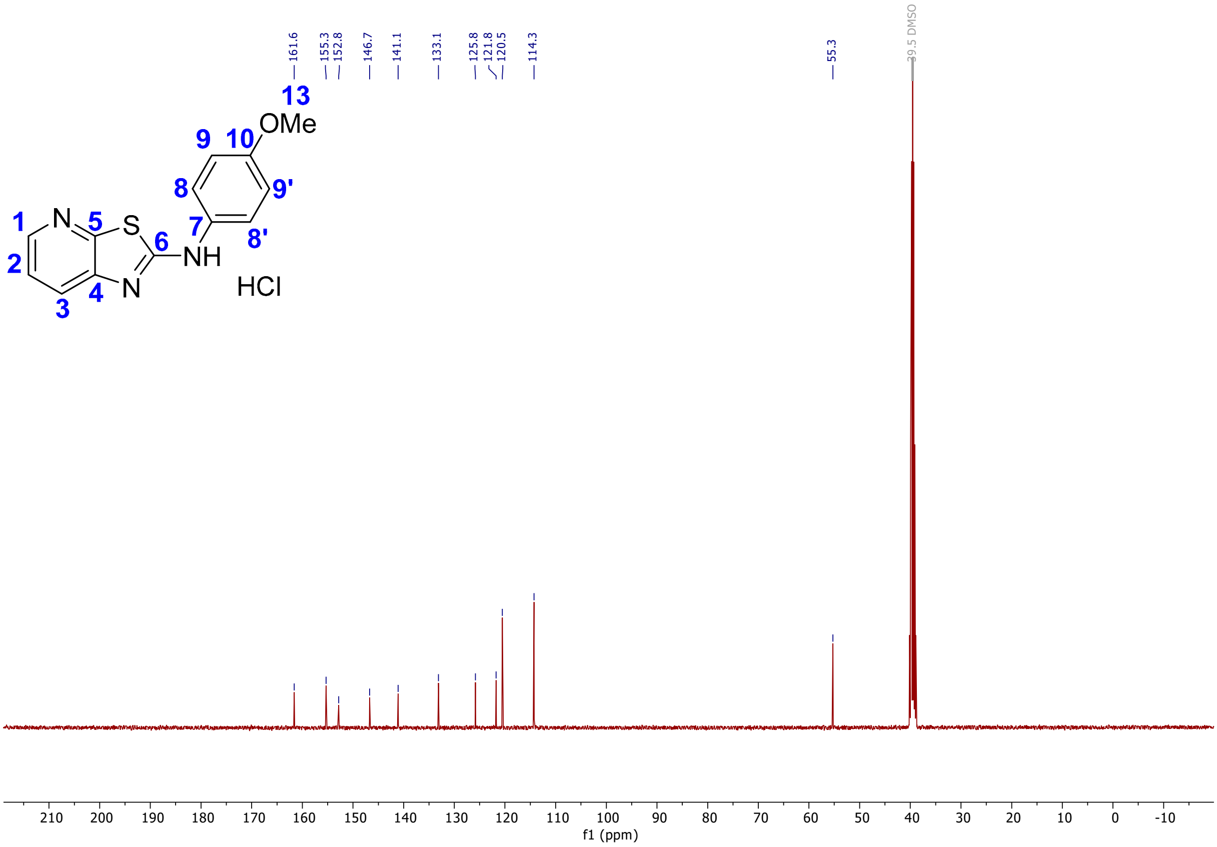

molecules MDPI

N-(4-bromophenyl)thiazolo[5,4-*b*]pyridin-2-amine hydrochloride (**4e**)

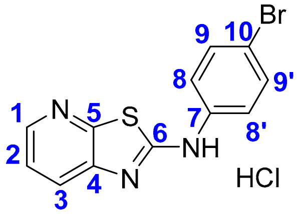

General procedure 1 was applied to 4-bromophenyl isothiocyanate and 3-amino-2-chloropyridine **2a** to give *N*-(4-bromophenyl)thiazolo[5,4-*b*]pyridin-2-amine hydrochloride **4e** in 66% yield.

Aspect : beige powder

Melting point : 264°C (decomposition.)

HRMS (ESI+): m/z calculated for $C_{12}H_9BrN_3S$ [M + H+] = 305.9695 ; found = 305.9698

^{1}H NMR (DMSO-*d6*, 400 MHz) : δ_H = 7.41 (dd, J = 8.1, 4.9 Hz, 1H, $^2H_{Ar}$), 7.55 (d, J = 8.8 Hz, 2H, $^8H_{Ar}$ and $^{8'}H_{Ar}$), 7.80 (d, J = 8.9 Hz, 2H, $^9H_{Ar}$ and $^{9'}H_{Ar}$), 7.97 (dd, J = 8.2, 1.6 Hz, 1H, $^3H_{Ar}$), 8.30 (dd, J = 4.9, 1.6 Hz, 1H, $^1H_{Ar}$), 11.16 (bs, 1H, N-H).

^{13}C NMR (DMSO-*d6*, 101 MHz) : δ_C = 114.1 ($^{10}C^{IV}$), 120.2 ($^9CH_{Ar}$ and $^{9'}CH_{Ar}$), 121.7 ($^2CH_{Ar}$), 126.1 ($^3CH_{Ar}$), 131.8 ($^8CH_{Ar}$ and $^{8'}CH_{Ar}$), 139.4 ($^7C^{IV}$), 142.5 ($^1CH_{Ar}$), 146.1 ($^4C^{IV}$), 153.6 ($^5C^{IV}$) and 160.6 ($^6C^{IV}$).

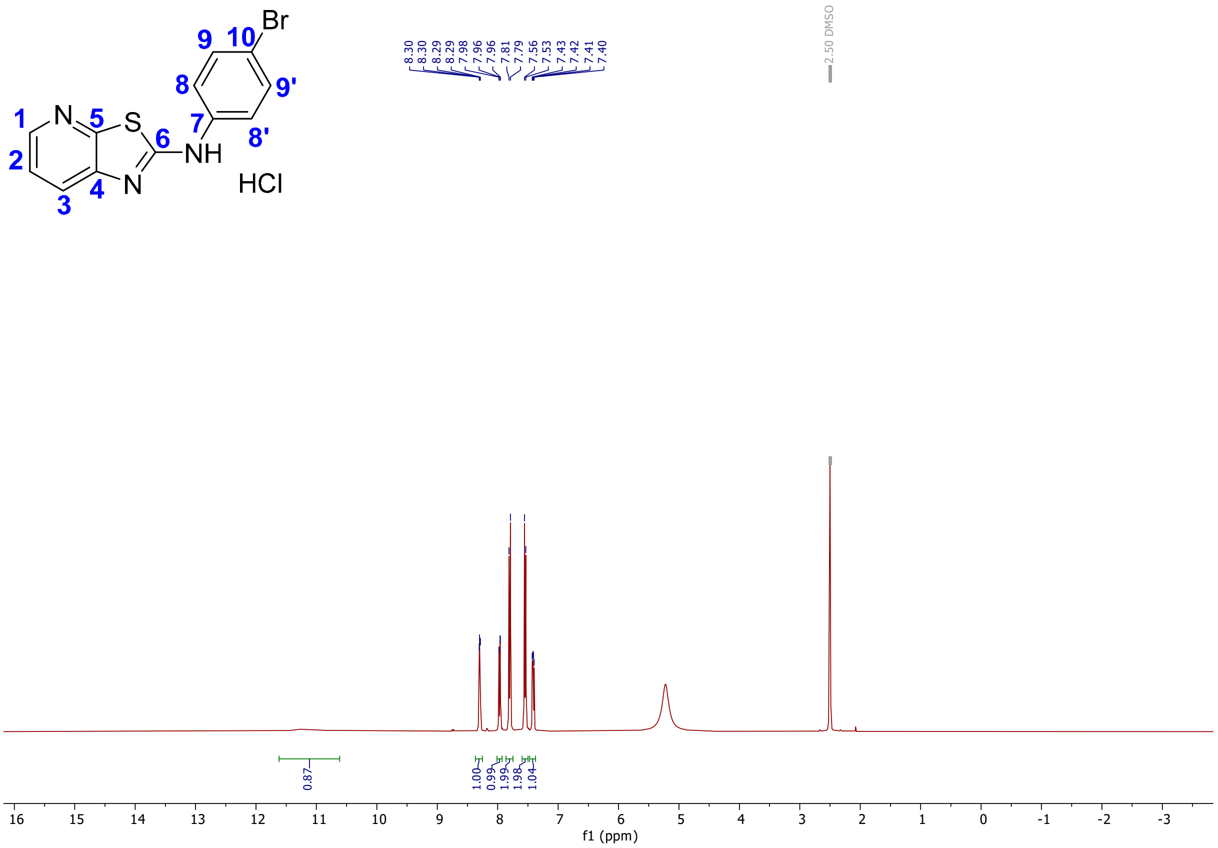
Br
9
10
8
9'
1
N
5
S
7
6
NH
8'
2
4
N
3
HCl
8.30
8.30
8.29
8.29
7.98
7.96
7.96
7.81
7.79
7.56
7.53
7.43
7.42
7.41
7.40
2.50 DMSO
0.87
1.00
0.99
1.99
1.98
1.04
16
15
14
13
12
11
10
9
8
7
6
5
4
3
2
1
0
-1
-2
-3
f1 (ppm)

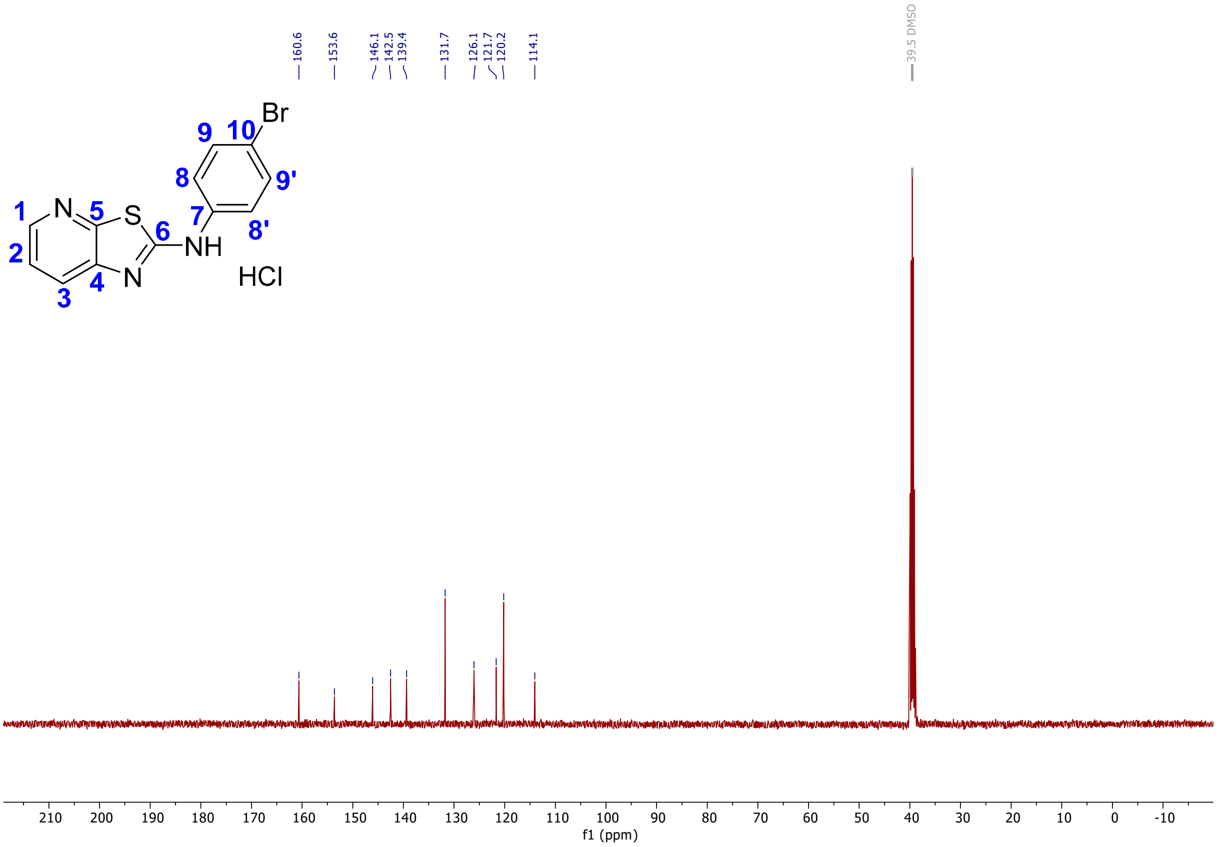
160.6
153.6
146.1
142.5
139.4
131.7
126.1
121.7
120.2
114.1
39.5 DMSO
Br
9
10
8
9'
1
N
5
S
7
6
NH
8'
2
4
N
3
HCl
210
200
190
180
170
160
150
140
130
120
110
100
90
80
70
60
50
40
30
20
10
0
-10
f1 (ppm)

molecules

MDPI

N-(3-bromophenyl)thiazolo[5,4-*b*]pyridin-2-amine hydrochloride (**4f**)

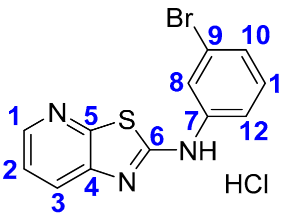

General procedure 1 was applied to 3-bromophenyl isothiocyanate and 3-amino-2-chloropyridine **2a** to give *N*-(3-bromophenyl)thiazolo[5,4-*b*]pyridin-2-amine hydrochloride **4f** in 58% yield.

Aspect : yellowish powder

Melting point : 231°C

HRMS (ESI+) : calculated for $C_{12}H_9BrN_3S$ [M + H$^+$] = 305.9695 ; found = 305.9689

^{1}H NMR (DMSO-*d6*, 400 MHz) : δ_H = 7.23 (d, J = 8.1 Hz, 1H, $^{12}H_{Ar}$), 7.33 (t, J = 8.1 Hz, 1H, $^{11}H_{Ar}$), 7.41 (dd, J = 8.4, 5.2 Hz, 1H, $^2H_{Ar}$), 7.71 (d, J = 8.2 Hz, 1H, $^{10}H_{Ar}$), 7.99 (d, J = 8.1 Hz, 1H, $^3H_{Ar}$), 8.18 (s, 1H, $^8H_{Ar}$), 8.29 (d, J = 5.3 Hz, 1H, $^1H_{Ar}$), 11.12 (bs, 1H, N-H).

^{13}C NMR (DMSO-*d6*, 101 MHz) : δ_C = 117.2 ($^{10}CH_{Ar}$), 120.5 ($^8CH_{Ar}$), 121.8 ($^2CH_{Ar}$), 121.9 ($^9C^{IV}$), 125.2 ($^{12}CH_{Ar}$), 127.0 ($^3CH_{Ar}$), 130.9 ($^{11}CH_{Ar}$), 141.5 ($^1CH_{Ar}$), 141.6 ($^7C^{IV}$), 146.5 ($^4C^{IV}$), 152.7 ($^5C^{IV}$) and 160.8 ($^6C^{IV}$).

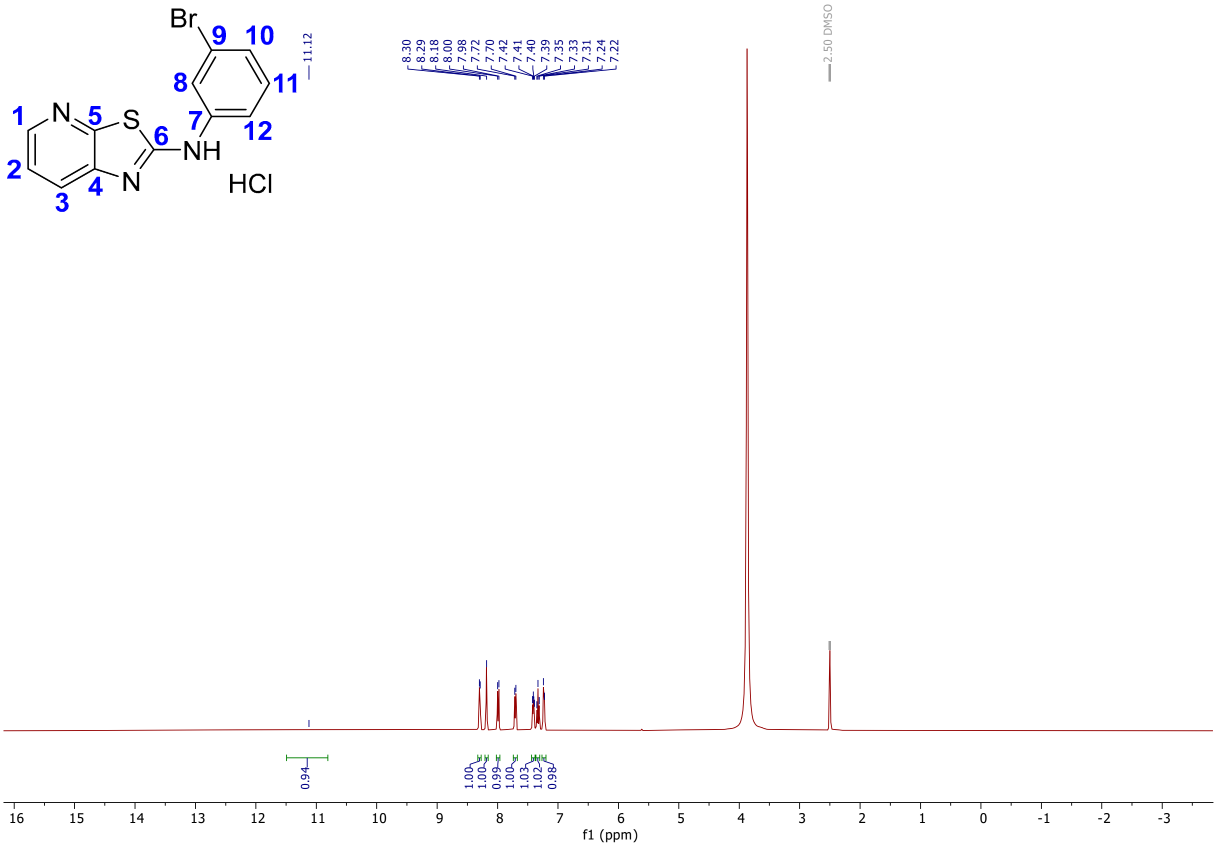
Br
9 10
8
11
1 N 5 S
6
7 NH
12
2
3 4 N
HCl
11.12
8.30
8.29
8.18
8.00
7.98
7.72
7.70
7.42
7.41
7.40
7.39
7.35
7.33
7.31
7.24
7.22
2.50 DMSO
0.94
1.00
1.00
0.99
1.00
1.03
1.02
0.98
f1 (ppm)

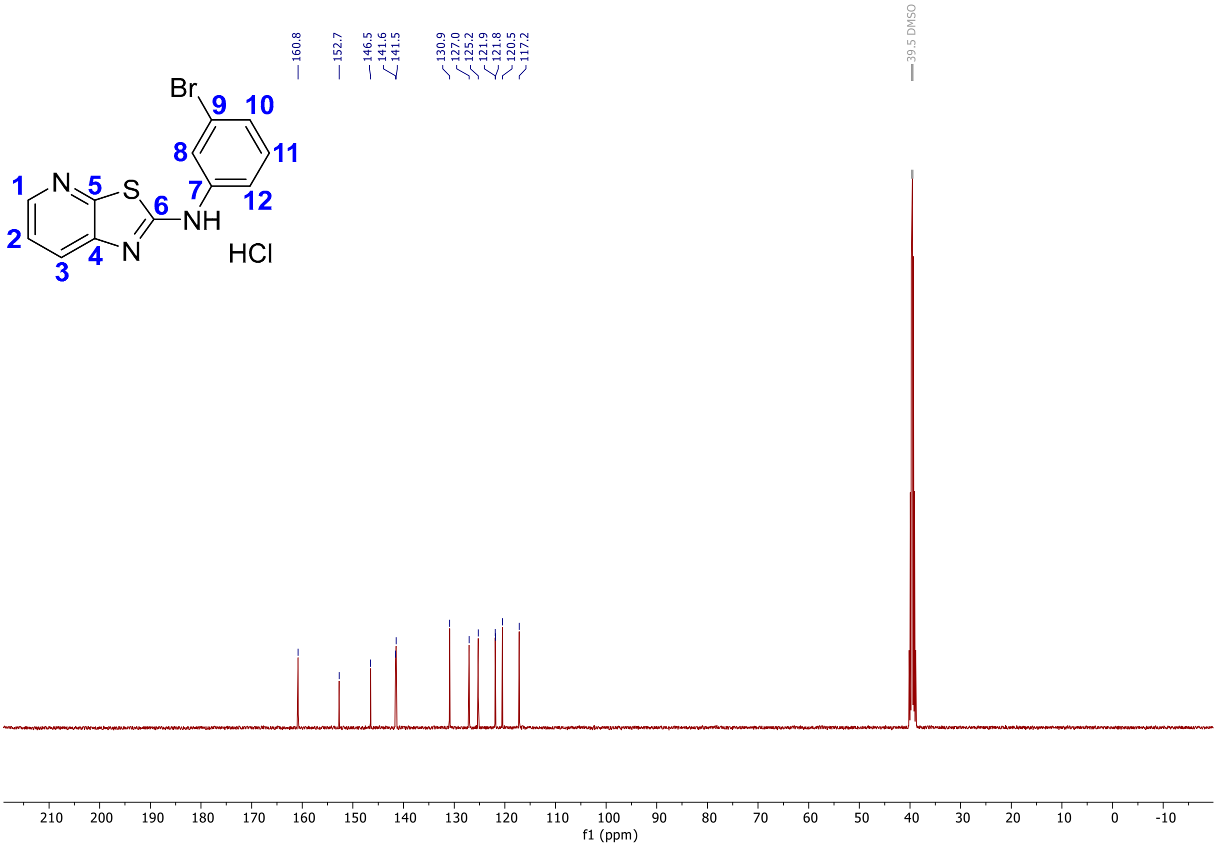
160.8
152.7
146.5
141.6
141.5
130.9
127.0
125.2
121.9
121.8
120.5
117.2
39.5 DMSO
Br
9 10
8
11
1 N 5 S
6
7 NH
12
2
3 4 N
HCl
f1 (ppm)

molecules

N-(3-chlorophenyl)thiazolo[5,4-*b*]pyridin-2-amine hydrochloride (**4g**)

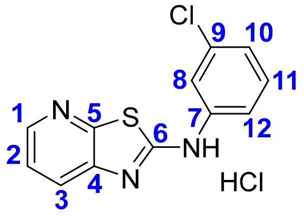

General procedure 1 was applied to 3-chlorophenyl isothiocyanate and 3-amino-2-chloropyridine **2a** to give *N*-(3-chlorophenyl)thiazolo[5,4-*b*]pyridin-2-amine hydrochloride **4g** in 63% yield.

Aspect : beige powder

Melting point : 205°C.

HRMS (ESI+): m/z calculated for $C_{12}H_9ClN_3S$ [M + H$^+$] = 262.0200 ; found = 262.0202

^{1}H NMR (DMSO-*d6*, 400 MHz) : δ_H = 7.10 (dd, J = 7.8, 2.4 Hz, 1H, $^{12}H_{Ar}$), 7.39 (t, J = 8.2 Hz, 1H, $^{11}H_{Ar}$), 7.44 (dd, J = 8.0, 4.9 Hz, 1H, $^{2}H_{Ar}$), 7.67 (dd, J = 8.2, 2.6 Hz, 1H, $^{10}H_{Ar}$), 8.03 (dd, J = 8.2, 1.6 Hz, 1H, $^{3}H_{Ar}$), 8.07 (m, 1H, $^{8}H_{Ar}$), 8.32 (dd, J = 4.9, 1.6 Hz, 1H, $^{1}H_{Ar}$), 11.32 (bs, 1H, N-H).

^{13}C NMR (DMSO-*d6*, 101 MHz) : δ_C = 116.7 ($^{10}CH_{Ar}$), 117.6 ($^{8}CH_{Ar}$), 121.8 ($^{2}CH_{Ar}$), 122.3 ($^{12}CH_{Ar}$), 126.5 ($^{3}CH_{Ar}$), 130.6 ($^{11}CH_{Ar}$), 133.3 ($^{9}C^{IV}$), 141.4 ($^{7}C^{IV}$), 142.4 ($^{1}CH_{Ar}$), 146.1 ($^{4}C^{IV}$), 153.4 ($^{5}C^{IV}$) and 160.7 ($^{6}C^{IV}$).

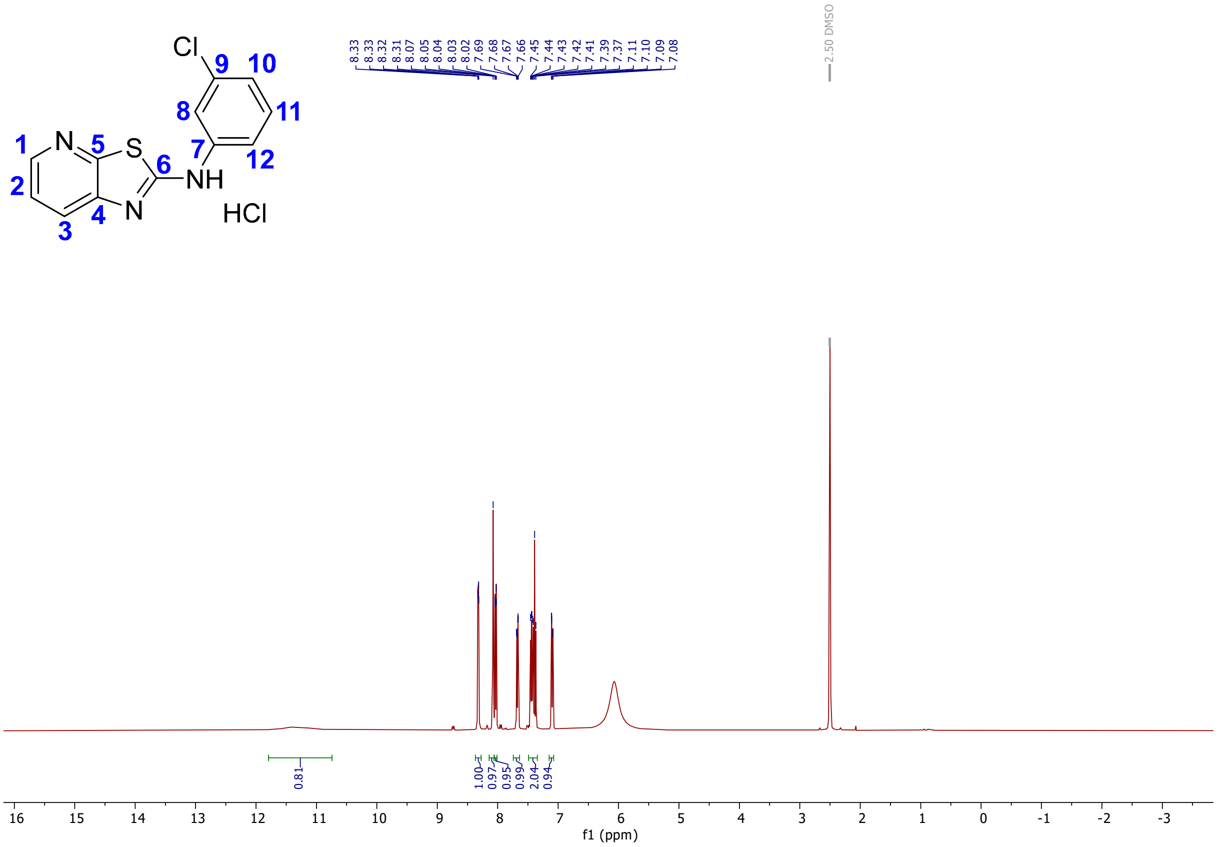
Cl
9
10
8
11
1
N
5
S
7
6
NH
12
2
3
4
N
HCl
8.33
8.33
8.32
8.31
8.07
8.05
8.04
8.03
8.02
7.69
7.68
7.67
7.66
7.45
7.44
7.43
7.42
7.41
7.39
7.37
7.11
7.10
7.09
7.08
2.50 DMSO
0.81
1.00
0.97
0.95
0.99
2.04
0.94
f1 (ppm)

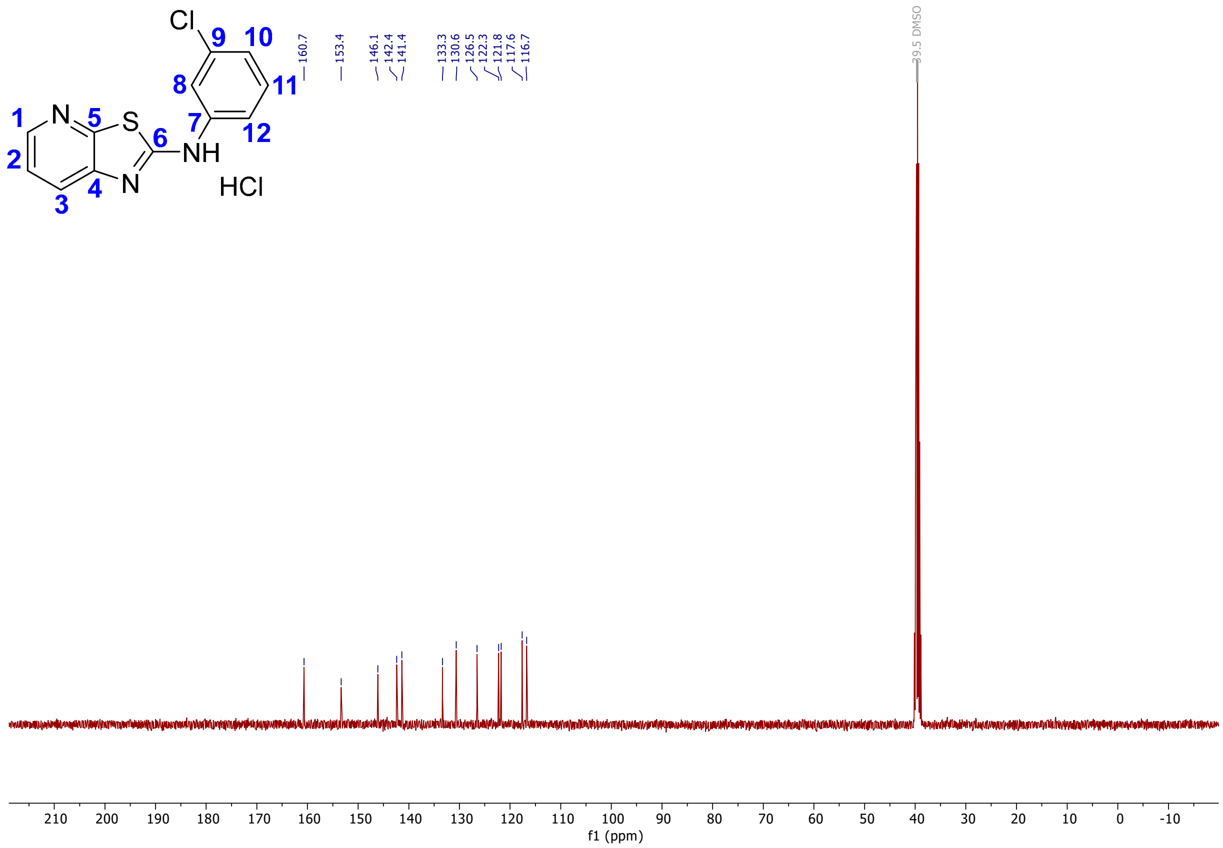
Cl
9
10
8
11
1
N
5
S
7
6
NH
12
2
3
4
N
HCl
160.7
153.4
146.1
142.4
141.4
133.3
130.6
126.5
122.3
121.8
117.6
116.7
39.5 DMSO
f1 (ppm)

N-(3,5-dichlorophenyl)thiazolo[5,4-*b*]pyridin-2-amine hydrochloride (**4h**)

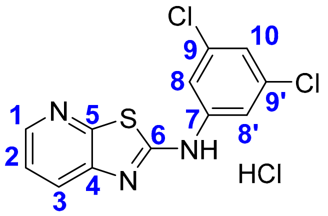

General procedure 3 was applied to 3,5-dichlorophenyl isothiocyanate and 3-amino-2-chloropyridine **2a** to give *N*-(3,5-dichlorophenyl)thiazolo[5,4-*b*]pyridin-2-amine hydrochloride **4h** in 66% yield.

Aspect : beige powder

Melting point : 265°C (decomposition)

HRMS (ESI+): m/z calculated for $C_{12}H_8Cl_2N_3S$ [M + H$^+$] = 295.9811 ; found = 295.9810

^{1}H NMR (DMSO-*d6*, 400 MHz) : δ_H = 7.22 (t, J = 1.8 Hz, 1H, $^{10}H_{Ar}$), 7.43 (dd, J = 8.2, 4.9 Hz, 1H, $^2H_{Ar}$), 7.91 (d, J = 1.8 Hz, 2H, $^8H_{Ar}$ and $^{8'}H_{Ar}$), 8.05 (dd, J = 8.2, 1.6 Hz, 1H, $^3H_{Ar}$), 8.33 (dd, J = 4.9, 1.6 Hz, 1H, $^1H_{Ar}$), 11.54 (bs, 1H, N-H).

^{13}C NMR (DMSO-*d6*, 101 MHz) : δ_C = 116.2 ($^8CH_{Ar}$ and $^{8'}CH_{Ar}$), 121.5 ($^{10}CH_{Ar}$), 121.8 ($^2CH_{Ar}$), 126.6 ($^3CH_{Ar}$), 134.3 ($^9C^{IV}$ and $^{9'}C^{IV}$), 142.2 ($^7C^{IV}$), 143.2 ($^1CH_{Ar}$), 145.6 ($^4C^{IV}$), 153.7 ($^5C^{IV}$) and 160.3 ($^6C^{IV}$).

molecules

MDPI

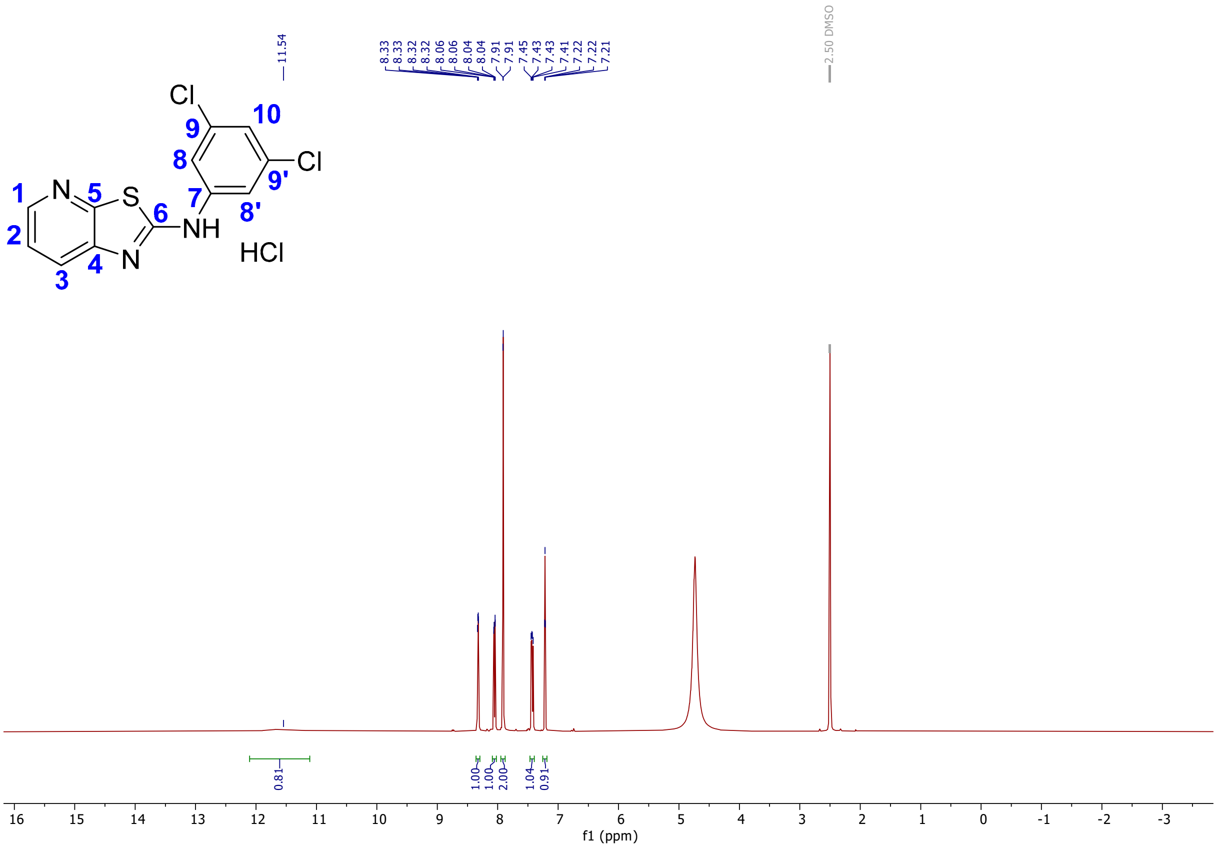

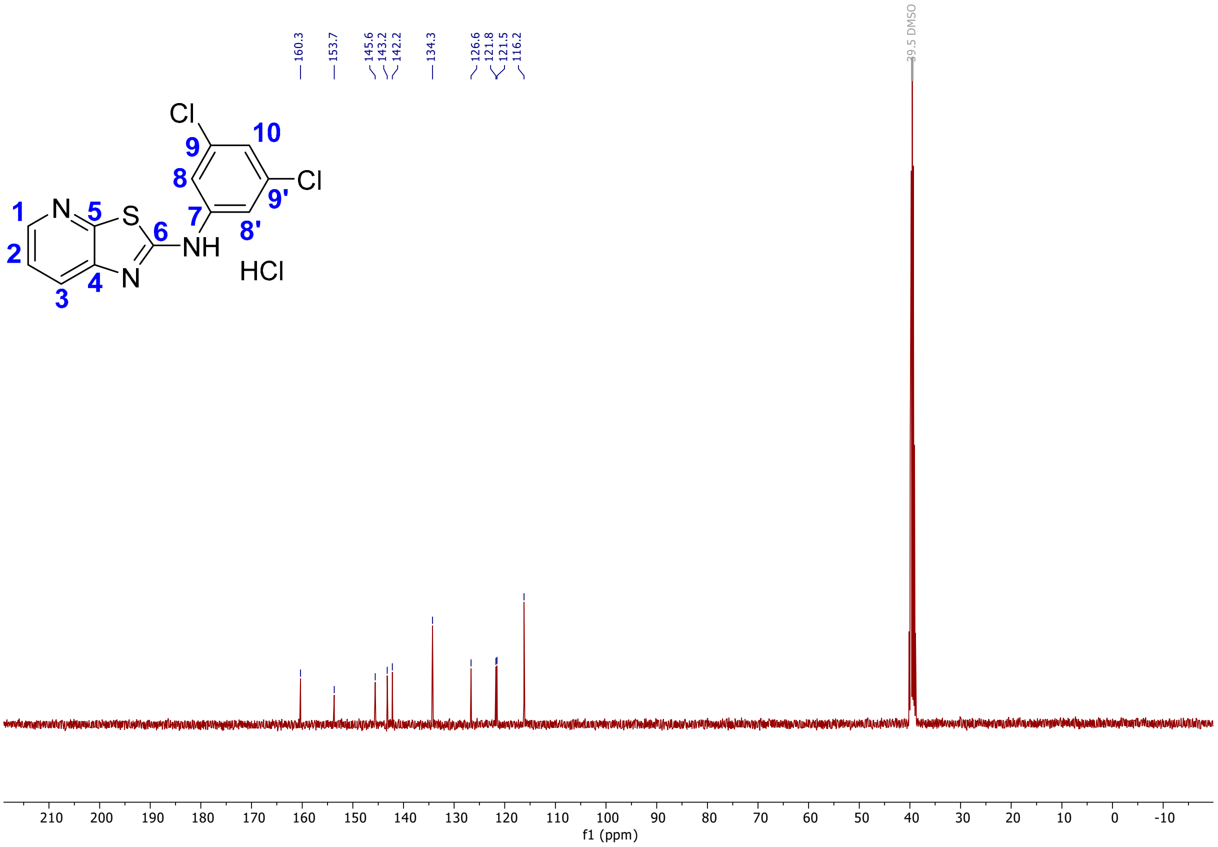

molecules

N-(ethyl 4-aminobenzoate)thiazolo[5,4-*b*]pyridin-2-amine hydrochloride (**4i**)

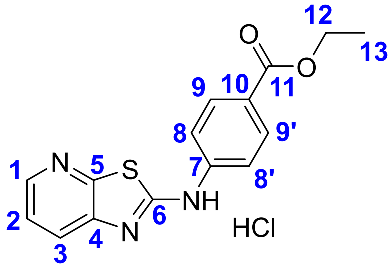

General procedure 3 was applied to ethyl 4-isothiocyanatobenzoate and 3-amino-2-chloropyridine **2a** to give *N*-(ethyl 4-aminobenzoate)thiazolo[5,4-*b*]pyridin-2-amine hydrochloride **4i** in 55% yield.

Aspect : beige powder

Melting point : 230°C.

HRMS (ESI+): m/z calculated for $C_{15}H_{14}N_3O_2S$ [M + H$^+$] = 300.0801 ; found = 300.0801

^{1}H NMR (DMSO-*d6*, 400 MHz) : δ_H = 1.31 (t, J = 7.1 Hz, 3H, $^{13}CH_3$), 4.28 (q, J = 7.1 Hz, 2H, $^{12}CH_2$), 7.45 (dd, J = 8.1, 4.9 Hz, 1H, $^2H_{Ar}$), 7.96 (s, 4H, H_{Ar}), 8.04 (d, J = 8.3 Hz, 1H, $^3H_{Ar}$), 8.34 (d, J = 4.9 Hz, 1H, $^1H_{Ar}$), 11.48 (bs, 1H, N-H).

^{13}C NMR (DMSO-*d6*, 101 MHz) : δ_C = 14.3 ($^{13}CH_3$), 60.4 ($^{12}CH_2$), 117.6 (2xCH_{Ar}), 121.8 (^{2}CH), 123.5 ($^{10}C^{IV}$), 126.8 (^{3}CH), 130.5 (2xCH_{Ar}), 142.6 (^{1}CH), 144.2 ($^7C^{IV}$), 146.1 ($^4C^{IV}$), 153.5 ($^5C^{IV}$), 160.5 ($^6C^{IV}$) and 165.3 ($^{11}C^{IV}$=O).

molecules

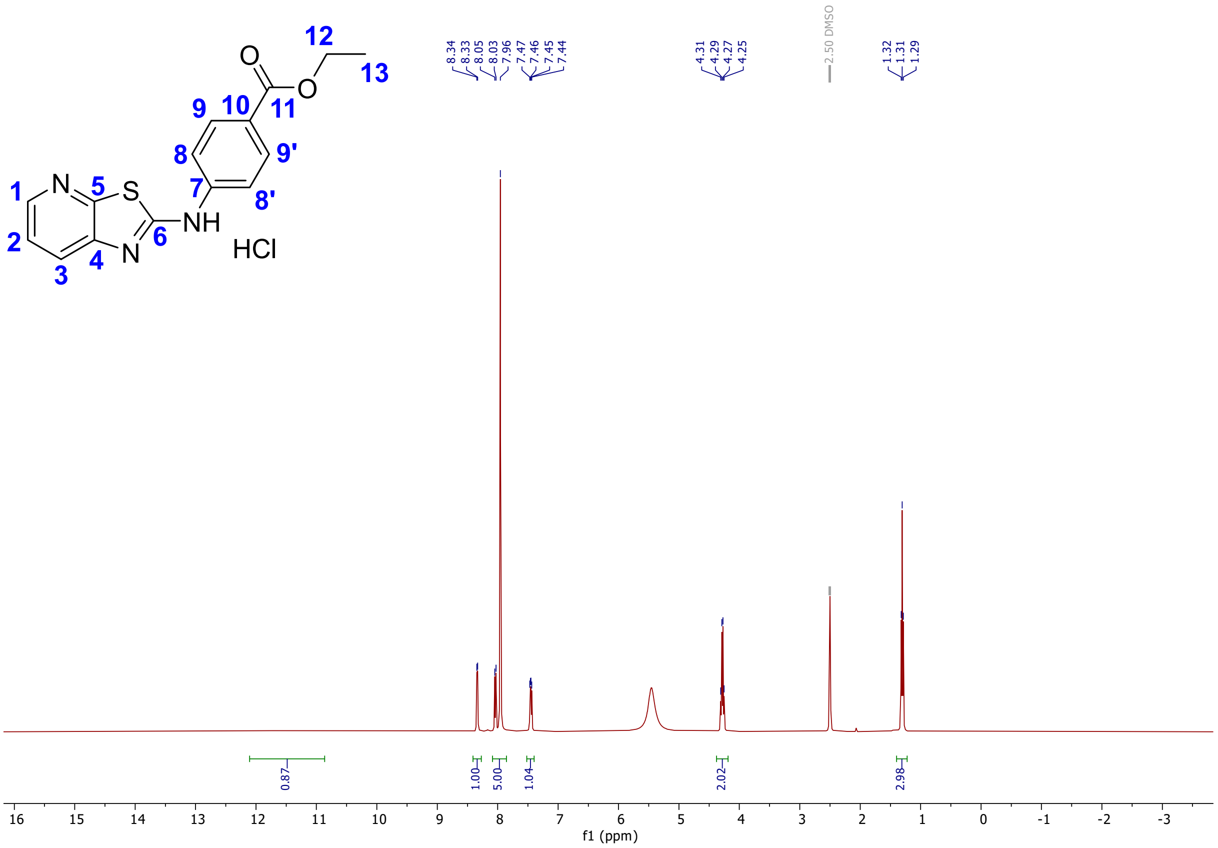

8.34
8.33
8.05
8.03
7.96
7.47
7.46
7.45
7.44
4.31
4.29
4.27
4.25
2.50 DMSO
1.32
1.31
1.29
0.87
1.00
5.00
1.04
2.02
2.98
f1 (ppm)
HCl

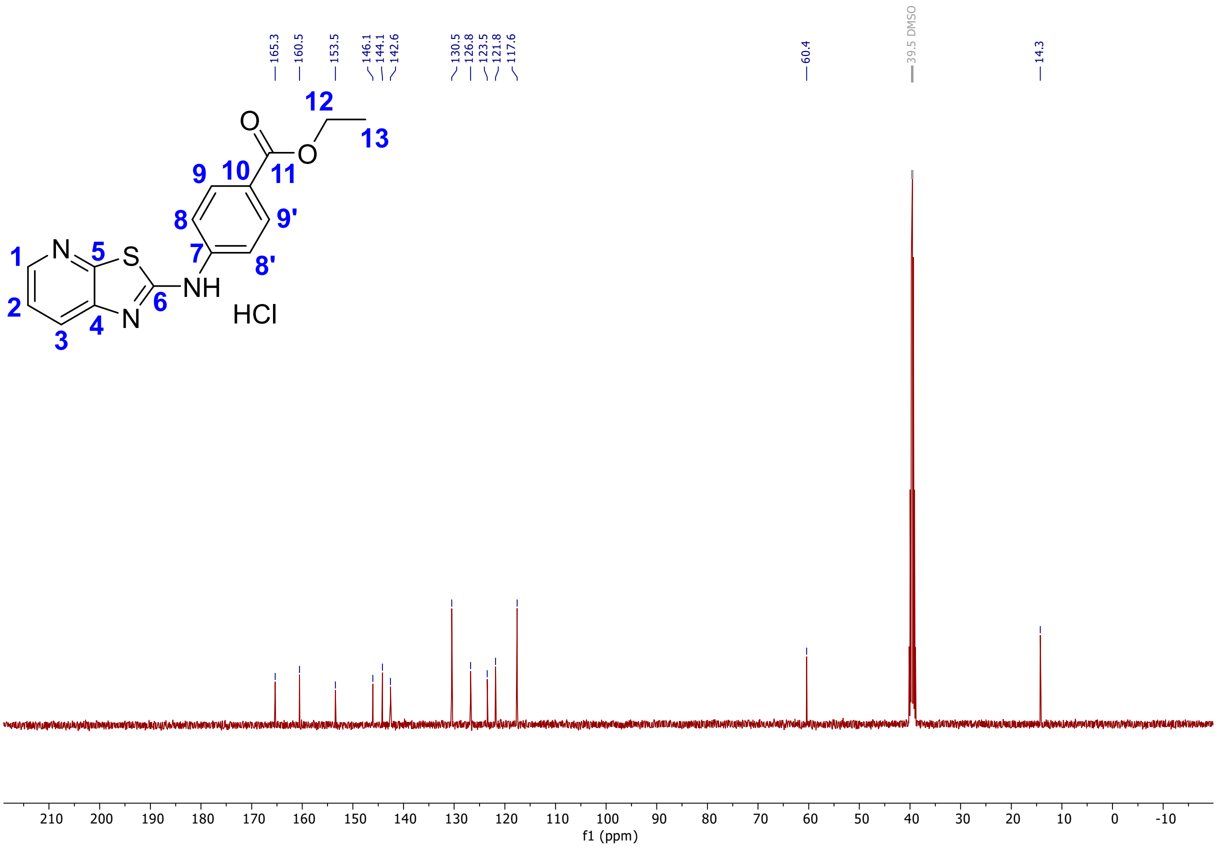

165.3
160.5
153.5
146.1
144.1
142.6
130.5
126.8
123.5
121.8
117.6
60.4
39.5 DMSO
14.3
f1 (ppm)
HCl

molecules

N-methylthiazolo[5,4-*b*]pyridin-2-amine hydrochloride (**4j**)

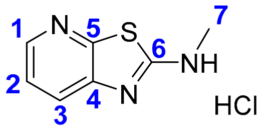

General procedure 1 was applied to methyl isothiocyanate and 3-amino-2-chloropyridine **2a** to give *N*-methylthiazolo[5,4-*b*]pyridin-2-amine hydrochloride **4j** in 50% yield.

Aspect : beige powder

Melting point : 239°C (lit. 264-265) [41]

HRMS (ESI+): m/z calculated for $C_7H_8N_3S$ [M + H^+] = 166.0433 ; found = 166.0438

^{1}H NMR (DMSO-*d6*, 400 MHz) : δ_H = 3.06 (s, 3H, $^7CH_3$), 7.43 (dd, J = 8.1, 5.0 Hz, 1H, $^2H_{Ar}$), 7.88 (d, J = 8.1 Hz, $^3H_{Ar}$), 8.27 (d, J = 5.0 Hz, 1H, $^1H_{Ar}$), 9.69 (bs, 1H, N-H).

^{13}C NMR (DMSO-*d6*, 101 MHz) : δ_C = 30.9 ($^7CH_3$), 122.0 ($^2CH_{Ar}$), 123.7 ($^3CH_{Ar}$), 141.6 ($^1CH_{Ar}$), 142.4 ($^4C^{IV}$), 150.6 ($^5C^{IV}$) and 165.9 ($^6C^{IV}$).

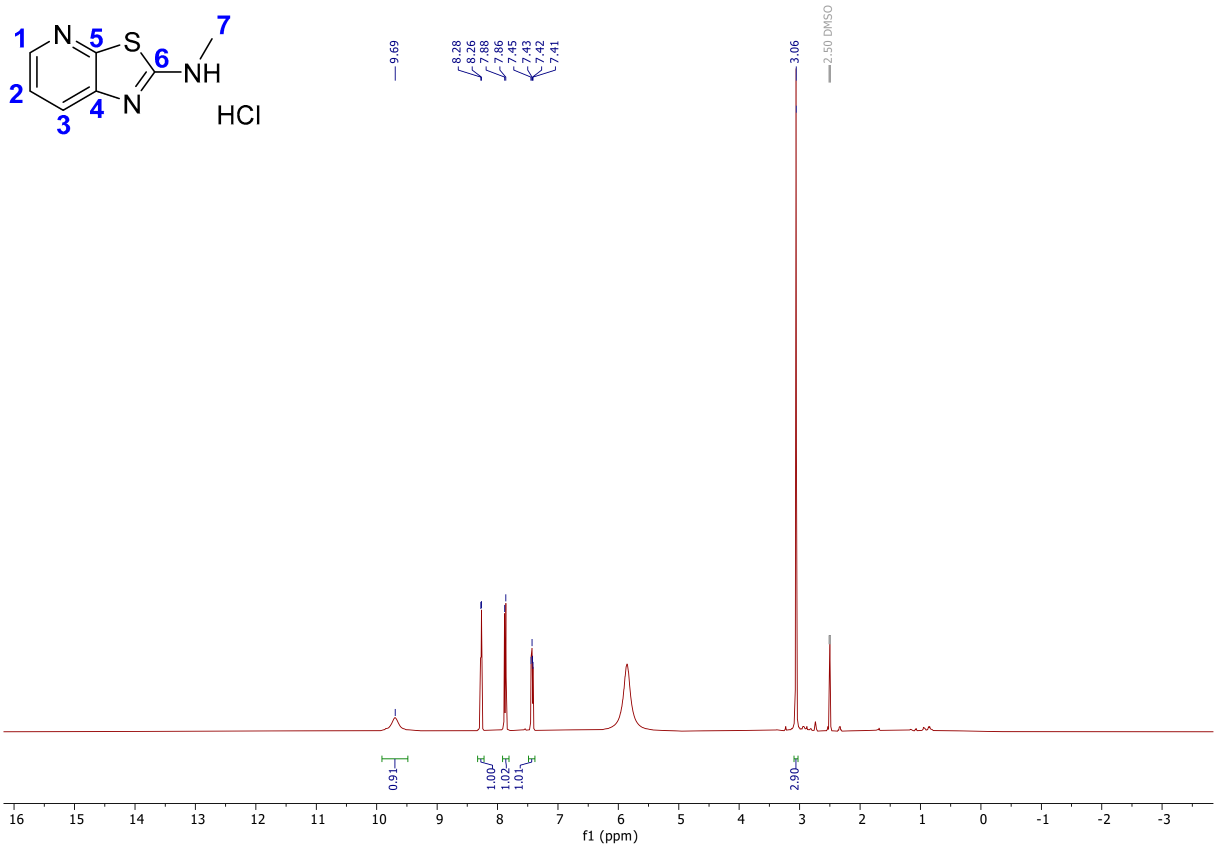

1
2
3
4
5
6
7
N
S
NH
HCl
9.69
8.28
8.26
7.88
7.86
7.45
7.43
7.42
7.41
3.06
2.50 DMSO
0.91
1.00
1.02
1.01
2.90
16 15 14 13 12 11 10 9 8 7 6 5 4 3 2 1 0 -1 -2 -3
f1 (ppm)

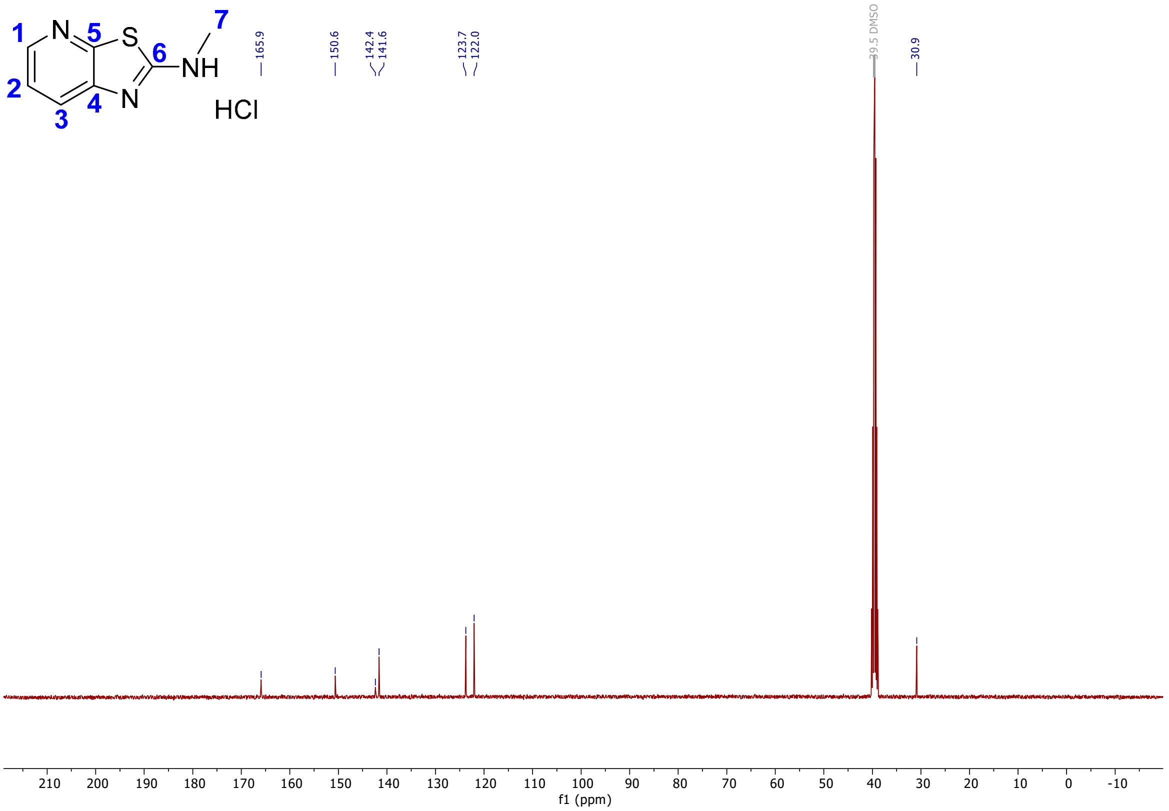

1
2
3
4
5
6
7
N
S
NH
HCl
165.9
150.6
142.4
141.6
123.7
122.0
39.5 DMSO
30.9
210 200 190 180 170 160 150 140 130 120 110 100 90 80 70 60 50 40 30 20 10 0 -10
f1 (ppm)

molecules MDPI

N-benzamidethiazolo[5,4-*b*]pyridin-2-amine hydrochloride (**4k**)

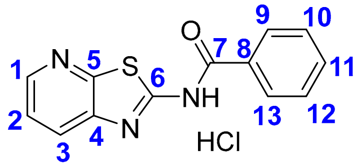

General procedure 1 was applied to benzoyl isothiocyanate and 3-amino-2-chloropyridine **2a** to give *N*-benzamidethiazolo[5,4-*b*]pyridin-2-amine hydrochloride **4k** in 46% yield.

Aspect : Beige powder

Melting point : 183°C.

HRMS (ESI+): m/z calculated for $C_{13}H_{10}N_3OS$ [M + H$^+$] = 256.0539 ; found = 256.0541.

^{1}H NMR (DMSO-*d6*, 400 MHz) : δ_H = 7.54 (dd, J = 8.2, 4.6 Hz, 1H, ^{2}H$_{Ar}$), 7.58 (t, J = 7.6 Hz, 2H, ^{10}H$_{Ar}$ and $^{10'}$H$_{Ar}$), 7.67-7.71 (m, 1H, H$_{Ar}$), 8.11-8.20 (m, 3H, ^{9}H$_{Ar}$ + $^{9'}$H$_{Ar}$ + ^{3}H$_{Ar}$), 8.52 (dd, J = 4.8, 1.4 Hz, 1.0H, ^{1}H$_{Ar}$), 12.95 (bs, 1H, N-H).

^{13}C NMR (DMSO-*d6*, 101 MHz) : δ_C = 121.8 (^{2}CH$_{Ar}$), 127.6 (^{3}CH$_{Ar}$), 128.4 (^{9}CH$_{Ar}$ and $^{9'}$CH$_{Ar}$), 128.7 (^{10}CH$_{Ar}$ and $^{10'}$CH$_{Ar}$), 131.6 (^{8}C^{IV}), 133.1 (^{11}CH$_{Ar}$), 141.8 (^{4}C^{IV}), 145.4 (^{1}CH$_{Ar}$), 154.7 (^{5}C^{IV}), 158.4 (^{6}C^{IV}) and 166.3 (^{7}C^{IV}=O).

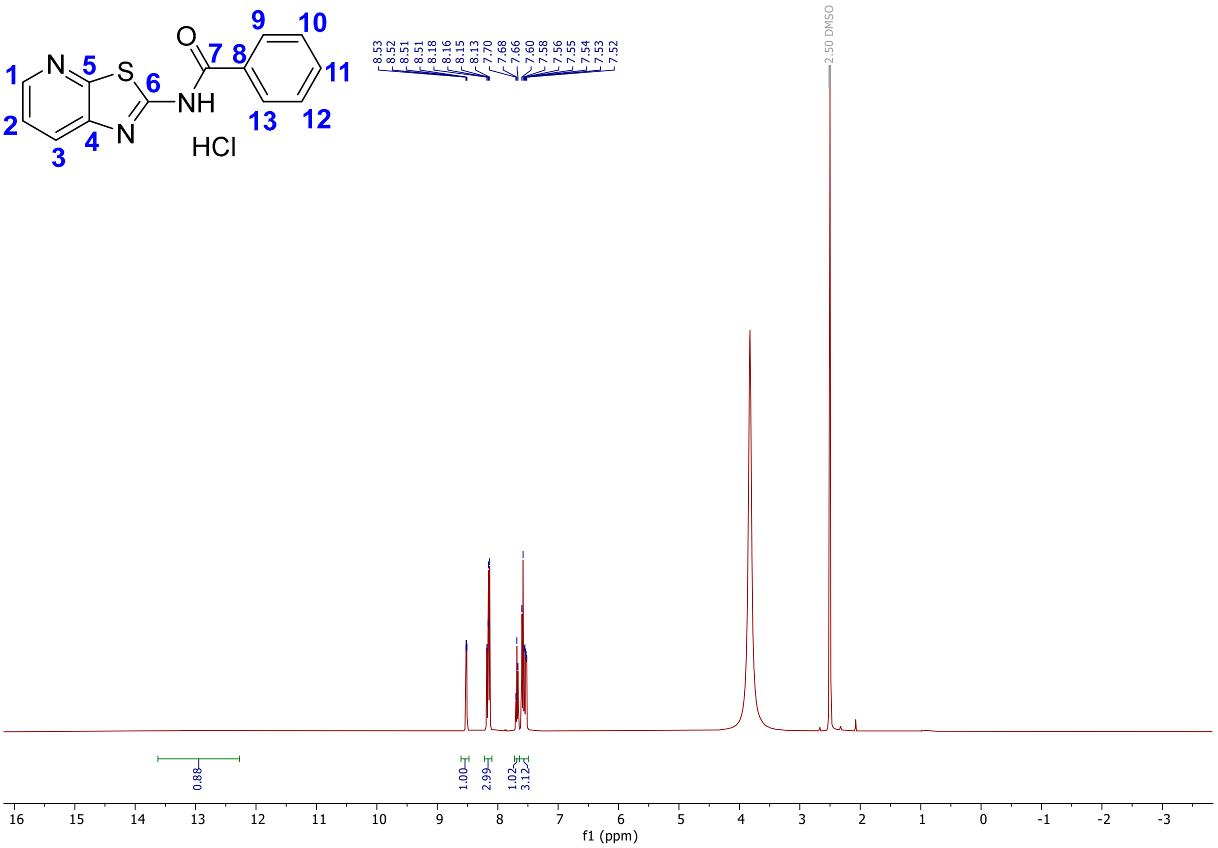
8.53
8.52
8.51
8.51
8.18
8.16
8.15
8.13
7.70
7.68
7.66
7.60
7.58
7.56
7.55
7.54
7.53
7.52
2.50 DMSO
HCl
0.88
1.00
2.99
1.02
3.12
f1 (ppm)

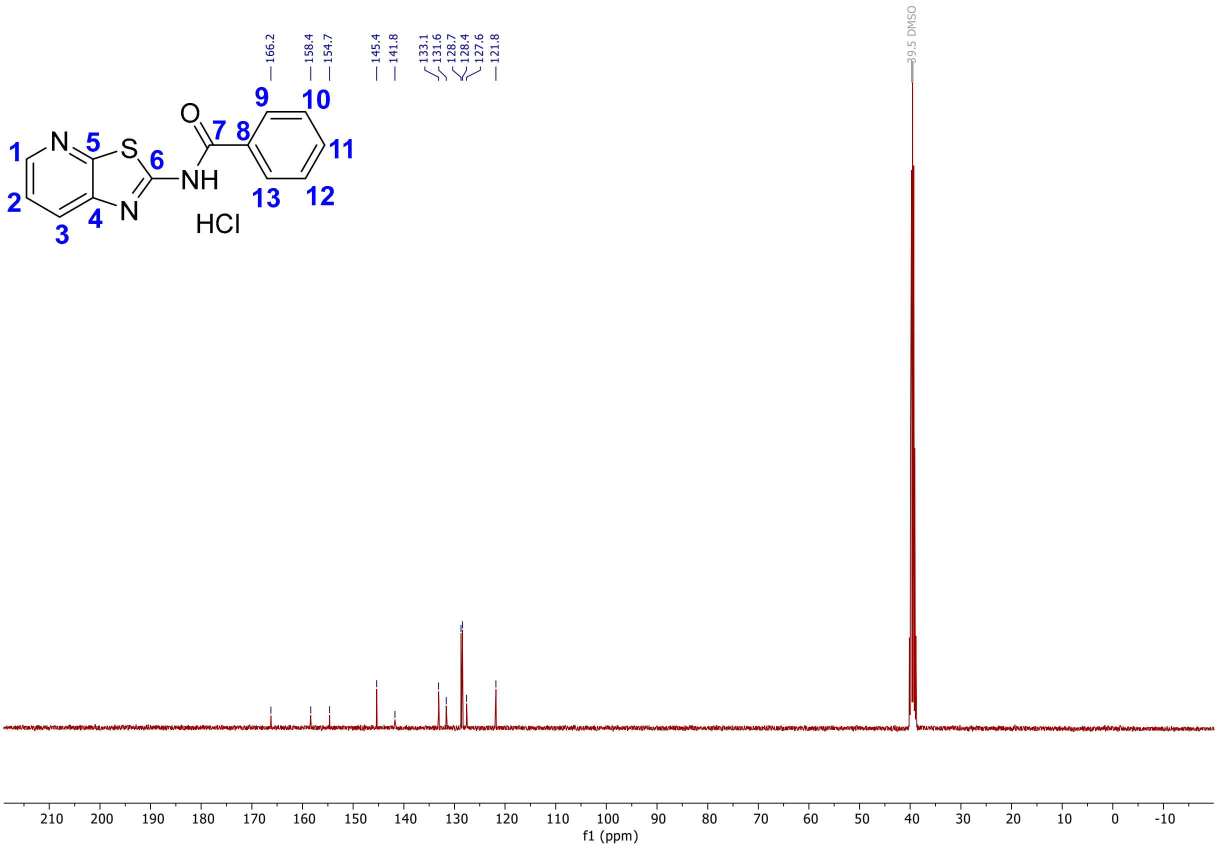
166.2
158.4
154.7
145.4
141.8
133.1
131.6
128.7
128.4
127.6
121.8
39.5 DMSO
HCl
f1 (ppm)

molecules

N-phenylthiazolo[5,4-*b*]-6-methylpyridin-2-amine hydrochloride (**5a**)

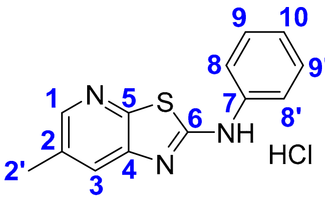

General procedure 2 was applied to phenyl isothiocyanate **3a** and 3-amino-2-chloro-5-methylpyridine **2b** to give *N*-phenylthiazolo[5,4-*b*]-6-methylpyridin-2-amine hydrochloride **5a** in 64% yield.

Aspect : beige powder

Melting point : 228°C (decomposition)

HRMS (ESI+): m/z calculated for $C_{13}H_{12}N_3S$ [M + H$^+$] = 242.0746 ; found = 242.0749

^{1}H NMR (DMSO-*d6*, 400 MHz) : δ_H = 2.38 (s, 3H, $^{2'}$CH$_3$), 7.06 (t, J = 7.4 Hz, 1H, ^{10}H$_{Ar}$), 7.38 (t, J = 7.6 Hz, 2H, ^{9}H$_{Ar}$ and $^{9'}$H$_{Ar}$), 7.80 (d, J = 8.3 Hz, 2H, ^{8}H$_{Ar}$ and $^{8'}$H$_{Ar}$), 7.88 (s, 1H, ^{3}H$_{Ar}$), 8.20 (s, 1H, ^{1}H$_{Ar}$), 10.93 (bs, 1H, N-H).

^{13}C NMR (DMSO-*d6*, 101 MHz) : δ_C = 17.8 ($^{2'}$CH$_3$), 118.5 (^{8}CH$_{Ar}$ and $^{8'}$CH$_{Ar}$), 122.9 (^{10}CH$_{Ar}$), 127.4 (^{3}CH$_{Ar}$), 129.1 (^{9}CH$_{Ar}$ and $^{9'}$CH$_{Ar}$), 131.7 (^{2}C^{IV}), 140.0 (^{7}C^{IV}), 141.2 (^{1}CH$_{Ar}$), 146.9 (^{4}C^{IV}), 149.4 (^{5}C^{IV}) and 161.5 (^{6}C^{IV}).

molecules

MDPI

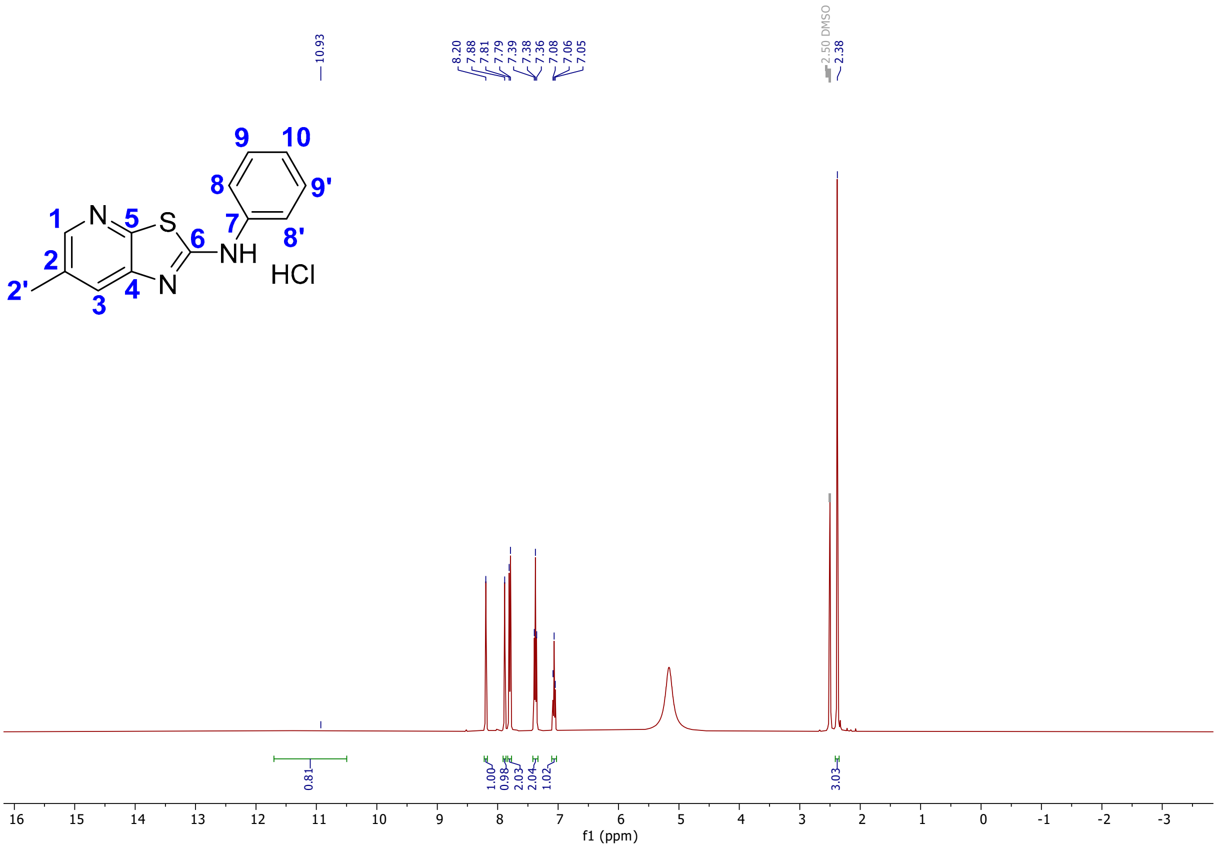

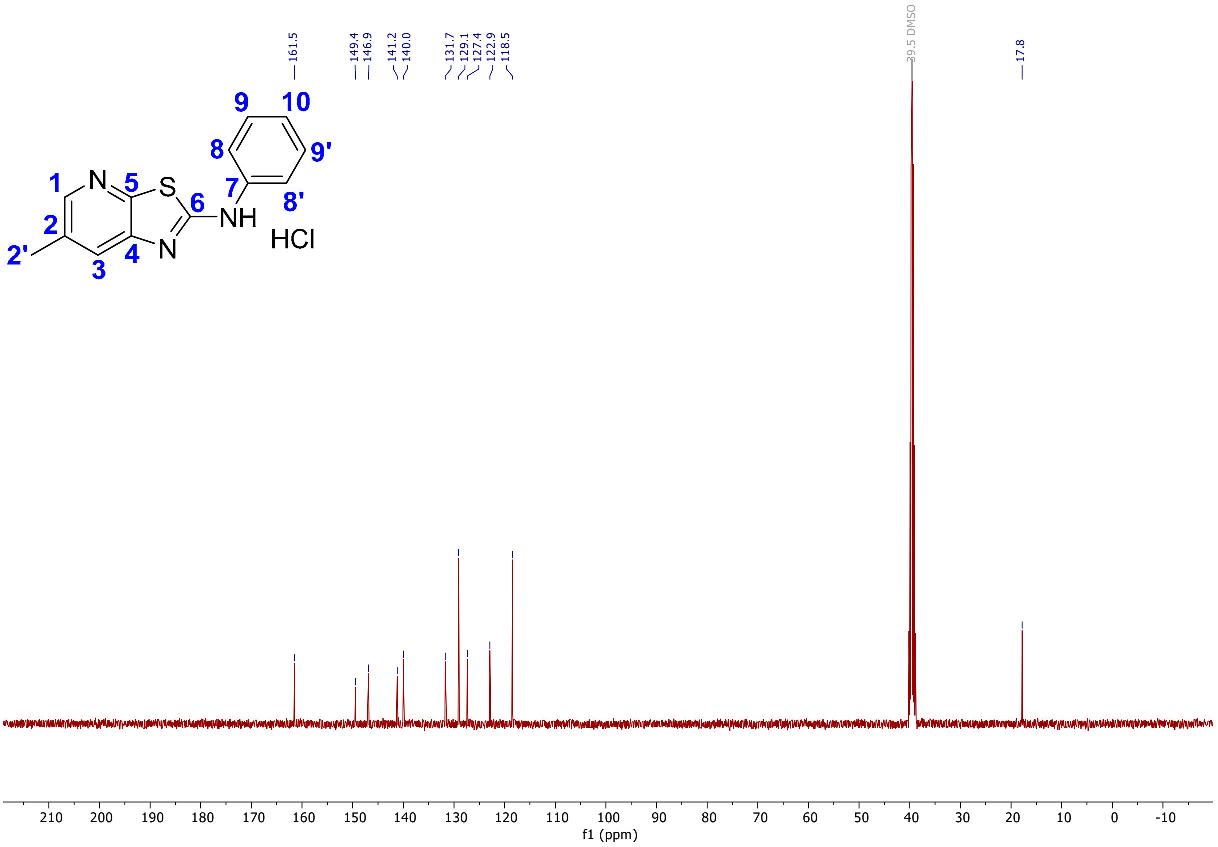

molecules

N-(3-bromophenyl)thiazolo[5,4-b]-6-methylpyridin-2-amine hydrochloride (**5b**)

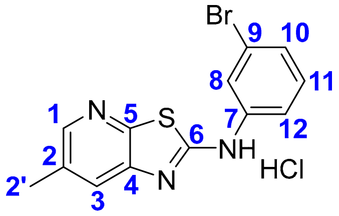

General procedure 1 was applied to 3-bromophenyl isothiocyanate and 3-amino-2-chloro-5-methylpyridine **2b** to give *N*-(3-bromophenyl)thiazolo[5,4-*b*]-6-methylpyridin-2-amine hydrochloride **5b** in 62% yield.

Aspect : beige powder.

Melting point : 247-248°C

HRMS (ESI+): m/z calculated for $C_{13}H_{11}BrN_3S$ [M + H$^+$] = 319.9852 ; found = 319.9847

^{1}H NMR (DMSO-*d6*, 400 MHz) : δ_H = 2.38 (s, 3H, $^{2'}$CH$_3$), 7.23 (d, J = 7.8 Hz, 1H, ^{12}H$_{Ar}$), 7.32 (t, J = 7.9 Hz, 1H, ^{11}H$_{Ar}$), 7.69 (d, J = 8.1 Hz, 1H, ^{10}H$_{Ar}$), 7.91 (s, 1H, ^{3}H$_{Ar}$), 8.20 (s, 2H, ^{1}H$_{Ar}$ and ^{8}H$_{Ar}$), 11.26 (bs, 1H, N-H).

^{13}C NMR (DMSO-*d6*, 101 MHz) : δ_C = 17.8 ($^{2'}$CH$_3$), 117.0 (^{10}CH$_{Ar}$), 120.4 (^{8}CH$_{Ar}$), 121.8 (^{9}C^{IV}), 125.1 (^{12}CH$_{Ar}$), 127.3 (^{3}CH$_{Ar}$), 130.9 (^{11}CH$_{Ar}$), 131.6 (^{2}C^{IV}), 141.5 (^{7}C^{IV}), 142.5 (^{1}CH$_{Ar}$), 146.2 (^{4}C^{IV}), 150.1 (^{5}C^{IV}) and 160.9 (^{6}C^{IV}).

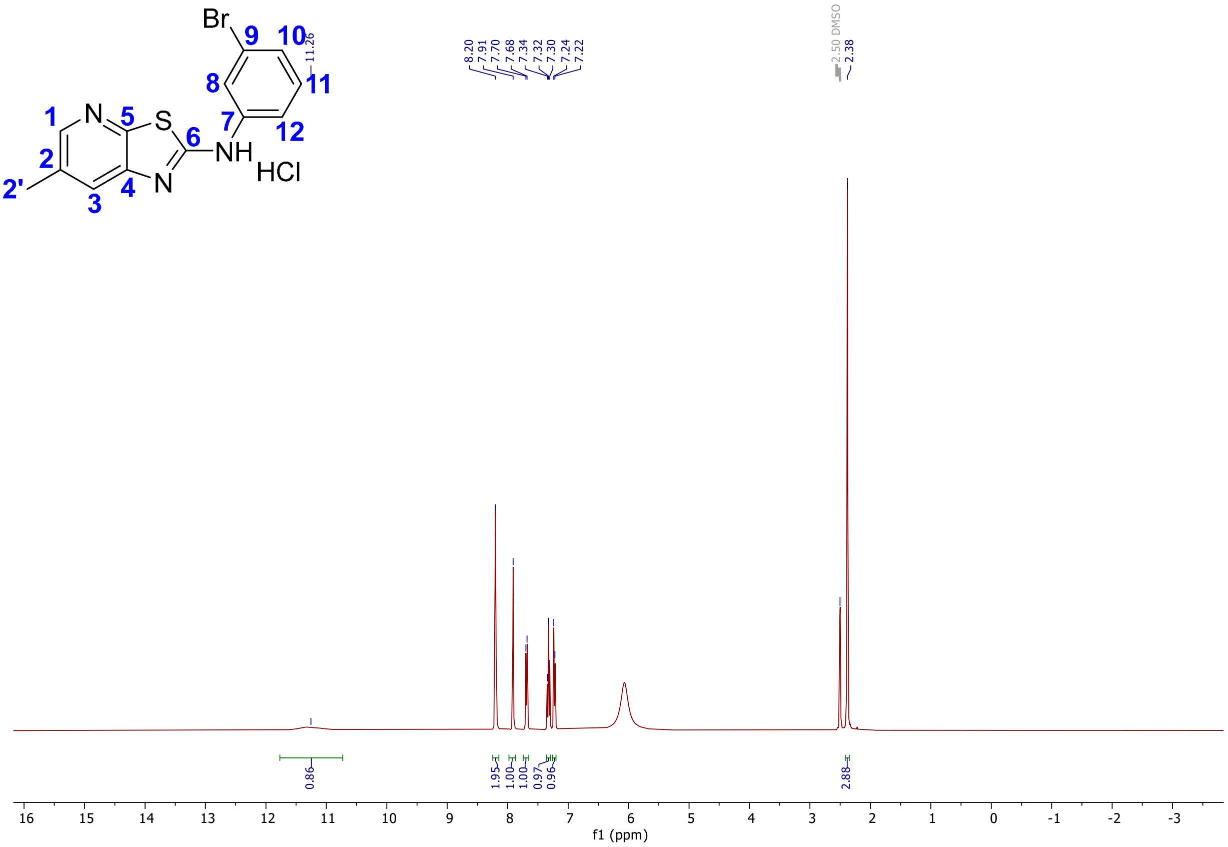
Br
9
10
8
11
1
N
5
S
6
7
NH
12
2
HCl
2'
4
N
3
11.26
8.20
7.91
7.70
7.68
7.34
7.32
7.30
7.24
7.22
2.50 DMSO
2.38
0.86
1.95
1.00
1.00
0.97
0.96
2.88
16 15 14 13 12 11 10 9 8 7 6 5 4 3 2 1 0 -1 -2 -3
f1 (ppm)

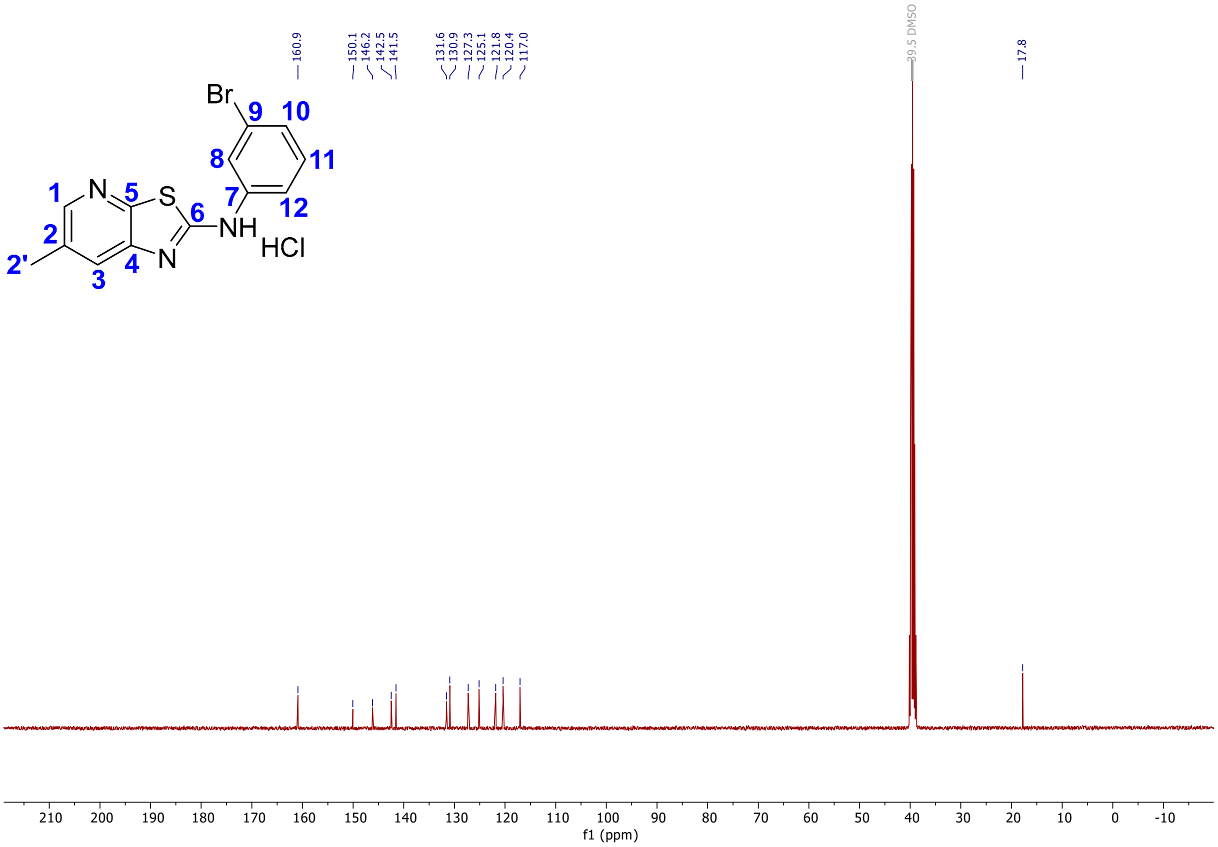
160.9
150.1
146.2
142.5
141.5
131.6
130.9
127.3
125.1
121.8
120.4
117.0
39.5 DMSO
17.8
Br
9
10
8
11
1
N
5
S
6
7
NH
12
2
HCl
2'
4
N
3
210 200 190 180 170 160 150 140 130 120 110 100 90 80 70 60 50 40 30 20 10 0 -10
f1 (ppm)

molecules

N-(3,5-bis(trifluoromethyl)phenyl)thiazolo[5,4-*b*]-6-methylpyridin-2-amine hydrochloride (**5c**)

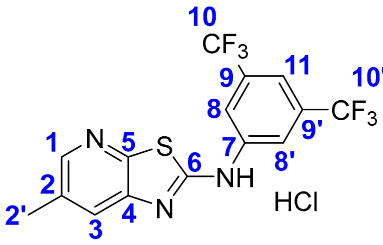

General procedure 2 was applied to 3,5-Bis(trifluoromethyl)phenyl isothiocyanate and 3-amino-2-chloro-5-methylpyridine **2b** to give *N*-(3,5-bis(trifluoromethyl)phenyl)thiazolo[5,4-*b*]-6-methylpyridin-2-amine hydrochloride **5c** in 48% yield.

Aspect : beige powder

Melting point : 242°C (decomposition)

HRMS (ESI+): m/z calculated for $C_{15}H_{10}F_6N_3S$ [M + H^+] = 378.0494 ; found = 378.0491

^{1}H NMR (DMSO-*d6*, 400 MHz) : δ_H = 2.37 (s, 3H, $^{2'}CH_3$), 7.67 (s, 1H, $^{11}H_{Ar}$), 7.89 (s, 1H, $^3H_{Ar}$), 8.19 (s, 1H, $^1H_{Ar}$), 8.49 (s, 2H, $^8CH_{Ar}$ and $^{8'}CH_{Ar}$), 11.83 (bs, 1H, N-H).

^{13}C NMR (DMSO-*d6*, 101 MHz) : δ_C = 17.7 ($^{2'}CH_3$), 114.8 ($^{11}CH_{Ar}$), 117.6 ($^8CH_{Ar}$ and $^{8'}CH_{Ar}$), 123.3 (q, ^1J = 274 Hz, $^{10}CF_3$ and $^{10'}CF_3$), 127.3 ($^3CH_{Ar}$), 130.9 (q, ^2J = 33 Hz, $^9C^{IV}$ and $^{9'}C^{IV}$), 131.6 ($^2C^{IV}$), 141.8 ($^7C^{IV}$), 143.9 ($^1CH_{Ar}$), 145.3 ($^4C^{IV}$), 150.6 ($^5C^{IV}$) and 160.6 ($^6C^{IV}$).

^{19}F NMR (DMSO-*d6*, 376 MHz) : δ_F = -61.68.

molecules MDPI

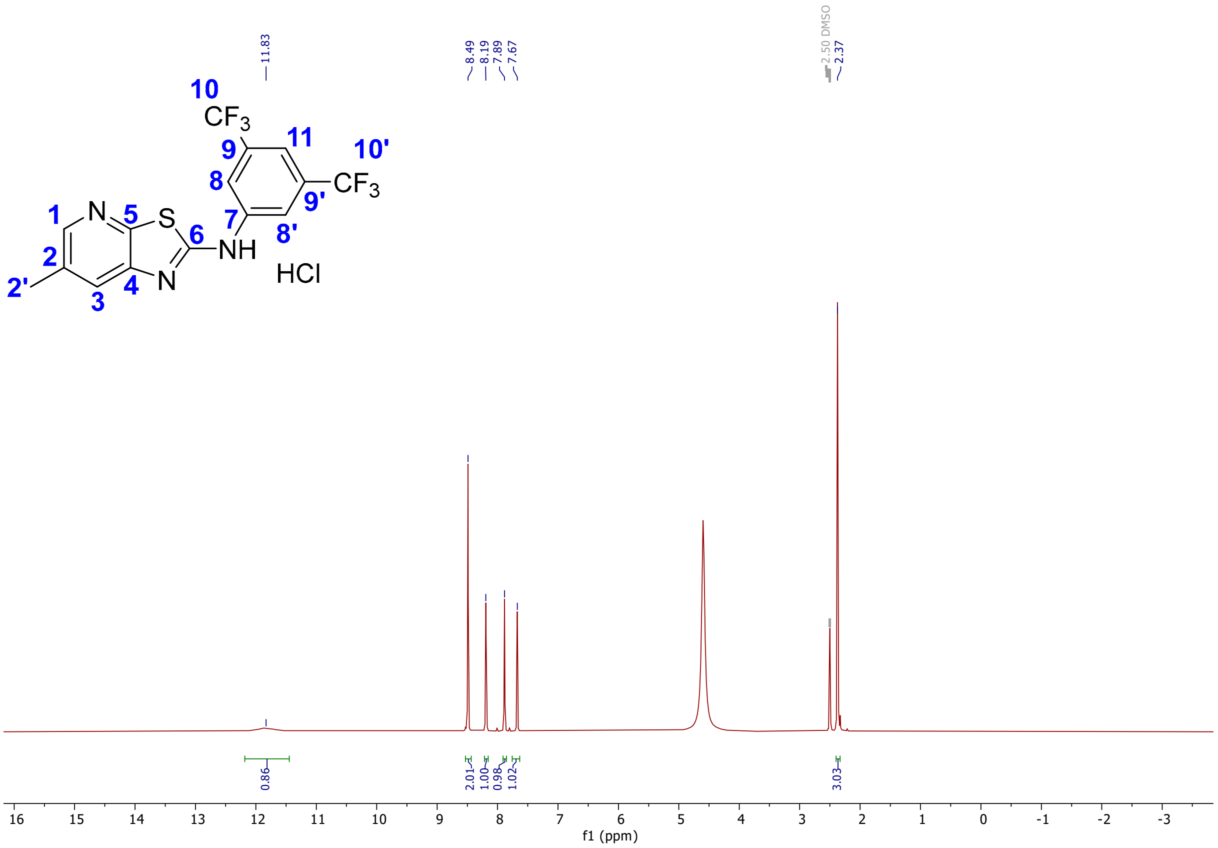

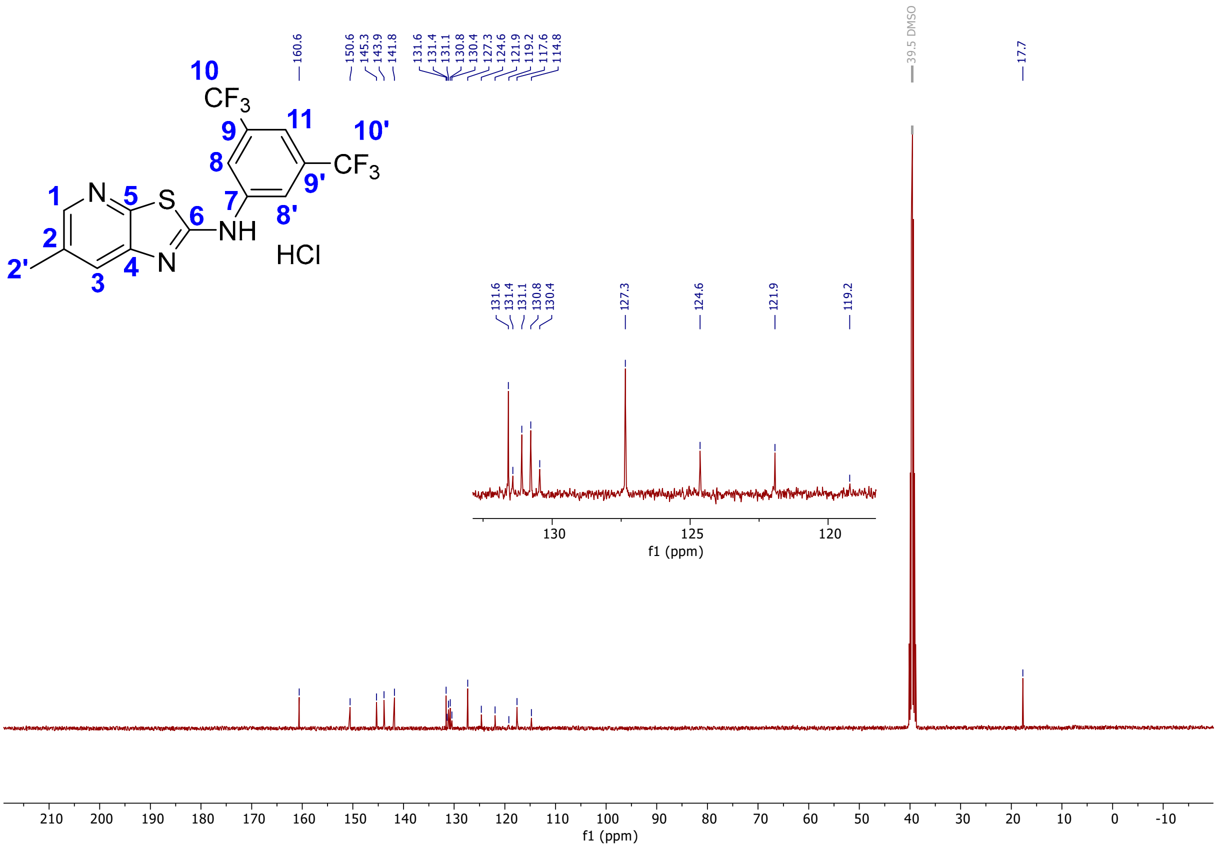

molecules MDPI

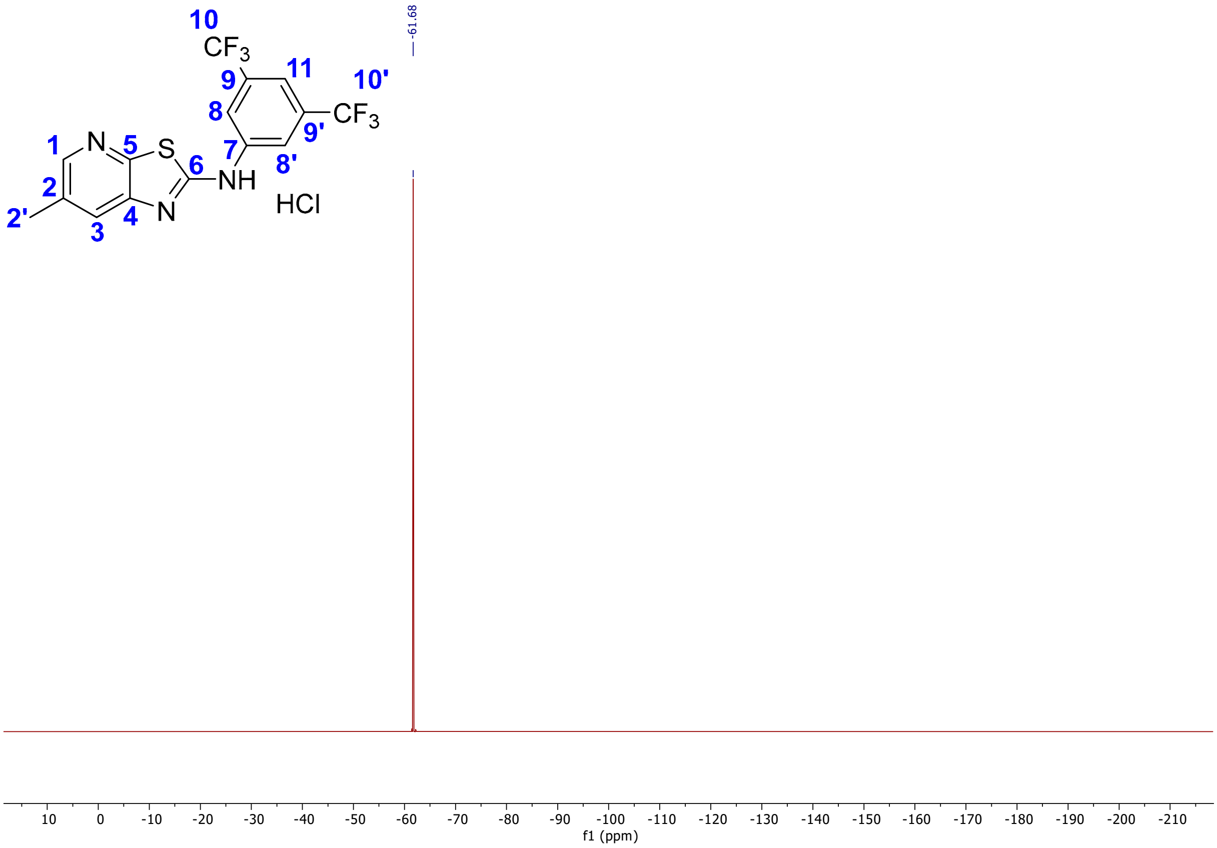

N-(4-methoxyphenyl)thiazolo[5,4-*b*]-6-methylpyridin-2-amine hydrochloride (**5d**)

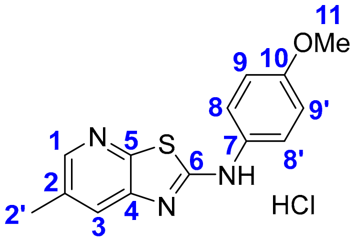

General procedure 2 was applied to 4-methoxyphenyl isothiocyanate and 3-amino-2-chloro-5-methylpyridine **2b** to give *N*-(4-methoxyphenyl)thiazolo[5,4-*b*]-6-methylpyridin-2-amine hydrochloride **5d** in 42% yield.

Aspect : yellow powder

Melting point : 212°C (decomposition)

HRMS (ESI+): m/z calculated for $C_{14}H_{14}N_3OS$ [M + H^+] = 272.0852 ; found = 272.0856

^{1}H NMR (DMSO-*d6*, 400 MHz) : δ_H = 2.37 (s, 3H, $^{2'}CH_3$), 3.75 (s, 3H, $^{11}CH_3$-O), 6.96 (d, J = 9.0 Hz, 2H, $^8H_{Ar}$ and $^{8'}H_{Ar}$), 7.67 (d, J = 9.0 Hz, 2H, $^9H_{Ar}$ and $^{9'}H_{Ar}$), 7.84 (s, 1H, $^3H_{Ar}$), 8.17 (s, 1H, $^1H_{Ar}$), 10.80 (bs, 1H, N-H).

^{13}C NMR (DMSO-*d6*, 101 MHz) : δ_C = 17.8 ($^{2'}CH_3$), 55.3 ($^{11}CH_3$-O), 114.3 ($^8CH_{Ar}$ and $^{8'}CH_{Ar}$), 120.6 ($^9CH_{Ar}$ and $^{9'}CH_{Ar}$), 127.0 ($^3CH_{Ar}$), 131.8 ($^2C^{IV}$), 133.1 ($^7C^{IV}$), 140.5 ($^1CH_{Ar}$), 147.0 ($^4C^{IV}$), 149.1 ($^5C^{IV}$), 155.4 ($^{10}C^{IV}$) and 162.1 ($^6C^{IV}$).

molecules

MDPI

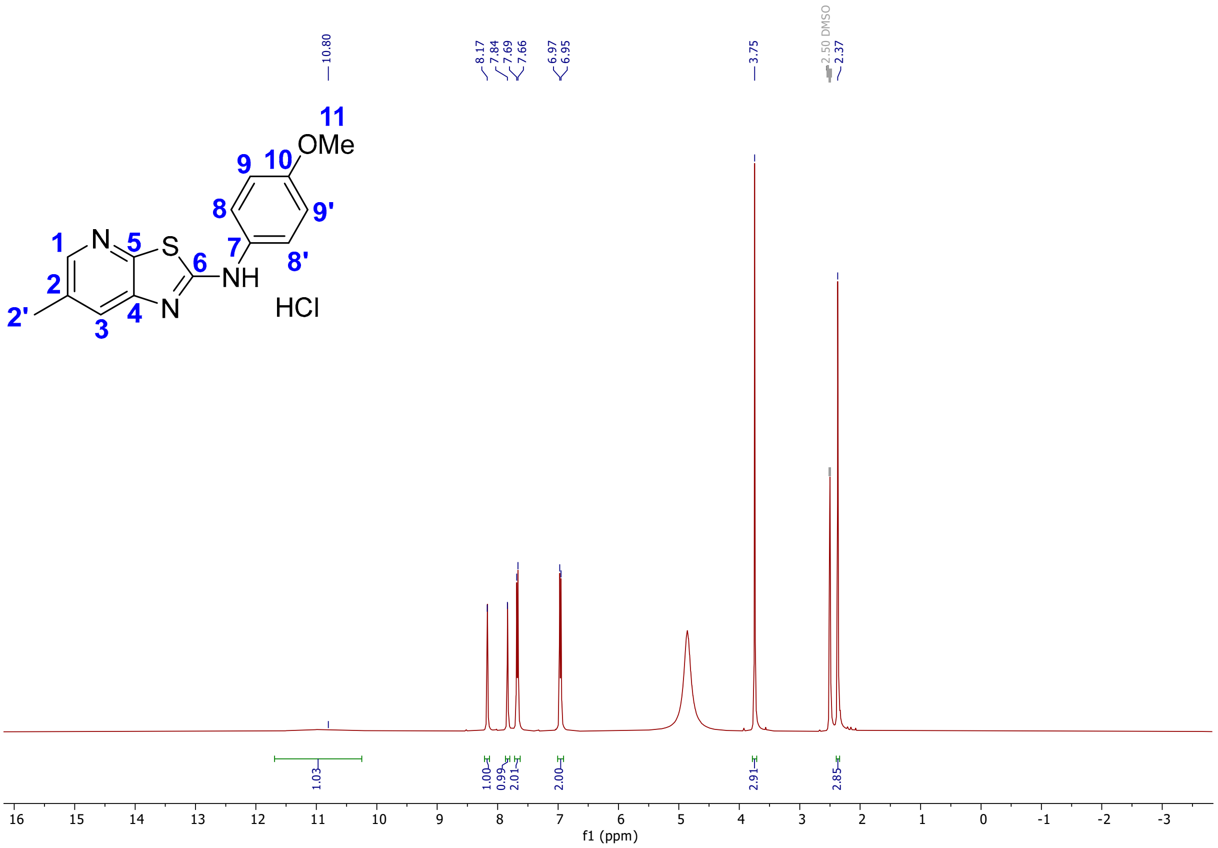

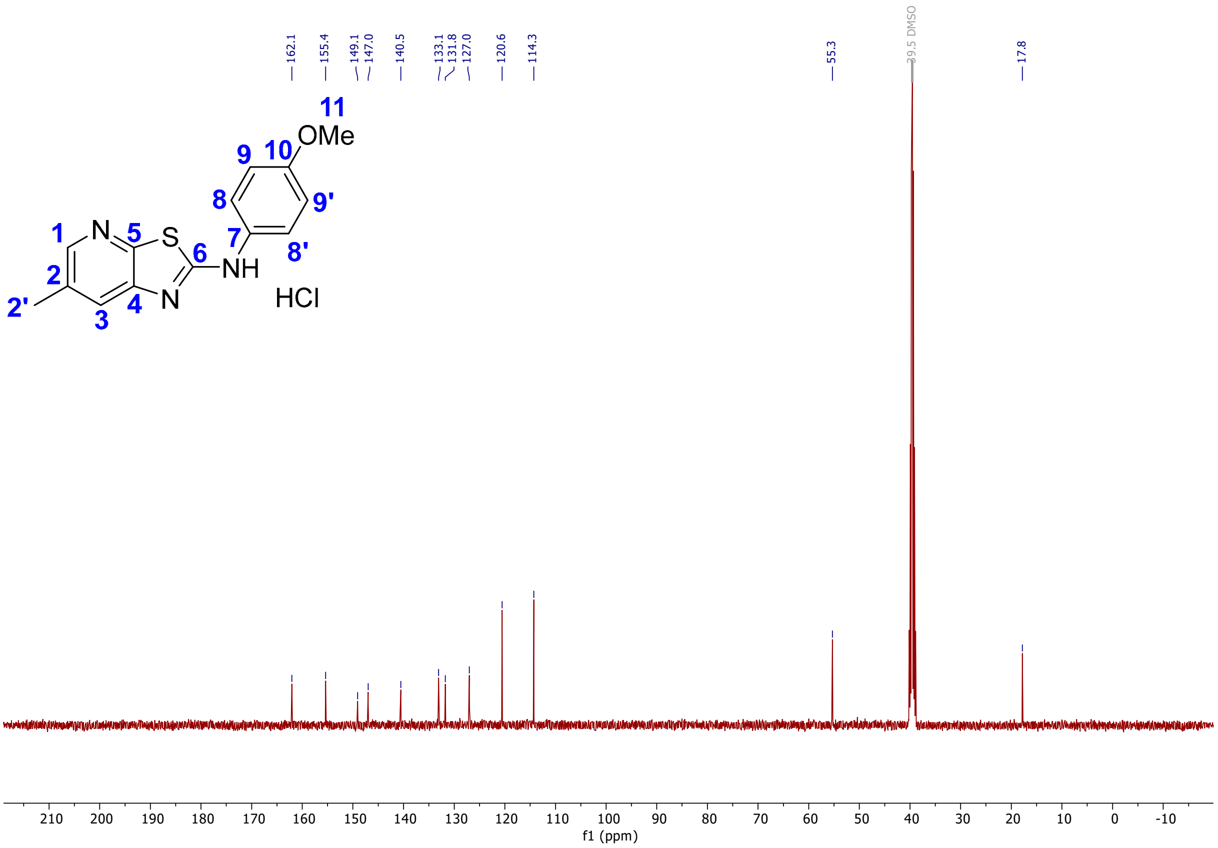

molecules MDPI

N-(3,5-dichlorophenyl)thiazolo[5,4-*b*]-6-methylpyridin-2-amine hydrochloride (**5e**)

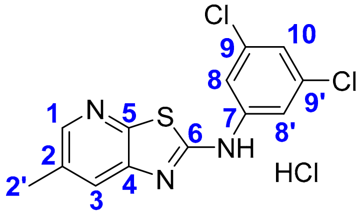

General procedure 2 was applied to 3,5-dichlorophenyl isothiocyanate and 3-amino-2-chloro-5-methylpyridine **2b** to give *N*-(3,5-dichlorophenyl)thiazolo[5,4-*b*]-6-methylpyridin-2-amine hydrochloride **5e** in 59% yield.

Aspect : pinkish powder

Melting point : 251°C

HRMS (ESI+): m/z calculated for $C_{13}H_{10}Cl_2N_3S$ [M + H^+] = 309.9967 ; found = 309.9973

^{1}H NMR (DMSO-*d6*, 250 MHz) : δ_H = 2.37 (s, 3H, $^{2'}$CH$_3$), 7.21 (t, J = 1.9 Hz, 1H, ^{10}H$_{Ar}$), 7.89 (d, J = 1.9 Hz, 2H, ^{8}H$_{Ar}$ and $^{8'}$H$_{Ar}$), 7.93 (d, J = 1.0 Hz, 1H, ^{3}H$_{Ar}$), 8.20 (d, J = 1.2 Hz, 1H, ^{1}H$_{Ar}$), 11.51 (bs, 1H, N-H).

^{13}C NMR (DMSO-*d6*, 101 MHz) : δ_C = 17.7 ($^{2'}$CH$_3$), 116.1 (^{8}CH$_{Ar}$ and $^{8'}$CH$_{Ar}$), 121.4 (^{10}CH$_{Ar}$), 127.4 (^{3}CH$_{Ar}$), 131.6 (^{2}C^{IV}), 134.2 (^{9}C^{IV} and $^{9'}$C^{IV}), 142.2 (^{7}C^{IV}), 143.2 (^{1}CH$_{Ar}$), 145.7 (^{4}C^{IV}), 150.3 (^{5}C^{IV}) and 160.6 (^{6}C^{IV}).

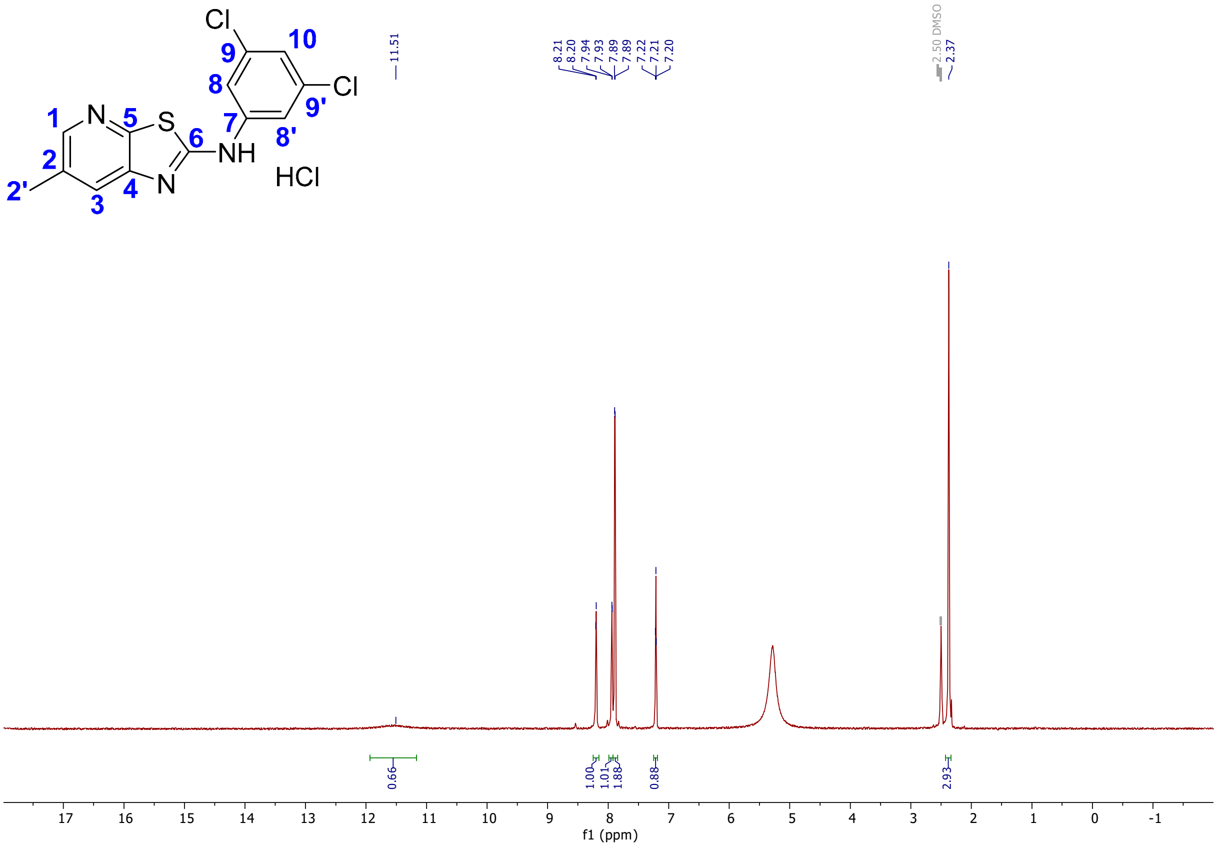
Cl
10
9
8
Cl
9'
1
N
5
S
6
7
NH
8'
2
2'
3
4
N
HCl
11.51
8.21
8.20
7.94
7.93
7.89
7.89
7.22
7.21
7.20
2.50 DMSO
2.37
0.66
1.00
1.01
1.88
0.88
2.93
17 16 15 14 13 12 11 10 9 8 7 6 5 4 3 2 1 0 -1
f1 (ppm)

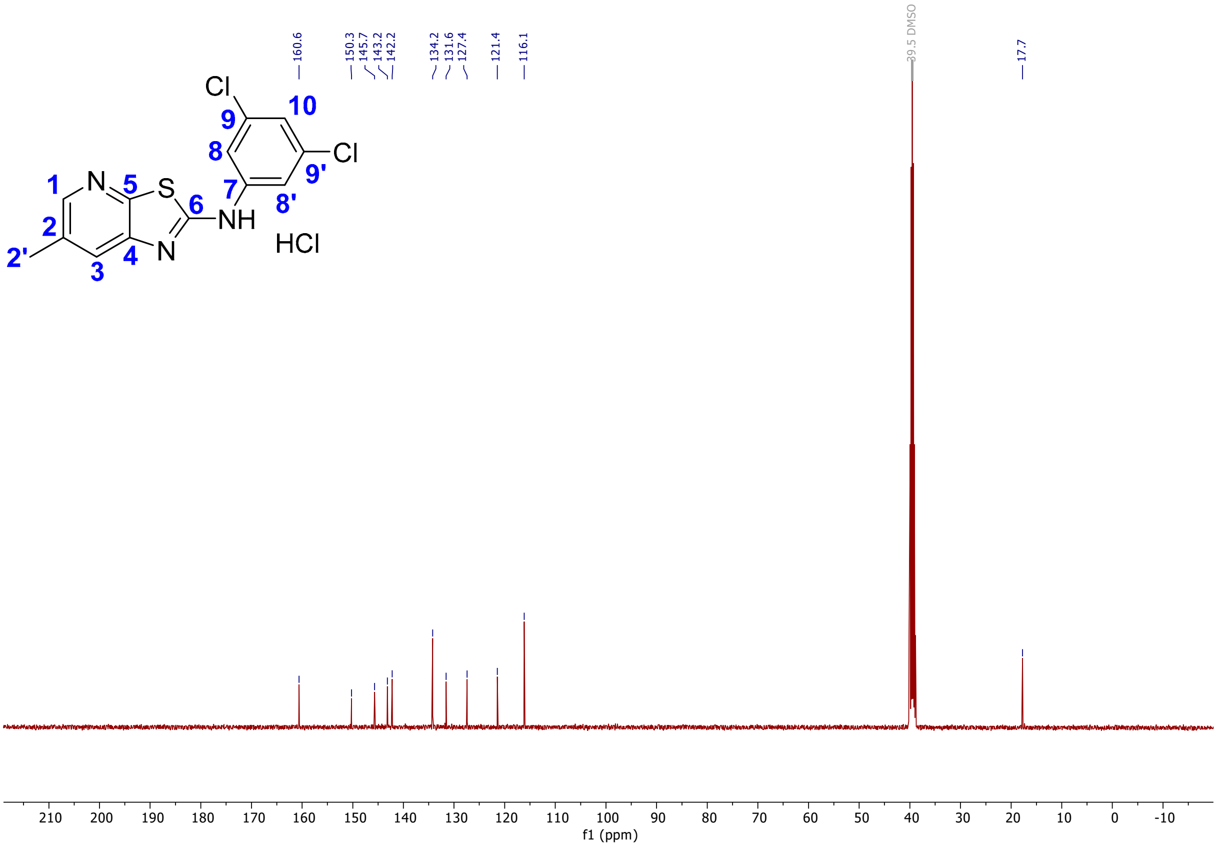
160.6
150.3
145.7
143.2
142.2
134.2
131.6
127.4
121.4
116.1
39.5 DMSO
17.7
Cl
10
9
8
Cl
9'
1
N
5
S
6
7
NH
8'
2
2'
3
4
N
HCl
210 200 190 180 170 160 150 140 130 120 110 100 90 80 70 60 50 40 30 20 10 0 -10
f1 (ppm)

molecules

N-(3-chlorophenyl)thiazolo[5,4-*b*]-6-methylpyridin-2-amine hydrochloride (**5f**)

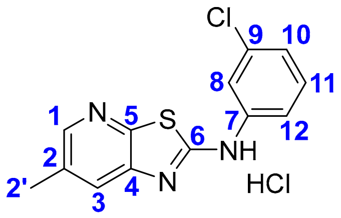

General procedure 1 was applied to 3-chlorophenyl isothiocyanate and 3-amino-2-chloro-5-methylpyridine **2b** to give *N*-(3-chlorophenyl)thiazolo[5,4-*b*]-6-methylpyridin-2-amine hydrochloride **5f** in 67% yield.

Aspect : colourless powder

Melting point : 214°C.

HRMS (ESI+): m/z calculated for $C_{13}H_{11}ClN_3S$ [M + H$^+$] = 276.0357 ; found = 267.0360.

^{1}H NMR (DMSO-*d6*, 400 MHz) : δ_H = 2.38 (s, 3H, $^{2'}$CH$_3$), 7.09 (dd, J = 8.0, 2.3 Hz, 1H, ^{10}H$_{Ar}$), 7.38 (t, J = 8.0 Hz, 1H, ^{11}H$_{Ar}$), 7.64 (dd, J = 8.2, 2.3 Hz, 1H, ^{12}H$_{Ar}$), 7.93 (s, 1H, ^{3}H$_{Ar}$), 8.08 (s, 1H, ^{8}H$_{Ar}$), 8.21 (s, 1H, ^{1}H$_{Ar}$), 11.34 (bs, 1H, N-H).

^{13}C NMR (DMSO-*d6*, 101 MHz) : δ_C = 17.8 ($^{2'}$CH$_3$), 116.7 (^{12}CH$_{Ar}$), 117.6 (^{8}CH$_{Ar}$), 122.2 (^{10}CH$_{Ar}$), 127.6 (^{3}CH$_{Ar}$), 130.6 (^{11}CH$_{Ar}$), 131.7 (^{2}C^{IV}), 133.3 (^{9}C^{IV}), 141.4 (^{7}C^{IV}), 141.9 (^{1}CH$_{Ar}$), 146.4 (^{4}C^{IV}), 149.7 (^{5}C^{IV}) and 161.0 (^{6}C^{IV}).

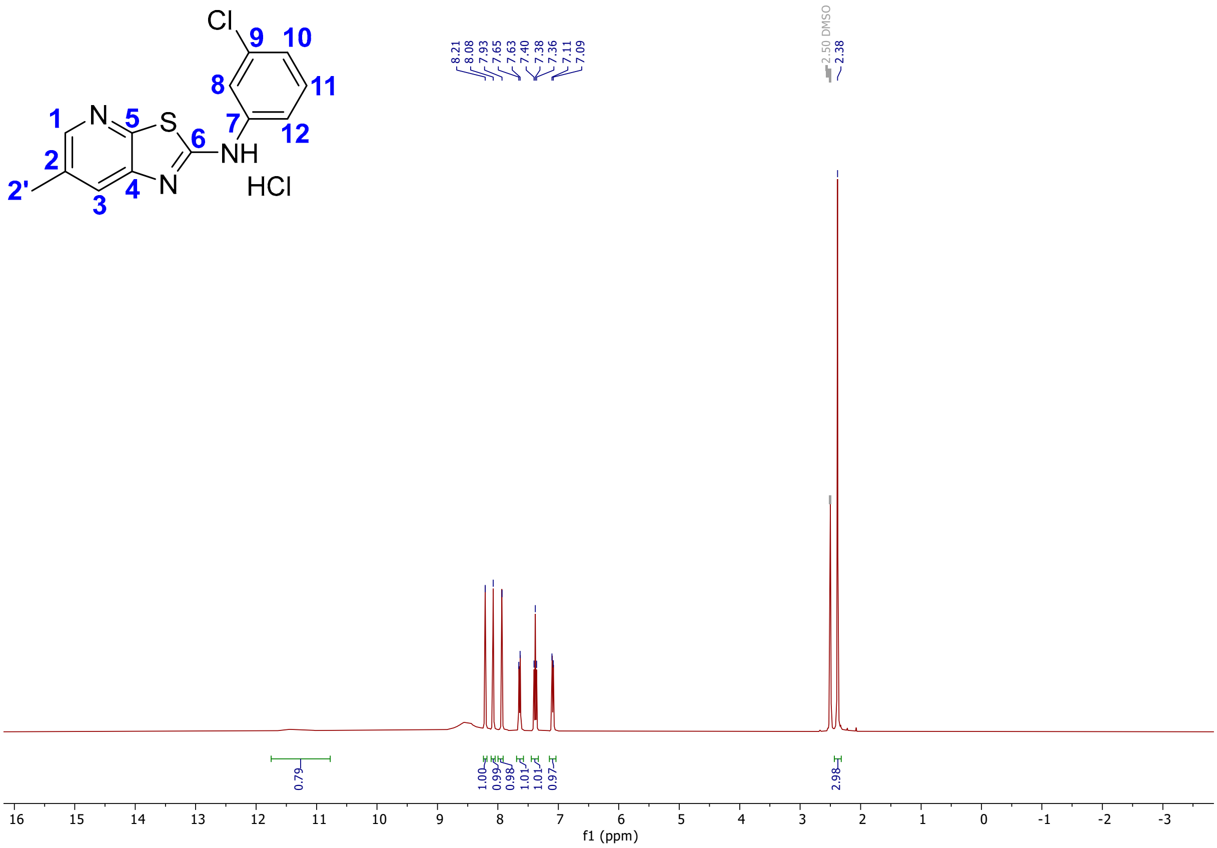
Cl
9 10
8 11
1 N 5 S 7
6 NH 12
2
2' 3 4 N HCl
8.21
8.08
7.93
7.65
7.63
7.40
7.38
7.36
7.11
7.09
2.50 DMSO
2.38
0.79
1.00
0.99
0.98
1.01
1.01
0.97
2.98
f1 (ppm)

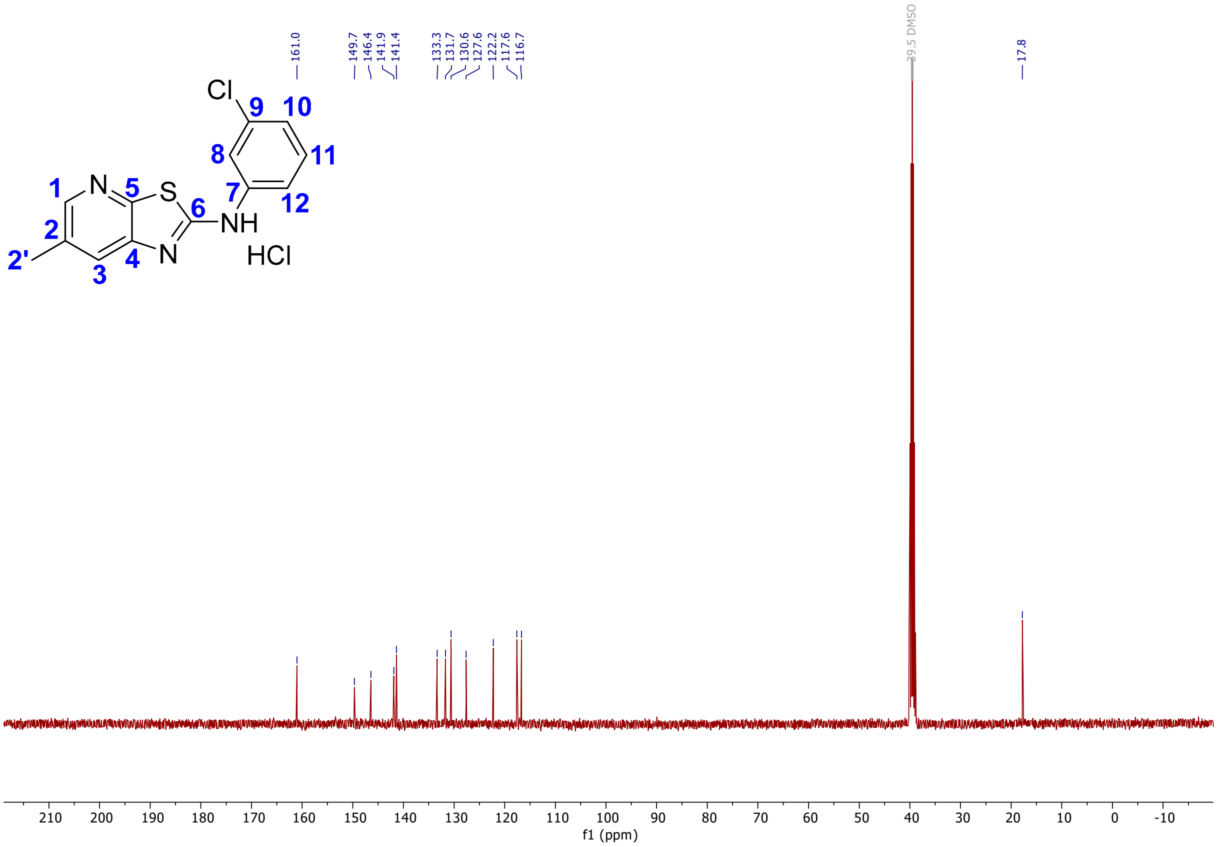
161.0
149.7
146.4
141.9
141.4
133.3
131.7
130.6
127.6
122.2
117.6
116.7
39.5 DMSO
17.8
Cl
9 10
8 11
1 N 5 S 7
6 NH 12
2
2' 3 4 N HCl
f1 (ppm)

molecules

MDPI

N-benzamidethiazolo[5,4-*b*]-6-methyl-2-amine hydrochloride (**5g**)

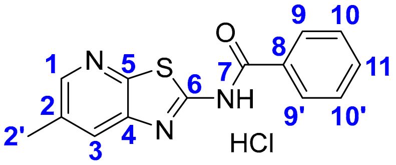

General procedure 1 was applied to benzoyl isothiocyanate and 3-amino-2-chloro-5-methylpyridine **2b** to give *N*-benzamidethiazolo[5,4-*b*]-6-methyl-2-amine hydrochloride **5g** in 46% yield.

Aspect : colourless powder

Melting point : 222°C

HRMS (ESI+): m/z calculated for $C_{14}H_{12}N_3OS$ [M + H$^+$] = 270.0696 ; found = 270.0698

^{1}**H NMR (DMSO-*d6*, 400 MHz) :** δ_H = 2.43 (s, 3H, $^{2'}$CH$_3$), 7.57 (t, J = 7.7 Hz, 2H, ^{10}H$_{Ar}$ and $^{10'}$H$_{Ar}$), 7.67 (t, J = 7.3 Hz, 1H, ^{11}H$_{Ar}$), 7.97 (s, 1H, ^{3}H$_{Ar}$), 8.13 (d, J = 7.8 Hz, 2H, ^{9}H$_{Ar}$ and $^{9'}$H$_{Ar}$), 8.35 (s, 1H, ^{1}H$_{Ar}$), 12.91 (bs, 1H, N-H).

^{13}**C NMR (DMSO-*d6*, 101 MHz) :** δ_C = 17.9 ($^{2'}$CH$_3$), 127.7 (^{3}CH$_{Ar}$), 128.4 (^{9}CH$_{Ar}$ and $^{9'}$CH$_{Ar}$), 128.7 (^{10}CH$_{Ar}$ and $^{10'}$CH$_{Ar}$), 131.5 (^{2}C^{IV}), 131.7 (^{8}C^{IV}), 133.1 (^{11}CH$_{Ar}$), 141.7 (^{4}C^{IV}), 146.2 (^{1}CH$_{Ar}$), 151.8 (^{5}C^{IV}), 158.6 (^{6}C^{IV}) and 166.2 (^{7}C^{IV}=O).

molecules

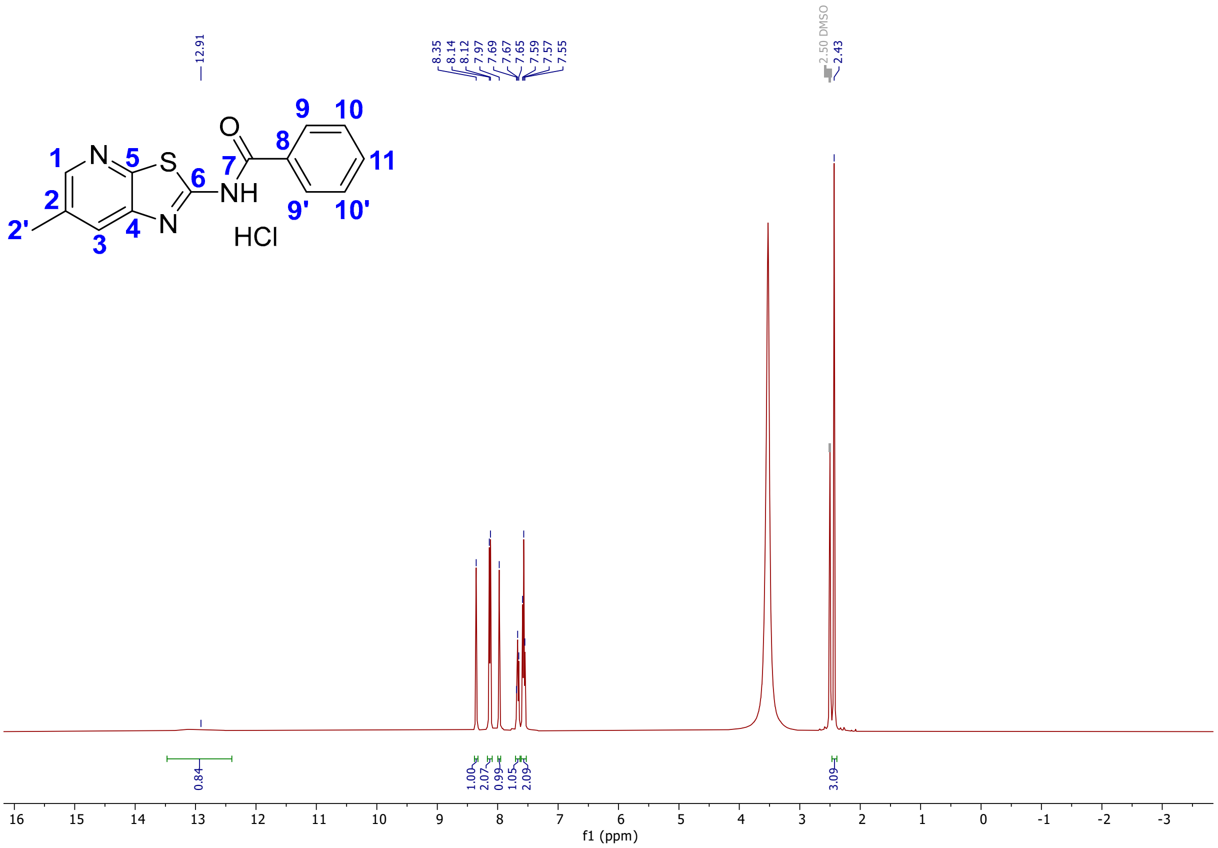
12.91
8.35
8.14
8.12
7.97
7.69
7.67
7.65
7.59
7.57
7.55
2.50 DMSO
2.43
0.84
1.00
2.07
0.99
1.05
2.09
3.09
NH
HCl
f1 (ppm)

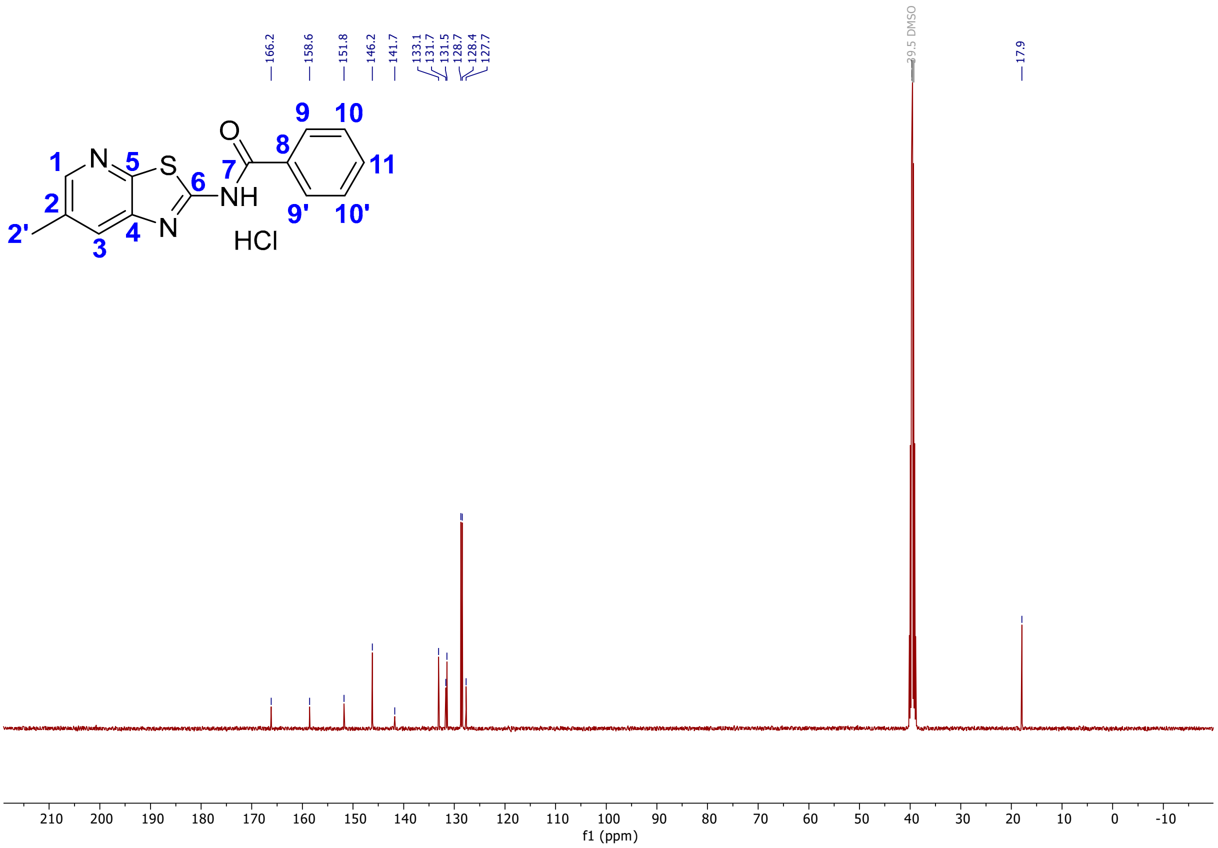
166.2
158.6
151.8
146.2
141.7
133.1
131.7
131.5
128.7
128.4
127.7
39.5 DMSO
17.9
NH
HCl
f1 (ppm)

molecules MDPI

N-(ethyl 4-aminobenzoate)thiazolo[5,4-*b*]-6-methylpyridin-2-amine hydrochloride (**5h**)

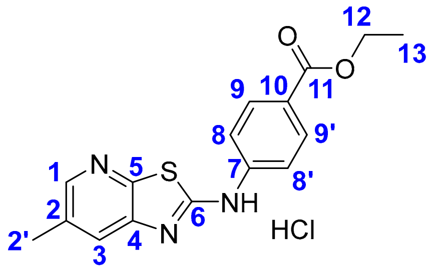

General procedure 1 was applied to ethyl 4-isothiocyanatobenzoate and 3-amino-2-chloro-5-methylpyridine **2b** to give *N*-(ethyl 4-aminobenzoate)thiazolo[5,4-*b*]-6-methylpyridin-2-amine hydrochloride **5h** in 64% yield.

Aspect : beige powder

Melting point : 210°C (decomposition).

HRMS (ESI+): m/z calculated for $C_{16}H_{16}N_3O_2S$ [M + H+] = 314.0958 ; found = 314.0956.

^{1}H NMR (DMSO-*d6*, 400 MHz) : δ_H = 1.31 (t, J = 7.0 Hz, 3H, $^{13}CH_3$), 2.38 (s, 3H, $^{2'}CH_3$), 4.28 (q, J = 7.0 Hz, 2H, $^{12}CH_2$), 7.89 (s, 1H, $^3H_{Ar}$), 7.89-7.98 (m, 4H, H_{Ar}), 8.20 (s, 1H, $^1H_{Ar}$), 11.41 (bs, 1H, N-H).

^{13}C NMR (DMSO-*d6*, 101 MHz) : δ_C = 14.2 ($^{13}CH_3$), 17.8 ($^{2'}CH_3$), 60.4 ($^{12}CH_2$), 117.5 (2 x CH_{Ar}), 123.4 ($^{10}C^{IV}$), 127.4 ($^3CH_{Ar}$), 130.5 (2 x CH_{Ar}), 131.6 ($^2C^{IV}$), 142.7 (^1CH), 144.2 ($^7C^{IV}$), 146.1 ($^4C^{IV}$), 150.2 ($^5C^{IV}$), 160.8 ($^6C^{IV}$) and 165.3 ($^{11}C^{IV}$).

molecules
MDPI

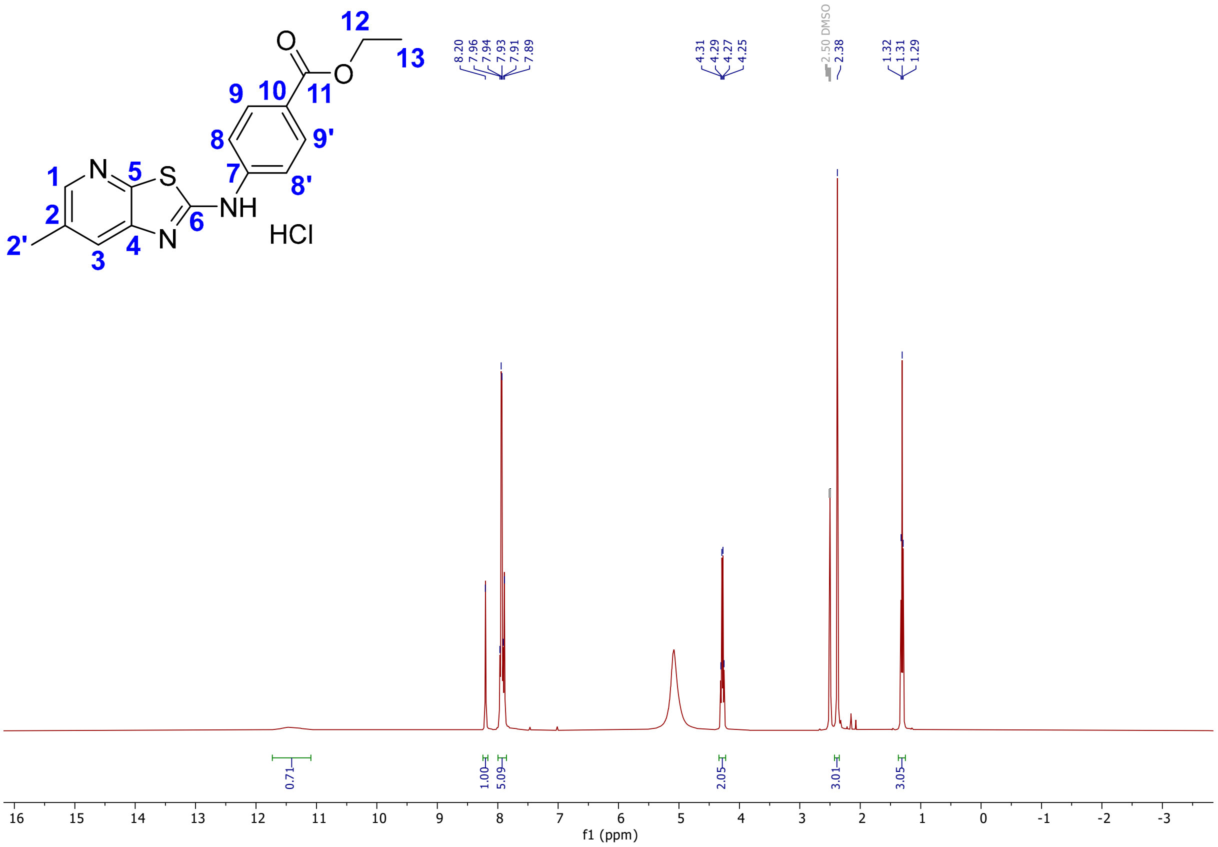

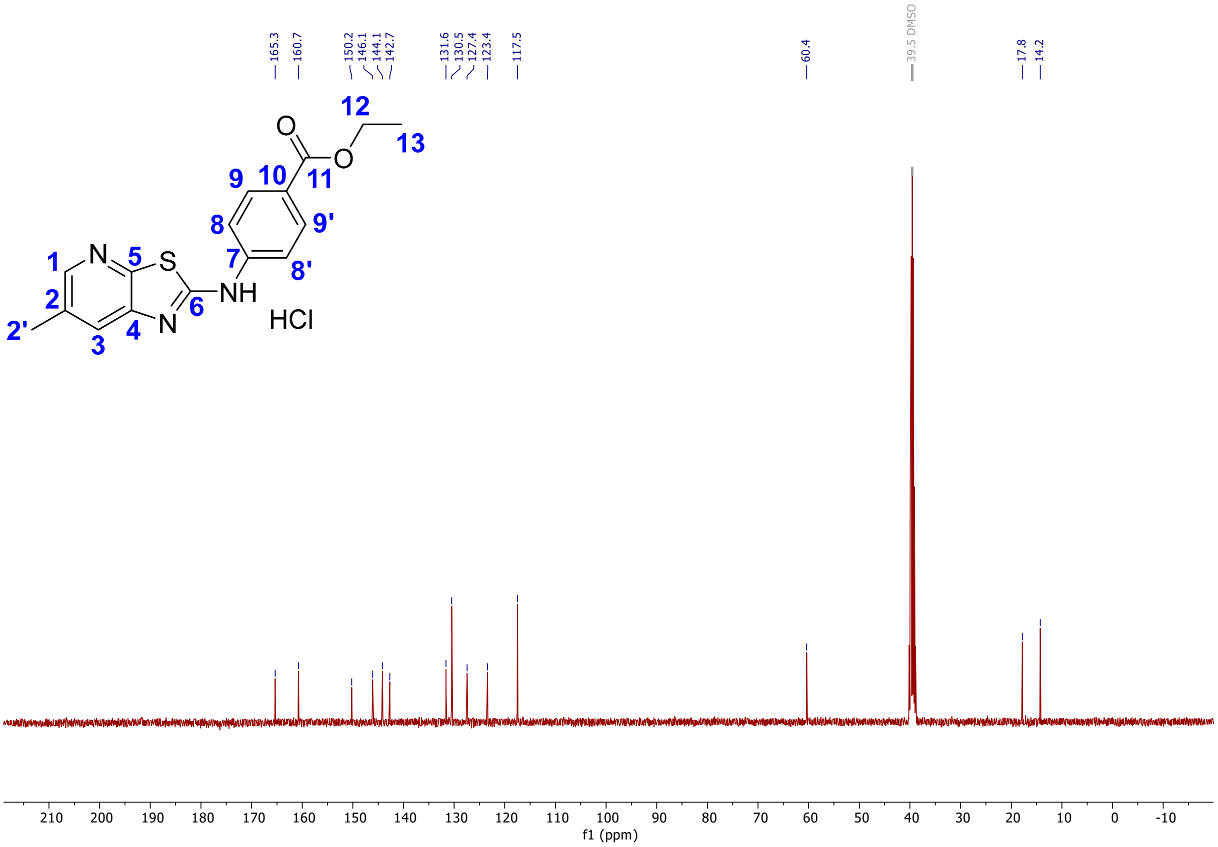

molecules

N-methylthiazolo[5,4-*b*]-6-methylpyridin-2-amine hydrochloride (**5i**)

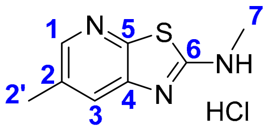

General procedure 1 was applied to methyl isothiocyanate and 3-amino-2-chloro-5-methylpyridine **2b** to give *N*-methylthiazolo[5,4-*b*]-6-methylpyridin-2-amine hydrochloride **5i** in 40% yield.

Aspect : colourless powder

Melting point : 186°C (decomposition)

HRMS (ESI+): m/z calculated for $C_8H_{10}N_3S$ [M + H^+] = 180.0590 ; found = 180.0587

^{1}H NMR (D_2O, 400 MHz) : δ_H = 2.39 (s, 3H, $^{2'}CH_3$), 3.13 (s, 3H, $^7CH_3$), 7.60 (s, 1H, $^3H_{Ar}$), 8.12 (s, 1H, $^1H_{Ar}$).

^{13}C NMR (D_2O, 101 MHz) : δ_C = 17.5 ($^{2'}CH_3$), 31.4 ($^7CH_3$), 124.3 ($^3CH_{Ar}$), 134.2 ($^2C^{IV}$), 138.1 (C^{IV}), 142.8 ($^1CH_{Ar}$), 143.3 (C^{IV}) and 167.7 ($^6C^{IV}$).

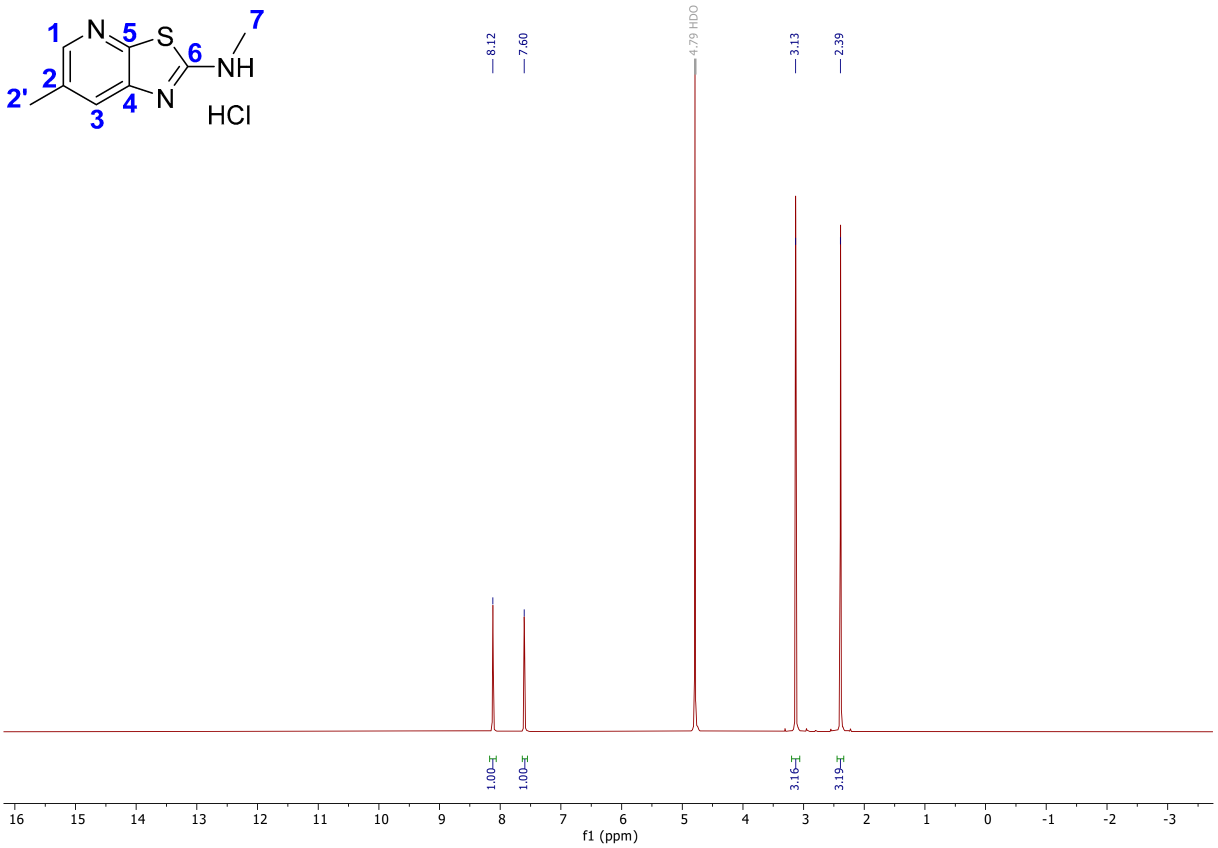
1
N
5
S
6
7
NH
2
2'
3
4
N
HCl
8.12
7.60
4.79 HDO
3.13
2.39
1.00
1.00
3.16
3.19
f1 (ppm)

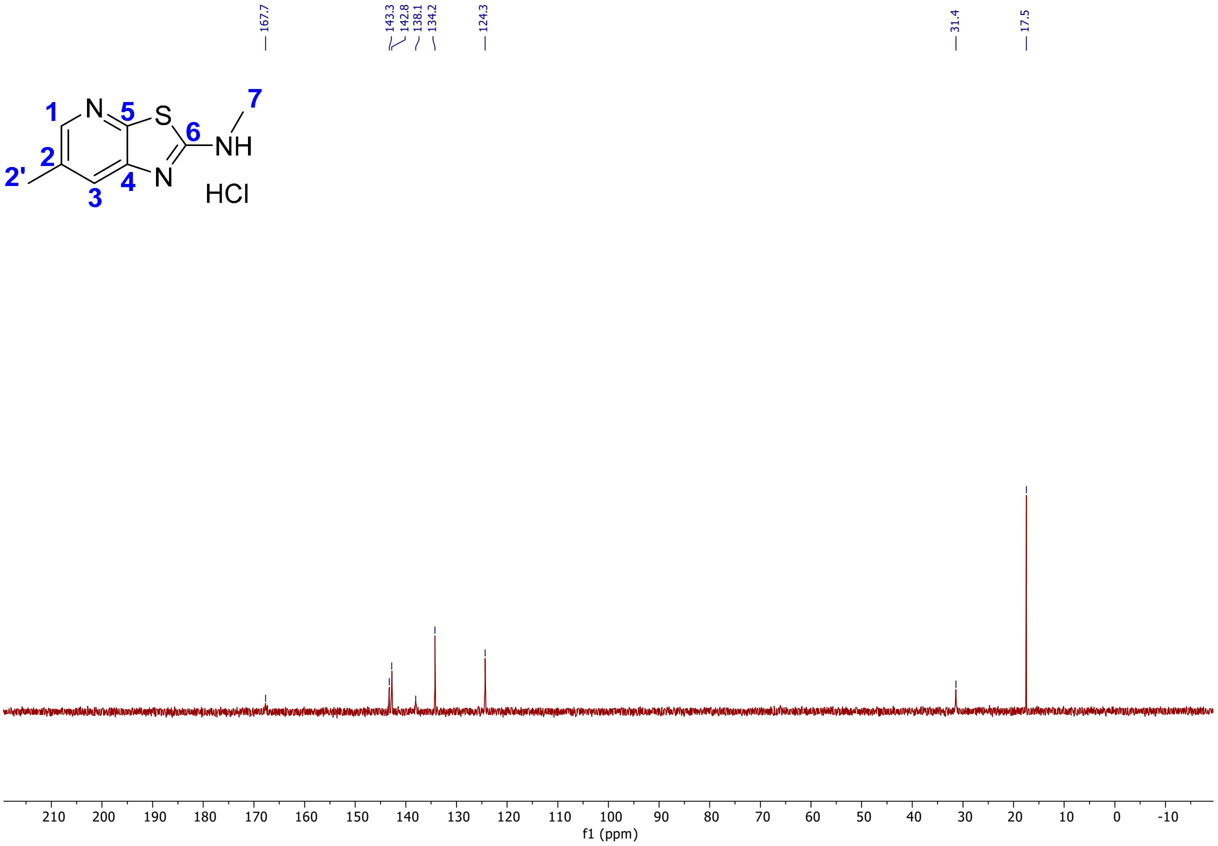
167.7
143.3
142.8
138.1
134.2
124.3
31.4
17.5
1
N
5
S
6
7
NH
2
2'
3
4
N
HCl
f1 (ppm)

molecules

MDPI

Commercial Sabinene ¹H 250 MHz (CDCl₃)

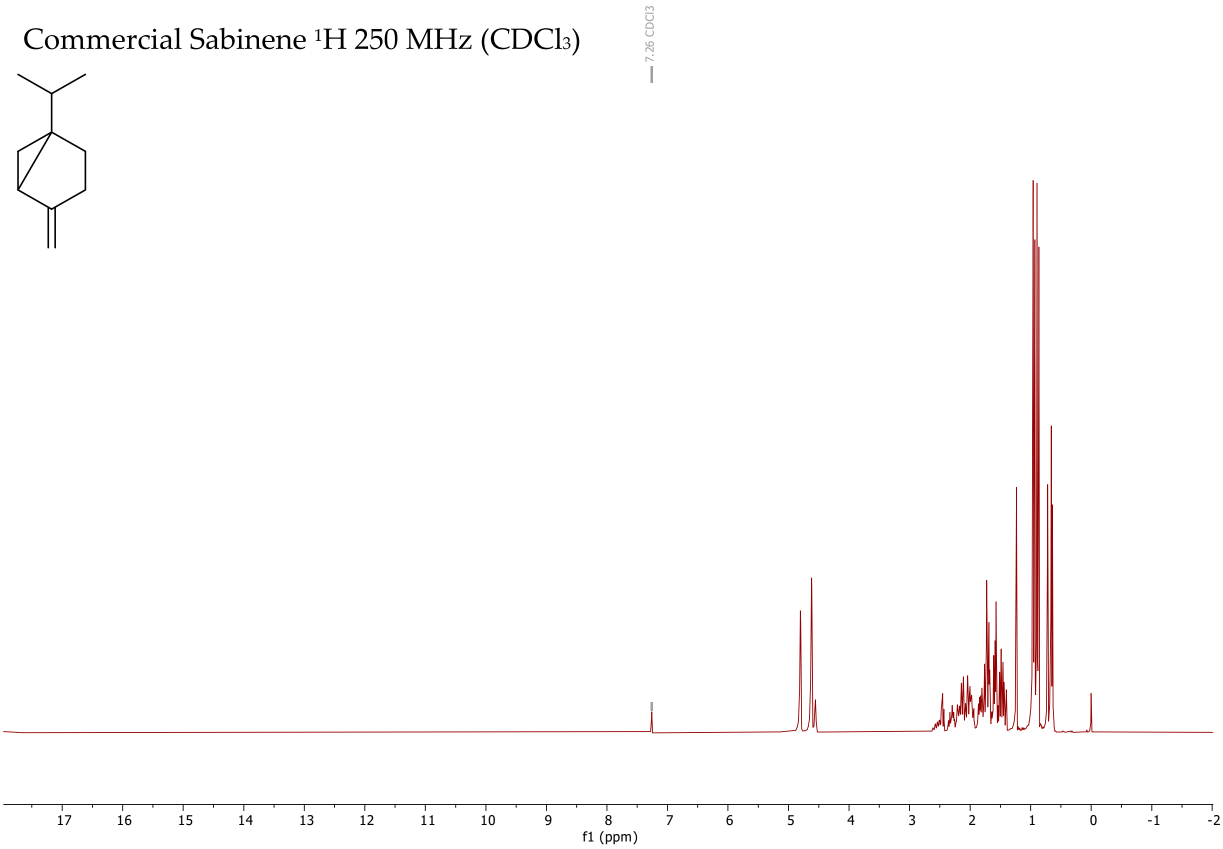

Distilled Sabinene ¹H 250 MHz (CDCl₃)

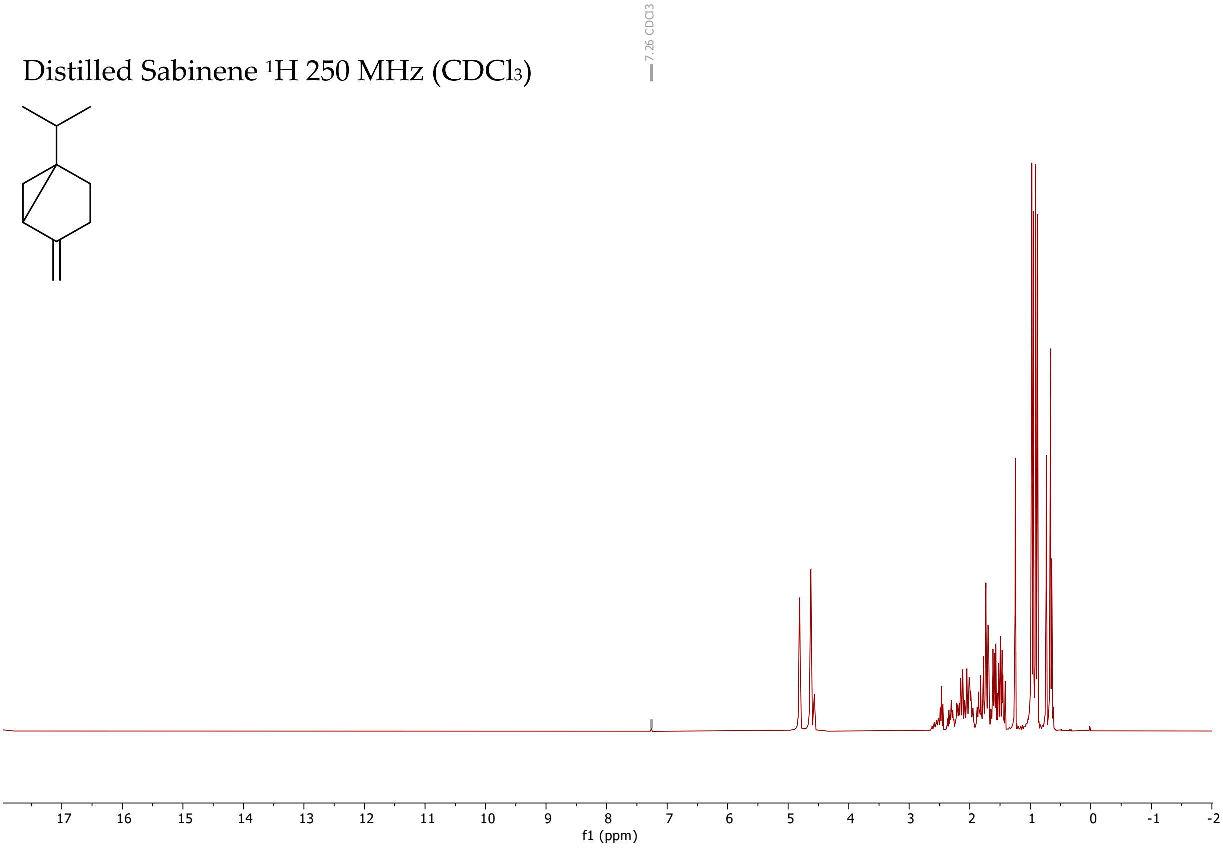

molecules

MDPI

Reference

41. Atland, H.W.; Molander, G.A. A facile synthesis of 2-aminothiazolo[5,4-*b*] and 2-aminothiazolo[4,5-*c*]pyridines. *J. Heterocycl. Chem.* **1977**, *14*, 129–134. https://doi.org/10.1002/jhet.5570140125.
